# Reclassification of the Sack-bearer Moths (Lepidoptera, Mimallonoidea, Mimallonidae)

**DOI:** 10.3897/zookeys.815.27335

**Published:** 2019-01-10

**Authors:** Ryan A. St Laurent, Akito Y. Kawahara

**Affiliations:** 1 McGuire Center for Lepidoptera and Biodiversity, Florida Museum of Natural History, University of Florida, 3215 Hull Road, Gainesville, FL 32611, USA University of Florida Gainesville United States of America; 2 Department of Biology, University of Florida, Gainesville, FL 32611, USA University of Florida Gainesville United States of America; 3 Entomology Department, University of Florida, Gainesville, FL 32611, USA University of Florida Gainesville United States of America

**Keywords:** *
Citralla
*, evolution, *
Fatellalla
*, *
Lepismalla
*, morphology, Neotropical, New World, revised classification, taxonomy

## Abstract

A backbone molecular phylogeny of Mimallonidae, based on 47 species and 515 loci, was recently published. That study resolved some of the major relationships in the family, but taxon sampling was limited and a classification of the family was not formally presented for all species. Here morphological phylogenetic analyses in parsimony and maximum likelihood (ML) frameworks were conducted that included 192 species and 55 morphological characters. A phylogenetic analysis was also conducted on the morphological dataset with a topological constraint based on the 515 locus tree from the previous study. Results show that nearly all species can be confidently placed in a genus using morphological phylogenetics. The presence of a frenulum, a character that was historically used to distinguish major groups of Mimallonidae, varies within and among genera. Based on our phylogenetic results, the classification of Mimallonidae is revised, which now includes 291 species in 41 genera. Descriptions of three new genera are included: *Fatellalla***gen. n.**, *Citralla***gen. n.**, and *Lepismalla***gen. n.** The following taxonomic changes were made in the present article: 43 new/revived combinations (in *Aceclostria* Vuillot, *Arcinnus* Herbin, *Bedosia* Schaus, *Bedosiallo* St Laurent & Kawahara, *Cicinnus* Blanchard, *Citralla*, *Druentica* Strand, *Fatellalla*, *Lacosoma* Grote, *Lepismalla*, *Mimallo* Hübner, *Procinnus* Herbin, *Psychocampa* Grote, *Roelmana* Schaus, and *Thaelia* Herbin), two new species-level synonyms (*melini* Bryk is synonymized with *viemanda* Schaus, *jaruga* Jones is synonymized with *hamata* Walker), one revived synonymy (*roscida* Dognin is resynonymized with *externa* Moore), seven new statuses (in *Druentica*, *Macessoga* Schaus, and *Trogoptera* Herrich-Schäffer), six revived statuses (in *Aceclostria*, *Cicinnus*, *Druentica*, *Psychocampa*, and *Zaphanta* Dyar), and one new designation of *nomen nudum*. In order to alleviate nomenclatural problems, twelve lectotypes are designated (for *Tolypidaamaryllis* (Schaus), *Trogopteraalthora* Schaus, *Adalgisacroesa* Schaus, *Alheitapulloides* (Dognin), *Lacosomabriasia*Schaus, *Lacosomadiederica* Schaus, *Lacosomaraydela* Schaus, *Psychocampalacuna* (Schaus), *Cicinnuscorallina* Dognin, *Cicinnuslatris* Schaus, *Cicinnussolvens* Schaus, *Cicinnustuisana* Schaus) as well as a neotype for *Mimallodespecta* Walker (= *Cicinnusdespecta*). This paper also provides apomorphies for each genus and a morphological key to genera. Annotations are given to aid researchers in understanding all changes made herein, and images of male and female and their genitalia are present for nearly all type species.

## Introduction

The sack-bearer moths (Mimallonidae) have been poorly studied in terms of their evolution, systematics, and natural history. The family is the only representative in the Mimallonoidea, and nearly all species are Neotropical (Lemaire and Minet 1998). Prior research on the family has focused largely on taxonomy of individual genera (e.g., Pearson 1951, 1984, St Laurent and Dombroskie 2016) and the only systematic treatment of the entire family was the study of Schaus (1928). Schaus described most of the taxa that are valid today, and since his work, only a handful of authors have studied the taxonomy and systematics of Mimallonidae (e.g., Pearson 1951, 1984, Franclemont 1973, Herbin 2012, St Laurent and Dombroskie 2015). Life histories of few mimallonids have been published (e.g., Dyar 1900, Wagner 2005, St Laurent et al. 2017a), and adult behavior has been poorly studied except for one diurnal North American species (St Laurent and Carvalho 2017a).

Mimallonidae have extraordinary morphological diversity. Schaus (1928) discovered the variation in the presence of the frenula across mimallonid genera and divided the family into two subfamilies based on this trait. Pearson (1951) noticed that this trait is inoconsistent within genera, and Lemaire and Minet (1998) postulated that the presence of the frenulum is a symplesiomorphy, but neither study conducted a formal phylogenetic analysis. Since Schaus’ (1928) mimallonid classification, two family-level checklists were published (Gaede 1931, Becker 1996), and in both cases, Schaus’ classification was retained with only minor taxonomic changes. Because of the significant morphological variation in Mimallonidae, studies on mimallonid taxonomy utilized features of the male genitalia to define genera (e.g., St Laurent and Dombroskie 2016, St Laurent and Herbin 2017). However, whether these characters serve as apomorphies for genera has never been formally tested because a morphological phylogenetic framework for the family has been lacking.

The only formal phylogenetic study of Mimallonidae is the molecular analysis of St Laurent et al. (2018a), which sampled 47 species. Their phylogeny was well-resolved and most clades were robustly supported. Six subfamilies and eight tribes were established or received a revived status. These authors revised the classification of several genera, proposing 19 new combinations, three new genera, and one generic synonym. However, many genus- and species-level taxonomic changes were not made because taxon sampling was limited.

The current study aims to incorporate morphological data to assess apomorphies and revise the classification of the family. This article has four main goals:

1 Conduct morphological phylogenetic analyses of Mimallonidae.

2 Present a key to genera of Mimallonidae, based on male genitalia morphology.

3 Provide a generic classification of Mimallonidae with apomorphies based on new phylogenetic results.

4 Present a complete, annotated checklist of Mimallonidae that includes all species in the family.

## Materials and methods

### Phylogenetic methods

In order build a comprehensive phylogeny of Mimallonidae (Goal 1), 192 species of Mimallonidae (66% of the family considering that 291 species are treated as valid here) were coded for 55 morphological adult characters (see Suppl. material 1: Table 1), including characters pertaining to male genitalia (25 characters), female genitalia (six characters), and external morphology (24 characters). All characters were coded as unordered, with 25 of them binary and 30 multi-state. Explanations of characters and their states can be found in Suppl. material 2, with illustrations of these characters and states in Suppl. material 3. We only included species for which we were able to dissect at least one male specimen, because male genitalia are generally the most reliable suite of characters for genus and species identification in the family (St Laurent pers. obs.). All Mimallonidae species sequenced for anchored hybrid enrichment (AHE) by St Laurent et al. (2018a) were coded for morphological characters so that a topological constraint could be applied to our morphological phylogenetic analysis. We chose this approach as morphological characters alone were not able to conclusively provide information on relationships among higher mimallonid groups (see Suppl. materials 5, 7). Two outgroups from the AHE study were also coded for morphology, the saturniid *Citheroniasplendens* (Druce), and the pyralid *Galleriamellonella* (Linnaeus).

Morphological phylogenetic analyses were carried out in a framework of maximum likelihood (ML) and parsimony. ML analyses were conducted in IQ-TREE v. 1.6.1 (Nguyen et al. 2015) using a Jukes-Cantor type model for morphological data + equal states frequency (option “-m MK+FQ”). We conducted two ML phylogenetic analyses that included morphology: 1 ML analysis of morphology that implements the higher-level topological constraint of St Laurent et al. (2018a) and 2 ML analysis of morphology that does not implement the higher-level topological constraint of St Laurent et al. (2018a). The -g option was used to perform the constrained tree search, using the dataset2_PART2 tree file from St Laurent et al. (2018a). The constraint tree topology is provided as a supplementary file (Suppl. material 4: constraint.tre). Nodal support in all IQ-TREE analyses was tested with 1000 ultrafast bootstraps (option “-bb 1000”) (Hoang et al. 2018) and SH-aLRT (option “-alrt 1000”) support.

For the parsimony analyses, we used TNT v. 1.5 (Goloboff and Catalano 2016) and conducted a New Technology Search, with default settings for sectorial, ratchet, drift, and tree fusing. Random addition sequences were set to 100 with the random seed set to 1 and all other parameters set to default. All characters were unordered and weighted equally. Nodal support was inferred using 100 jackknife replicates (Farris et al. 1996) using Traditional Search, and all other parameters set to default.

### Taxonomic key, classification, and checklist

**Figure 1.**
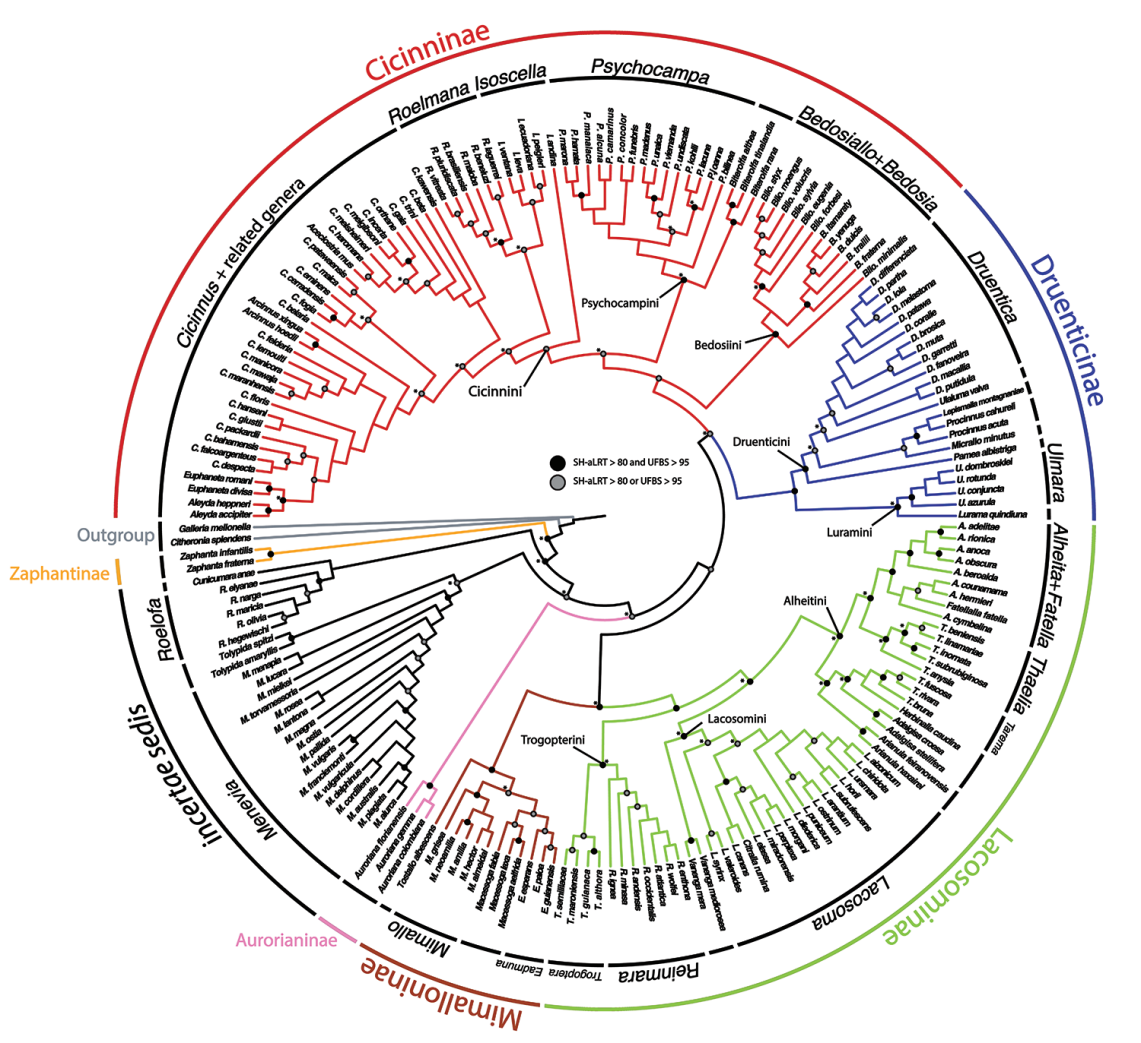
Maximum likelihood tree built using IQ-TREE with a backbone constraint (Suppl. material 4) from the phylogenomic study of St Laurent et al. (2018a). Branch lengths are removed so that relationships can be easily visualized. Asterisks denote nodes that were topologically constrained. For branch lengths and all support values, see Suppl. material 6.

Our taxonomic key (Goal 2) is based on male genitalia morphology, developed from the male genitalia characters in our morphological matrix (characters 1–25). We created this key so that researchers can readily identify genera of Mimallonidae, and place new or unrecognized taxa in the according genus. Our revised classification (Goal 3) follows the results from the topologically constrained, morphological phylogenetic analysis (Fig. 1). The section is arranged so that subfamilies are listed in phylogenetic order from the root of Mimallonidae to the crown. Tribes are listed in a consistent order below their respective subfamilies, throughout the text. Genera and species are listed alphabetically for convenience. We figure habitus of a male specimen and male genitalia of species from all genera, including those of type species when possible. Female genitalia, while often bearing genus-specific features, could not be examined extensively due to the relative rarity of female mimallonid specimens in collections. We provide images of females and their genitalia for most genera, but because we lack data for many of them, we abstain from discussing generic apomorphies based on these features.

For the checklist (Goal 4), species that were not explicitly mentioned or sampled in St Laurent et al. (2018a) have been examined morphologically in order to determine their likely placement. In addition to taxonomic information for each species in the checklist, we also include the type locality given as the country and state/department (when known), as well as the natural history collection abbreviation where primary type(s) is/are located, when known. All names considered valid in the present checklist are listed in boldface italicized type, whereas synonyms, unavailable names, and nomina nuda are given in regular italicized type. Unavailable names are denoted by the “‡” symbol, similar to the method of Fletcher and Nye (1982). Type species of each genus are signified by an asterisk.

Lectotypes are designated when syntypes from multiple localities exist for a given species, or when lectotypification is necessary to determine the identity of a species. Lectotypes for species described from syntypes from the same location, which we deem to be conspecific, will not be designated here, but rather later in more in-depth genus-level revisions where interspecific relationships can be more robustly determined. A neotype is designated for *Mimallodespecta* since the original type(s) is/are almost certainly lost (see Becker 2001).

Morphology terminology follows Kristensen (2003), Lemaire and Minet (1998), and St Laurent et al. (2018a). The term “valva apodeme” is derived from Zwick (2009). Dissections were performed as in Lafontaine (2004). Genitalia are slide mounted or stored in microcentrifuge/ microvials with glycerol. Genitalia dissection numbers are given in the legends for the respective figures, “diss.” is used as an abbreviation for “dissection”. Authorship of “St Laurent,” although occasionally published “St. Laurent,” is here standardized as “St Laurent” without the period as the author originally intended. All taxon authorships using this name should omit the period, though original article citations may include the period for standardized purposes. Furthermore, due the existence of multiple sets of different coauthors, with the first author St Laurent published in the same year, “St Laurent et al.” references are further denoted by a letter following the year, even if the “et al.” authors are not the same.

The following institutional abbreviations are used throughout the checklist.

**AMNH** American Museum of Natural History, New York, New York, USA

**ANSP** Academy of Natural Sciences, Philadelphia, Pennsylvania, USA

**CDH** Coll. of Daniel Herbin, Garidech, France

**CEIOC** Entomological Collection of the Oswaldo Cruz Institute, Rio de Janeiro, Rio de Janeiro, Brazil

**CGCM** Coll. of Carlos G. C. Mielke, Curitiba, Paraná́, Brazil

**CGD** Coll. of Guy Durand, France

**CMNH** Carnegie Museum of Natural History, Pittsburgh, Pennsylvania, USA

**CNC** Canadian National Collection of Insects, Arachnids and Nematodes, Ottawa, Ontario, Canada

**CPAC** Coleção Embrapa Cerrados, Planaltina, Distrito Federal, Brazil

**CPL** Coll. of Peter Landolt, Washington, USA

**CRAS** Coll. of Ryan St Laurent, Gainesville, Florida, USA

**CUIC** Cornell University Insect Collection, Ithaca, New York, USA

**DZUP** Coll. Pe. Jesus S. Moure, Departamento de Zoologia, Universidade Federal do Paraná, Curitiba, Paraná, Brazil

**HEC** Hope Entomological Collections of Oxford University Museum of Natural History, Oxford, U.K.

**ISEZ** The Institute of Systematics and Evolution of Animals of the Polish Academy of Sciences, Kraków, Poland

**MCZ** Museum of Comparative Zoology, Harvard University, Cambridge, Massachusetts, USA

**MGCL** McGuire Center for Lepidoptera and Biodiversity, Gainesville, Florida, USA

**MJWC** Coll. of Matthew J.W. Cock, Llannon, Wales, U.K.

**MNHN** Muséum nationale d’Histoire naturelle de Paris, Paris, France

**MNHU** Museum für Naturkunde der Humboldt-Universität zu Berlin, Germany

**MWM** Museum Witt, Munich, Germany

**NHRS** Entomological Collections, Swedish Museum of Natural History, Stockholm, Sweden

**NHMUK** Natural History Museum, London, U.K.

**NMW** Naturhistorisches Museum Wien, Vienna, Austria

**USNM** National Museum of Natural History [formerly United States National Museum], Washington, D.C., USA

**UVGC** Universidad del Valle de Guatemala, Guatemala City, Guatemala

**ZISP** Zoological Museum of the Zoological Institute of the Russian Academy of Sciences, St. Petersburg, Russia

**ZSM** Zoologische Staatssammlung München [Munich], Germany

## Results and discussion

### Phylogeny of Mimallonidae

Our morphological analysis which utilized a molecular topological constraint provides the most up-to-date picture on the evolutionary relationships of Mimallonidae (Fig. 1). This phylogeny presents many new relationships, especially within and between genera. All recognized mimallonid genera can now be placed within subfamilies and tribes (where applicable) using our phylogenetic results from this study, building on the results of St Laurent et al. (2018a). For example, all genera that were not sampled by St Laurent et al. (2018a) had their higher-level placement confirmed here. These genera include: *Cunicumara* St Laurent, as closely related to the *incertae sedis* genera *sensu*St Laurent et al. (2018a); *Eadmuna* Schaus and *Tostallo* St Laurent & C. Mielke, placed in Mimalloninae; *Tarema* Schaus in Alheitini; *Pamea* Walker and *Micrallo* St Laurent & C. Mielke in Druenticini; and *Aceclostria* Vuillot in Cicinnini.

Relationships between species were also well-supported, with essentially all species placed in the genera to which they are assigned in the present study as per the taxonomic sections of this article (Sections 3 and 4). Although the majority of genera were recovered as monophyletic in both the constrained and unconstrained ML trees (Fig. 1, Suppl. materials 5, 6), some genera were recovered as paraphyletic/polyphyletic in these analyses. Such incongruence can be summarized as follows: in both the unconstrained and constrained ML analyses *Alheita* Schaus and *Cicinnus* Blanchard were paraphyletic; *Lacosoma* Grote, *Druentica* Strand, *Procinnus* Herbin, *Psychocampa* Grote and Robinson, and *Roelmana* Schaus were only rendered paraphyletic in the unconstrained ML analysis; with *Bedosiallo* and *Isoscella* recovered as paraphyletic only in the constrained ML analysis. Despite these issues, ongoing molecular phylogenetic work continues to support the monophyly of all Mimallonidae genera as they are treated in the present article (St Laurent et al. in prep.).

Generic clade recovery in the unconstrained ML analysis (Suppl. material 5) and parsimony analysis (Suppl. material 7) were not significantly conflicting with results from our ML constrained analysis, with the fundamental differences lying in the backbone topology that was constrained by molecular data. There were no major genus-defining nodes, that is, clades which include all representatives of a given genus, that were recovered with UFBS ≥ 95 and SH-aLRT ≥ 80 in the unconstrained ML analysis that were not also recovered in the constrained ML analysis.

Our results also demonstrate that morphological traits, such as the degree of development of the frenulum (Schaus 1928), is not a diagnostic character to distinguish subfamilies due to the variability of this character within genera (particularly in *Mimallo* and *Druentica*). Other seemingly robust external characteristics that would appear diagnostic of genera, such as the presence or absence of hyaline patches, antennae formation (filiform, dentate, bipectinate), also vary within genera (e.g., antennae structure within *Roelofa* Schaus). We note that one of the autapomorphies of Mimallonidae given by Lemaire and Minet (1998: 323): “distal section of male antenna provided with short rami (distinctly shorter than those of the proximal section) or even wholly devoid of rami” should be revalidated because this degree of rami shortening along male antennae is quite variable across Mimallonidae.

### Key to genera of Mimallonidae

**Table:** 

1	Uncus deeply bifurcated, bifurcation deeper than uncus width (Fig. 4a)	***Tolypida* Schaus**
–	Uncus not deeply bifurcated, but may be bidentate if not simple	**2**
2	Genitalia structurally asymmetrical, such that phallus and single long tusk are oriented to one side of genitalia (Fig. 33a)	***Aceclostria* Vuillot**
–	Genitalia largely symmetrical (minor asymmetry may be apparent in size of projections from the ventral portion of the vinculum, or in characters related to the valvae) but not with major structural asymmetry as in Fig. 33a	**3**
3	Uncus proximal margin continuous with tegumen (Fig. 9a)	***Macessoga* Schaus**
–	Uncus proximal margin clearly differentiated from tegumen (all male genitalia figures herein, except Fig. 9a)	**4**
4	Vinculum apodemes present (e.g., Fig. 35c)	**5**
_	Vinculum apodemes absent	**11**
5	Vinculum tusks present (Figs 31, 35b, 37b, 38b, 39b)	**6**
–	Vinculum tusks absent (Fig. 36)	***Cicinnus* Blanchard, in part**
6	Vinculum tusks present as single pair (Figs 31, 35b, 37b)	**8**
–	Vinculum tusks present as two pairs (Figs 38b, 39b)	**7**
7	Base of gnathos rectangular, relatively lightly sclerotized (Fig. 38a)	***Isoscella* St Laurent and Carvalho**
–	Base of gnathos ovoid, relatively heavily sclerotized (Fig. 39a)	***Roelmana* Schaus**
8	Vinculum tusks sharp (Fig. 35b)	**9**
–	Vinculum tusks blunt (Fig. 37b)	***Arcinnus* Herbin**
9	Juxta complicated or hood-shaped, fused to phallus, occupying most of central region of genitalia (e.g., Figs 35a, 36b)	**10**
–	Juxta simple, fused to base of phallus, anchoring it to vinculum, without complex dorsal region (Fig. 34b)	***Aleyda* Schaus**
10	Juxta component dorsal to phallus distinctly hood-like, arching over phallus towards its terminus (Fig. 35a)	***Euphaneta* Schaus**
–	Juxta component dorsal to phallus variable, but always complex and heavily sclerotized, not hood-like, usually with setae covered fan-like lobes symmetrically on either dorsal side of phallus (e.g., Fig. 36b)	***Cicinnus* Blanchard, in part**
11	Lobe-like ventral-anterior projection of the vinculum present (Fig. 31d)	***Bedosia* Schaus**
–	Lobe-like ventral-anterior projection of the vinculum absent (vinculum base in most Mimallonidae truncated or flattened, not extended as mesal lobe)	**12**
12	Base of valvae with smooth, elongate or curled spine-like tusks (Figs 22a, 23, 24a, 25b, 26), not to be confused with extensions from valvae apodemes (see below) or vincular tusks of previous couplets	**13**
–	Base of valvae without elongate or curled spine-like tusks (not including saccular projections/spines)	**18**
13	Valvae tusks curled, often shorter than half length of valva (Figs 25b, 132II)	**14**
–	Valvae tusks not curled, though they may be curved, generally elongate, longer than half length of valva (Figs 22a, 23, 24a, 26)	**15**
14	Gnathos arms massive, heavily sclerotized, much larger than uncus, extending beyond saccular edge of valva (Fig. 132I)	***Lepismalla* gen. n.**
–	Gnathos arms very small, smaller in size than uncus, not extending beyond saccular edge of valva (Fig. 25b)	***Pamea* Walker**
15	Gnathos present	**16**
–	Gnathos absent	***Ulaluma* St Laurent & Kawahara**
16	Valvae membranous ventrally, extensively narrowed distally, and clubbed distally (Fig. 23a)	***Micrallo* St Laurent & C. Mielke**
–	Valvae not as above	**17**
17	Sternite VIII with claw-like spines (Fig. 26a)	***Procinnus* Herbin**
–	Sternite VIII without claw-like spines (but pair of elongate arms and/or other modifications may be present) (e.g., Fig. 22)	***Druentica* Strand**
18	Uncus broadly rectangular (Fig. 106I)	***Fatellalla* gen. n.**
–	Uncus variable, usually triangular, may be somewhat flattened or finger-like apically, but basally triangular	**19**
19	Pronounced coecum phallus absent (Figs 20d, 21)	**39**
–	Pronounced coecum phallus present (e.g., Figs 5c, 7, 18d)	**20**
20	Coecum phallus simple (not bifurcated) (Fig. 18d)	**22**
–	Coecum phallus bifurcated (Fig. 5c)	**21**
21	Transtilla/valvae apodemes forming complex sclerotization situated inward into body cavity from which elongated spine-like tusks project outward through center of genitalia (Fig. 3a)	***Menevia* Schaus**
–	Transtilla/valvae apodemes not developed as above, elongated spine-like tusks absent (but saccular spines present)	***Cunicumara* St Laurent**
22	Phallus snake-like (narrow, curved, variable in length) (Fig. 7a; Suppl. material 3, Plate 6, 20:0), gnathos as pair of massive, heavily sclerotized triangular processes or as two bifurcated processes (Fig. 7, Suppl. material 3, Plate 1, 5:8)	***Auroriana* St Laurent & C. Mielke**
–	Phallus and gnathos (if present) not as above	**23**
23	Distal tip of valvae extremely narrowed, forming spine (Fig. 17a), does not include saccular spines or mesal valva spines	***Arianula* Herbin**
–	Distal margin of valvae variable, but not as above	**24**
24	Gnathos absent (note, valvae apodeme extensions may be confused with gnathos)	**25**
–	Gnathos present (note gnathos may be typical with paired projections (e.g., Figs 12, 18c, 19a) or a simple plate below the uncus (e.g., Figs 13a, 14b), or a plate non-synscleritous with surrounding genitalia (Fig. 15b)	**29**
25	Valvae apodemes lobe-like, covered in setae (Fig. 27b)	***Lurama* Schaus**
–	Valvae apodemes not as above	**26**
26	Valva apodeme/transtilla extensions columnar, broad, spanning near entire dorsal-ventral height of vinculum (Fig. 11a)	***Tostallo* St Laurent & C. Mielke**
–	Valvae apodemes not as above	**27**
27	Valvae apodemes filamentous, elongate (Fig. 10a)	***Mimallo* Hübner**
–	Valvae apodemes not as above	**28**
28	Valvae apodemes wrinkled triangular projections (Fig. 8a)	***Eadmuna* Schaus**
–	Valvae apodemes not as above	***Thaelia* Herbin, in part**
29	Diaphragm with four discrete setae-filled sacks (upper pair of sacks may be significantly smaller and/or thicker than lower pair) (Figs 18, 19)	**30**
–	Diaphragm without four discrete setae-filled sacks	**31**
30	Gnathos distally separated as pair of finger-like projections, or slight bifurcation (Fig. 18c)	***Reinmara* Schaus**
–	Gnathos distally a single finger-like projection, not separated distally (Fig. 19a)	***Trogoptera* Herrich-Schäffer**
31	Gnathos with paired distal projections (e.g., Figs 6, 29c, 30a, 32a)	**35**
–	Gnathos with single projection, or not projected (often plate-like) (Figs 2a, 13a, 15b)	**32**
32	Uncus apex and gnathos both significantly more heavily sclerotized than surrounding tegumen and situated very near each other, appearing pincer-like from the lateral aspect (Fig. 2a)	***Zaphanta* Dyar**
–	Uncus and gnathos (if present) variable, not as above	**33**
33	Gnathos as single mesal plate, not synscleritous with surrounding genitalia (Fig. 15b)	***Herbinalla* St Laurent & Kawahara**
–	Gnathos not as above	**34**
34	Gnathos projected mesally, heavily sclerotized, forming closed region below uncus (Fig. 16a)	***Thaelia* Herbin, in part**
–	Gnathos unfused mesally or fused, but not heavily sclerotized/mesally projected (Fig. 28a)	***Ulmara* Schaus**
35	Phallus sword-like, sharp, covered in minute spines (Fig. 12a)	***Adalgisa* Schaus**
–	Phallus not as above: narrow, tubular (Fig. 32), roughly cylindrical (e.g., Fig. 6, 14, 20, 21, 30), and bent or somewhat flattened (Fig. 29)	**36**
36	Either side of phallus flanked by knob-like sclerotization of juxta, these knobs usually only attached to phallus by membrane, not strongly fused to it by sclerotization (Figs 29b, 30)	**37**
–	Either side of phallus not flanked by knob-like sclerotization of juxta	**38**
37	Gnathos arms with minute teeth ventrally, or with short protuberance near apex (Fig. 29c)	***Biterolfa* Schaus**
–	Gnathos arms either wrinkled, or elongated, finely tapering distally, never toothed or with additional dorsal protuberances (Fig. 30a)	***Psychocampa* Grote**
38	Mesal base of valvae with upturned projection, this projection usually spined (Fig. 6a)	***Roelofa* Schaus**
–	Mesal base of valvae simple	***Bedosiallo* St Laurent & Kawahara**
39	Gnathos replaced by smooth, flattened plate (Figs 13a, 14b); no discernable gnathos projections evident.	**42**
–	Gnathos either apparently absent, or reduced to pair of narrow sclerotizations extending below uncus, from which various projections may emanate (Figs 20b, 21b), this gnathos configuration is generally synscleritous with the juxta, which too is complicated and fused to phallus	**40**
40	Uncus broadly triangular (Fig. 118a), not sharp apically or narrowed distally	***Citralla* gen. n.**
–	Uncus very narrow, sharp, or more acutely triangular	**41**
41	Gnathos synscleritous with juxtal complex and/or connected to diaphragm by patches of setae (Fig. 20b), variable in configuration with variable extensions, subuncal projections also present (Fig. 20a)	***Lacosoma* Grote**
–	Gnathos reduced to finely tapering sclerotizations extending downward below subuncal projections, therefore uncus/gnathos complex not synscleritous with juxta	***Vanenga* Schaus**
42	Phallus with elongated lateral projections ending in sharp tooth-like points (Fig. 14c)	***Tarema* Schaus**
–	Phallus variable, but not as above, never with elongated, sharp-tipped lateral projections (Fig. 13)	***Alheita* Schaus**

### Generic classification of Mimallonidae

For the generic classification of Mimallonidae, we follow the phylogeny in Fig. 1. Subfamilies are in phylogenetic order from the root of Mimallonidae to the crown in this tree, with genera listed alphabetically. For each genus, a brief diagnosis is given, allowing recognition of species belonging to the genus based on external morphology and maculation. Apomorphies based on male genitalia are provided to formally identify the genus and allow additional new species to be placed accurately in the genera as we have defined them. Three new monotypic genera, *Fatellalla* gen. n., *Citralla* gen. n., and *Lepismalla* gen. n., are newly described and therefore also include formal descriptions.

### Zaphantinae St Laurent & Kawahara, 2018

#### Zaphanta

Taxon classificationAnimaliaLepidopteraMimallonidae

Dyar, 1910

**Figures 2–7.**
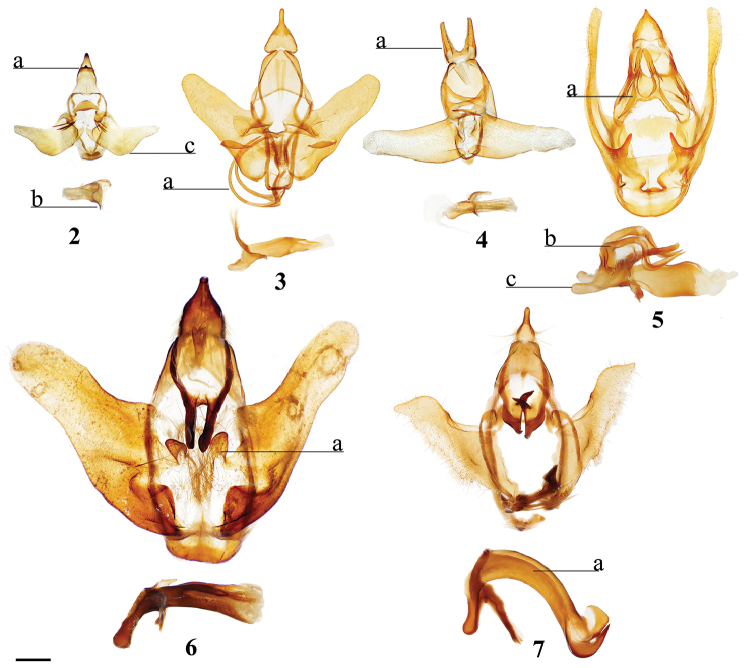
Ventral view of male genitalia of type species of genera belonging to Zaphantinae, *incertae sedis*, and Aurorianinae. Phallus/juxtal complex is figured below the genitalia in the lateral view. **2***Zaphantainfantilis*, St Laurent diss.: CPC 4 [reused with permission from St Laurent et al. (2018a), Systematic Entomology] (CRAS) **3***Menevialantona*, St Laurent diss.: 3-7-15:2 [reused with permission from St Laurent and Dombroskie (2016), ZooKeys] (CMNH) **4**Tolypidacfamaryllis, St Laurent diss.: LEP53357 (MGCL) **5***Cunicumaraanae* holotype, St Laurent diss.: 3-14-16:6 [reused with permission from St Laurent (2016), ZooKeys] (CMNH) **6***Roelofaolivia*, St Laurent diss.: 8-17-17:2 (CRAS) **7***Aurorianacolombiana* holotype, St Laurent diss.: 11-3-15:1 [reused with permission from St Laurent and Mielke (2017), ZooKeys] (NHMUK). Scale bar: 1 mm.

Figs 2 , 40 , 75 , 76

##### Type species.

*Zaphantainfantilis* Dyar, 1910.

##### Diagnosis.

Very small moths, among the smallest in body size within Mimallonidae, with forewing length ranging from 9–11 mm. Coloration also diagnostic: wing ground color yellow with purplish-pink antemedial regions. Ventrum of both fore- and hindwings with apparent antemedial as well as postmedial lines.

##### Apomorphy.

(1) Uncus and a subuncal sclerotization fused, heavily sclerotized apically forming a conical structure with tegumen (Fig. 2a).

#### Incertae Sedis

##### Cunicumara

Taxon classificationAnimaliaLepidopteraMimallonidae

St Laurent, 2016

Figs 5 , 77

###### Type species.

*Cunicumaraanae* St Laurent, 2016.

###### Diagnosis.

Hoary appearance caused by interspersion of gray, pale-khaki, and dark-brown scales layered upon salmon to orange-brown, sandy tan ground color. Extremely long bipectinate antennae extending more than half length of the stout forewings, with distinctly long pectinations.

###### Apomorphies.

(1) Basally-fused and outwardly projected gnathos with paired distal extensions (Fig. 5a); (2) Phallus with dorsal, curled, horn-like juxtal processes with third, single process located between the curled pair (Fig. 5b).

###### Remarks.

St Laurent et al. (2018a) did not include this genus in their phylogeny and instead treated this genus as *incertae sedis* based on morphology, due to similarities with the other *incertae sedis* genera. The morphological phylogenetic results of the present article support a close relationship between *Cunicumara* and other *incertae sedis* genera, particularly with *Menevia* and *Roelofa* (Fig. 1, see also Suppl. materials 5–7).

The female of *C.anae* is unknown, and thus we are unable to figure it or its genitalia.

##### Menevia

Taxon classificationAnimaliaLepidopteraMimallonidae

Schaus, 1928

Figs 3 , 41 , 79 , 80 ; Suppl. material 3: Plates 1, 3, 4, 6

###### Type species.

*Cicinnuslantona* Schaus, 1905.

###### Diagnosis.

Shape somewhat variable, but maculation consistent in presence of white apical dash on the forewing which forms a connection with a white, swooping “postmedial lunule” which may or may not form a continuous white band outlining the postmedial line (St Laurent and Dombroskie 2016).

###### Apomorphy.

(1) Paired, elongated, thin, tusk-like extensions reaching outwards from modified transtilla/juxtal complex which itself extends inward into the body cavity from attachments on either side of the inner costal apodemes of the valvae (Fig. 3a).

##### Roelofa

Taxon classificationAnimaliaLepidopteraMimallonidae

Schaus, 1928

Figs 6 , 43 , 81 , 82 ; Suppl. material 3: Plate 3

###### Type species.

*Perophoraolivia* Schaus, 1896.

###### Diagnosis.

Combination of the following two characters: forewing with black apical streak which forms a connection with a dark colored, straight or concave (or convex in some females) postmedial line and with pair of elongate darkly colored terminal abdominal tufts. Such tufts are seen in other genera but never in combination with the presence of a darkly colored forewing apical streak.

###### Apomorphy.

Valvae with basal mesal flap-like extension which may be finger-like or more triangular and spined; valva flap extends into center of genitalia and curves outwards (Fig. 6a).

##### Tolypida

Taxon classificationAnimaliaLepidopteraMimallonidae

Schaus, 1928

Figs 4 , 42 , 83 , 84 , 159

###### Type species.

*Hydriasamaryllis* Schaus, 1896.

###### Diagnosis.

Unmistakable yellow moths with thick gray ante- and postmedial bands spanning width of the wings, gray bands are outlined with cream or white on both sides.

###### Apomorphy.

Deeply bifid uncus such that bifurcation deeper than uncus width (Fig. 4a).

### Aurorianinae St Laurent & Kawahara, 2018

#### Auroriana

Taxon classificationAnimaliaLepidopteraMimallonidae

St Laurent & C. Mielke, 2016

Figs 7 , 44 , 78 ; Suppl. material 3: Plates 1, 2, 4, 6

##### Type species.

*Aurorianacolombiana* St Laurent & C. Mielke, 2016.

##### Diagnosis.

Orange-brown ground color with diffuse pink coloration on all regions of wings, with a tornal notch on the forewing only, anterior margin of the hindwing smooth. Similarly colored brown and pink Mimallonidae have a notch on the anterior margin of the hindwing (though this may be weak) as well as a usually deeper notch on the forewing tornus, and/or the pink coloration is not suffused across the whole wing in these species, but rather clearly delimited by the postmedial line. For example, see *Fatellalla* gen. n. below, a similarly colored genus with more clearly distinct medial and submarginal pink coloration.

##### Apomorphy.

Distally downward curved, snake-like phallus (Fig. 7a; Suppl. material 3: Plate 6, 20:0).

### Mimalloninae Burmeister, 1878

#### Eadmuna

Taxon classificationAnimaliaLepidopteraMimallonidae

Schaus, 1928

**Figures 8–11.**
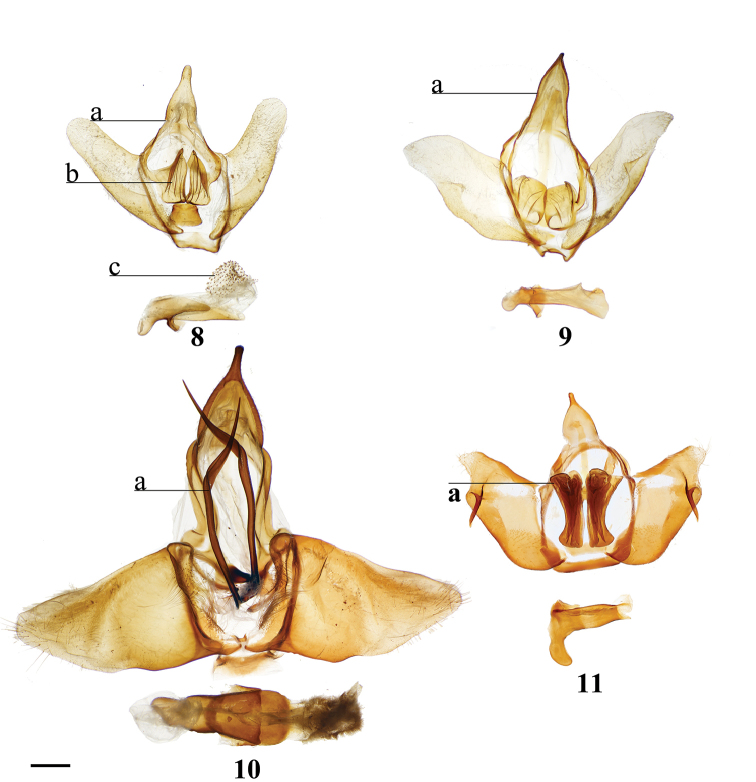
Ventral view of male genitalia of type species of genera belonging to Mimalloninae. Phallus/juxtal complex is figured below the genitalia in the lateral view, except where noted. **8***Eadmunaesperans*, St Laurent diss.: 3-17-18:6 (CRAS) **9**Macessogacffabia, St Laurent diss.: CPC 1 (CRAS) **10***Mimalloamilia*, St Laurent diss.: 10-5-17:8, phallus oriented dorsally [reused with permission from St Laurent et al. (2018a), Systematic Entomology] (CRAS) **11***Tostalloalbescens*, St Laurent diss.: 10-21-15:4 [reused with permission from St Laurent and Mielke (2016), ZooKeys] (NHMUK). Scale bar: 1 mm.

Figs 8 , 45 , 85 , 86

##### Type species.

*Cicinnusesperans* Schaus, 1905.

##### Diagnosis.

Combination of the following characters: silvery-gray or brown ground color, forewing with hyaline or sub-hyaline patch bisected by the M2 vein; dorsal postmedial line incomplete, formed by brown crescents between veins; the presence of smooth wing margins without a sharply falcate forewing apex.

##### Apomorphies.

Combination of the following characters: (1) Uncus clearly differentiated from tegumen as separate smaller structure (Fig. 8a) compared to single elongate triangular uncus + tegumen with little differentiation between these parts; (2) Triangular transtilla or valva apodeme (of uncertain homology) extensions finely wrinkled along their length (Fig. 8b); (3) Vesica with distinct cornutus (which itself may be formed by many cornuti fused into one) or scobinate patch (Fig. 8c).

##### Remarks.

St Laurent et al. (2018a) did not include this genus in their phylogeny and instead placed this genus in Mimalloninae based on morphology, due to clear similarities to the genus *Macessoga*, a genus that was included. Our ongoing molecular work, which includes broadened taxon coverage of Mimallonidae, including *Eadmuna*, fully supports this placement (St Laurent et al. in prep.). Additionally, all of our morphological phylogenetic results consistently place *Eadmuna* as sister to *Macessoga* (Fig. 1, see also Suppl. materials 5–7), therefore confirming the placement of this genus in Mimalloninae.

#### Macessoga

Taxon classificationAnimaliaLepidopteraMimallonidae

Schaus, 1928

Figs 9 , 46 , 87 , 88 ; Suppl. material 3: Plate 2

##### Type species.

*Perophorafabia* Druce, 1887.

##### Diagnosis.

Similar to *Eadmuna*, but ground coloration more yellow to yellow-brown, with continuous dorsal postmedial line, which is mostly straight (except for a sharp angle toward costa), not incomplete and crenulate as in *Eadmuna*.

##### Apomorphies.

Combination of the following characters: (1) Uncus undifferentiated from tegumen, forming large (relative to remainder of genitalia) rounded triangle (Fig. 9a; Suppl.material 3: Plate 2); (2) Triangular transtilla or valva apodeme (of uncertain homology) extensions similar to those of *Eadmuna* but irregularly shaped and wrinkled; (3) Vesica without distinct cornutus or scobinate patch.

#### Mimallo

Taxon classificationAnimaliaLepidopteraMimallonidae

Hübner, 1820

Figs 10 , 47 , 89 , 90 ; Suppl. material 3: Plate 3

##### Type species.

*Bombyxamilia* Cramer, 1780.

##### Diagnosis.

Combination of the following characters: wing margins irregular, postmedial lines always with distinct maculation of variable thickness spanning from postmedial line to wing margin, postmedial maculation wider on hindwing; forewing always with hyaline patch bisected by M2.

##### Apomorphy.

Pair of twisted, wrinkled, tendril-like elongations originating from vinculum near inner costal base of valvae (Fig. 10a), forming complex with valva apodeme, elongations extend inward into body cavity and then curve outward through center of vinculum, elongations’ total length (straightened) longer than valva or tegumen + uncus.

#### Tostallo

Taxon classificationAnimaliaLepidopteraMimallonidae

St Laurent & C. Mielke, 2016

Figs 11 , 47 , 91 , 92

##### Type species.

*Perophoraalbescens* Jones, 1912.

##### Diagnosis.

Unmistakable, bird dropping-like white and brown coloration, white ground color combined with rounded forewings is unique to this genus.

##### Apomorphy.

Transtilla/valva apodeme formed by two columnar structures with multiple invaginations and internal wrinkles (Fig. 11a).

##### Remarks.

*Tostallo* was not included in St Laurent et al. (2018a), but was placed in Mimalloninae based on morphology due to the presence of all apomorphies of the subfamily. Furthermore, the shape of the uncus in *Tostallo* is nearly identical those of some *Mimallo* and *Eadmuna*, and the transtilla/valva apodeme configuration is similar to this structure these other genera as well. Our morphological phylogenetic analyses, except the unconstrained ML analysis, robustly support the inclusion of *Tostallo* in Mimalloninae, and we consider this evidence to confirm the subfamily placement of this unique genus. Ongoing molecular phylogenetics of Mimallonidae, which include *Tostallo*, support the placement of this genus in Mimalloninae (St Laurent in prep.).

### Lacosominae Dyar, 1893

#### Trogopterini St Laurent & Kawahara, 2018

##### Reinmara

Taxon classificationAnimaliaLepidopteraMimallonidae

Schaus, 1928: 654

Figs 18 , 54 , 107 , 108 ; Suppl. material 3: Plates 4, 6

###### Type species.

*Cicinnusenthona* Schaus, 1905.

###### Diagnosis.

*Reinmara* can be recognized by the following combination of characters: contrast between medial and submarginal areas due to diffuse pink or silvery-gray scaling delimited by strongly marked, relatively straight postmedial line; notched forewing tornus and anterior margin of hindwing. In all but one species (*T.ignea* St Laurent, Herbin, & C. Mielke) female and male antennae are strongly dimorphic, being bipectinate in males as is typical of Mimallonidae and dentate in females. Such dimorphism is only also observed in unrelated *Roelofa*.

###### Apomorphies.

Combination of the following characters: (1) Saccular edge of valva curled, with short spine of variable length (Fig. 18a); (2) Bulbous, asymmetrically sized setae-filled diaphragmal sacs (Fig. 18b); (3) Gnathos mesally fused and bifid with fingerlike tips distally (Fig. 18c).

##### Trogoptera

Taxon classificationAnimaliaLepidopteraMimallonidae

Herrich-Schäffer, [1856]

Figs 19 , 55 , 109 , 110 , 160

###### Type species.

*Trogopteraerosa* Herrich-Schäffer, [1856].

###### Diagnosis.

Similar in overall size and shape to *Reinmara*, but wings broader, margins more squared, ground coloration more earthen in tone, most species are light khaki brown, some darker. Males and females with bipectinate antennae.

###### Apomorphies.

Apomorphies 1 and 2 as for *Reinmara*; but gnathos not bifid, distally forming single point of various lengths (Fig. 19a).

###### Remarks.

Although the morphology of *Reinmara* and *Trogoptera* are similar, we note that in ongoing molecular phylogenetics of the family, which now includes denser sampling of both genera than were available in St Laurent et al. (2018a), the two genera are quite divergent genetically. Eventually it will be worthwhile to include *R.ignea* which displays characters found in both genera (*Reinmara* genitalia but *Trogoptera* antennae structure) in order to clarify the phylogeny of Trogopterini.

#### Alheitini St Laurent & Kawahara, 2018

##### Adalgisa

Taxon classificationAnimaliaLepidopteraMimallonidae

Schaus, 1928

**Figures 12–17.**
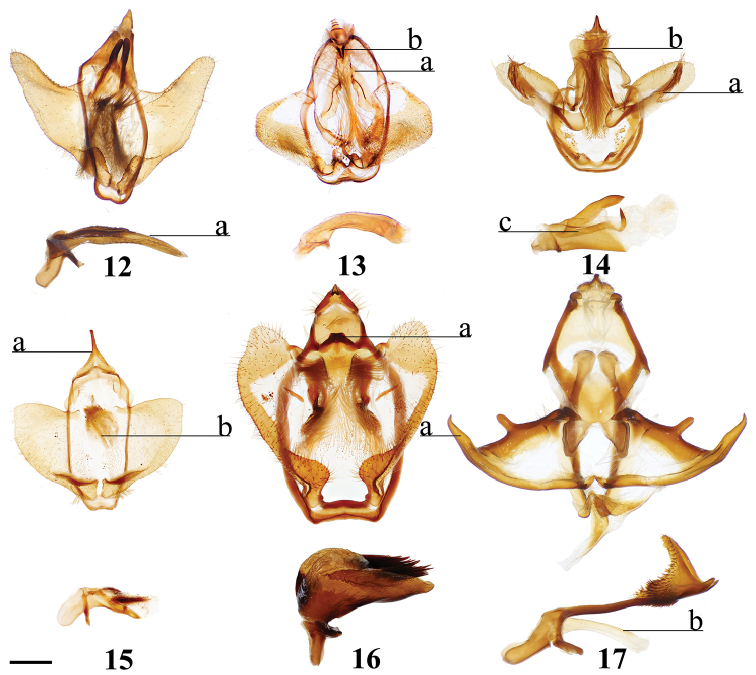
Ventral view of male genitalia of type species (except where noted) of genera belonging to Lacosominae: Alheitini. Phallus/juxtal complex is figured below the genitalia in the lateral view. **12***Adalgisacroesa*, St Laurent diss.: 9-20-17:1 (CRAS) **13***Alheitarionica*, St Laurent diss.: 3-27-17:2, not the type species of *Alheita* [reused with permission from St Laurent et al. (2018a), Systematic Entomology] (CRAS) **14***Taremarivara*, St Laurent diss.: 3-14-16:7 [reused with permission from St Laurent et al. (2017b), ZooKeys] (CNC) **15***Herbinallacaudina*, St Laurent diss. 10-8-14:1 [reused with permission from St Laurent et al. (2018a), Systematic Entomology] (CUIC) **16**Thaeliacfbeniensis, St Laurent diss.: 4-5-16:1, not the type species of *Thaelia* (USNM) **17***Arianulahaxairei*, St Laurent diss.: LEP50135 [reused with permission from St Laurent et al. (2018a), Systematic Entomology] (MGCL). Scale bar: 1 mm.

Figs 12 , 49 , 93 , 94 , 161

###### Type species.

*Adalgisacroesa* Schaus, 1928.

###### Diagnosis.

Three distinct hyaline patches between the following vein pairs of the forewing: Rs2 and Rs3, M3 and CuA1, CuA1 and CuA2. There are smaller hyaline patches between most other wing veins and narrowly along distal margin of discal cell. Similarly located, smaller hyaline patches exist on the hindwing as well.

###### Apomorphy.

(1) Phallus very long (longer than length from base of vinculum to tip of valva) and sharp, covered in fine spine-like setae (Fig. 12a), Suppl. material 3: Plate 6, 24: 4.

##### Alheita

Taxon classificationAnimaliaLepidopteraMimallonidae

Schaus, 1928

Figs 13 , 50 , 101 , 162

###### Type species.

*Cicinnusanoca* Schaus, 1905.

###### Diagnosis.

Small mimallonids with consistently short, triangular wings, always with a white postmedial lunule on the forewing, though this may be faint in species with accented veins in the broad, darker postmedial region (a character which itself is rather diagnostic of *Alheita*). Ventrally largely unmarked except for discal spot and significantly darker brown region of forewing delimited by the outline of the dorsal postmedial lunule. The similar genus *Tarema* is not so cleanly marked ventrally and has less falcate forewings.

###### Apomorphies.

(1) Gnathos plate typical of Alheitini, but with a thickly sclerotized mesal band that runs along it vertically (Fig. 13a); (2) Uncus rounded with paired mesal teeth (Fig. 13b).

###### Remarks.

We were unable to include a female *A.anoca* in our figures. Female *Alheita* are generally not largely distinct from males, with only the usual degree of sexual dimorphism (larger size, broader wings) which is usually observed in related genera.

##### Arianula

Taxon classificationAnimaliaLepidopteraMimallonidae

Herbin, 2012

Figs 17 , 95 , 96

###### Type species.

*Arianulahaxairei* Herbin, 2012.

###### Diagnosis.

*Arianula* is the only other mimallonid genus besides *Adalgisa* which has irregularly scattered hyaline patches on all wings. However, *Arianula* is easily recognized by the alternate configuration of these hyaline patches, such that there are fewer patches on the forewing than in *Adalgisa*, with the most apparent patch being between Rs4 and M1 (conversely in *Adalgisa*, the most apparent patch is between Rs2 and Rs3); and a unique trio of rectangular patches on the hindwing between M3, CuA1, and CuA2, not observed in this arrangement in *Adalgisa*.

###### Apomorphies.

(1) Bizarre claw-like valvae each similar in size to the tegumen + uncus, giving the overall genitalia a triangular appearance (Fig. 17a); (2) Phallus weakly sclerotized, tube-like (Fig. 17b), with elongated narrow juxtal process dorsal to phallus, which itself terminates in a multi-pronged, spined upward curved claw.

##### Fatellalla

Taxon classificationAnimaliaLepidopteraMimallonidae

St Laurent & Kawahara gen. n.

http://zoobank.org/31376FA5-D716-4DA5-ACA7-9461DE78982C

Figs 104–105 , 106

###### Type species.

*Cicinnusfatella* Schaus, 1905: 326, by present designation.

###### Etymology.

The name for this new genus is derived from the type, and only known species belonging to *Fatellalla*: *Fatellallafatella* comb. n. The name is feminine.

###### Diagnosis.

This new genus can be recognized by the following combination of characters in the male (the only known sex): triangular forewings with pink antemedial and medial areas, which contrast with the orange-brown submarginal area. The submarginal area is clearly delimited by a pair of thin, straight, preapical, blackish brown postmedial lines. The rounded hindwings display the same patterning as the forewings. The coloration of the body is that of the ground color of the wings: orange-brown, with a distinct darker brown lateral line along either side of the abdomen, as well as a distinct dark brown tuft of elongated scales extending from the terminus of the abdomen. The genitalia are distinct due to the truncated, flattened uncus and the knob-like saccular process at the base of each valva, characters together not observed in any other known Mimallonidae. The gnathos plate is typical of alheitine Lacosominae; and the short, rounded valvae, narrow pair of valva apodeme arms, and thick bunch of elongate setae originating from the diaphragm are similar to those seen in *Tarema* and *Alheita*. In *Fatellalla*, the arms cross over each other mesally and are sharply tipped unlike in these other genera.

###### Apomorphies.

(1) Uncus flattened, truncated (Fig. 106I); (2) Sharply tipped valva apodeme arms which cross mesally below uniquely shaped (mesally rounded) gnathos plate (Fig. 106II).

###### Description.

**Male.***Head*: Tan-brown ground color, frons dark brown, eyes very large, occupying more than two-thirds area of head, bordered posteriorly by dark scales; antenna coloration dark tan, antenna almost entirely bipectinate, distalmost 9–10 antennomers dentate; labial palpus three segmented. *Thorax*: Coloration pinkish orange with red hue (fading to light brown in old specimens), scales covering prothorax grayer, contrasting against lighter remainder of thorax. *Legs*: Coloration as for thorax, vestiture thick, long. Tibial spurs elongate, covered in scales except for tip, roughly half length of first tarsal segment. *Forewing dorsum*: Forewing length: 15.5–17.0 mm, avg.: 16.3 mm, wingspan: 29.0–4.5 mm, n = 2. Triangular, margin mostly straight except slightly convex below apex and slight indentation at tornus. Ground color orange brown, but antemedial and medial areas evenly tinted with pink, faint gray suffusion present submarginally. Antemedial line absent; preapical postmedial line formed by two thin, parallel dark brown lines with medial area pink coloration between them. Postmedial lines slightly wavy basally. Pink coloration of medial area does not extend beyond postmedial lines. Discal mark present as pink ovoid region slightly lighter than surrounding medial area. Fringe poorly preserved in examined specimens, but darker brown than submarginal area. *Forewing ventrum*: Darker brown than forewing dorsum, medial pink suffusion bleeds into submarginal area, postmedial line convex and indented at intersections with veins. *Hindwing dorsum*: Coloration, patterning as for forewing dorsum, but discal mark absent, submarginal area comparatively wider than medial area. *Hindwing ventrum*: Following same pattern as forewing ventrum, but lighter overall. Frenulum present as single bristle. *Venation*: Typical of Mimallonidae. *Abdomen*: Dorsal coloration as for thorax, laterally very dark brown, nearly black, dark coloration continues on to elongated paintbrush-like tuft of dark-brown scales extending from terminus of abdomen. Vestiture thick, long. *Genitalia*: (Fig. 106) n= 4. Vinculum ovoid, ventrally rounded. Paired sclerotized arms extend outward from sclerotized base of costal valva apodemes. Uncus simple, truncated distally, rectangular. Gnathos well-sclerotized, forming mesally lobed plate below uncus, gnathos spanning tegumen. Valvae small relative to tegumen + uncus, valvae rounded, hardly extending beyond vinculum-tegumen juncture. Saccular edge of valvae with heavily sclerotized knobs near valval base. Diaphragm with dense setae extending outward above phallus. Juxta fused to phallus, encircling it, lateral margins of juxta extend one quarter length of phallus as triangle directed toward apex of phallus, ventral lip of juxta connects to vinculum. Base of phallus weakly sclerotized with undefined coecum phallus. Phallus cylindrical, downwardly angled distally, vesica spiculate. **Female.** Unknown

###### Remarks.

We describe *Fatellalla* for *F.fatella*, an Amazonian species widely distributed in Ecuador, the Brazilian Amazon, and French Guiana. This species is exceptionally rare in collections, with less than ten specimens known to us in global collections. Although St Laurent et al. (2018a) did not sample *F.fatella*, this species clearly belongs to Lacosominae: Alheitini due to the presence of the fused gnathos “plate” which is also observed in alheitine genera *Thaelia*, *Tarema*, and *Alheita*. The genitalia of *Fatellalla* are most similar to those of *Tarema* and *Alheita* (see St Laurent et al. (2017b)), the presence of elongated valvae apodeme extensions basally not unlike those of *Tarema*, support a close relationship between these genera. Our morphological phylogenetic analyses also support the placement of *Fatellalla* within Alheitini due to close placement with several other alheitine genera, namely *Alheita* (Fig. 1, see also Suppl. materials 5–7). Although *Fatellalla* is nested within a poorly supported *Alheita* clade, we maintain this genus as valid pending molecular phylogenetic analyses due to the extremely divergent uncus shape and external appearance which is inconsistent with any observed *Alheita*. We note also a particularly long branch length (relative to other *Alheita*) in our ML analyses, emphasizing the divergent morphological characters of this odd genus.

Schaus’ (1928) placement of this species in *Druentica* was unjustified and erroneous.

##### Herbinalla

Taxon classificationAnimaliaLepidopteraMimallonidae

St Laurent & Kawahara, 2018

Figs 15 , 52 , 97 , 98

###### Type species.

*Cicinnuscaudina* Schaus, 1905.

###### Diagnosis.

Antemedial/ medial areas strongly contrast against the darker, chestnut brown postmedial/submarginal areas, these two distinct regions of color are divided by a pure white, sinuate postmedial line. *Trogopteramana* Schaus displays a similar coloration scheme but the forewing has a distinct tornal notch and an anterior hindwing notch typical of Trogopterini, which is absent in *Herbinalla*. The particularly acute hindwing anal angle, which is accentuated by darker scales at the tips, is largely unique to this genus.

###### Apomorphies.

(1) Seemingly floating sclerotized plate apparently homologous with plate-like gnathos of Alheitini, covered in fine setae, situated centrally within the diaphragm, plate not connected to remainder of the genitalia by any sclerotization (Fig. 15b); (2) The only alheitine genus with narrow and downwardly curved uncus (Fig. 15a) (this itself is not an apomorphy due to elongated/narrow uncus in other mimallonid genera, however its presence coupled with other typical alheitine traits such as valva shape, are unique to this genus).

##### Tarema

Taxon classificationAnimaliaLepidopteraMimallonidae

Schaus, 1896

Figs 14 , 51 , 99 , 100 ; Suppl. material 3: Plate 3

###### Type species.

*Taremarivara* Schaus, 1896.

###### Diagnosis.

Hoary in appearance due to generous amounts of light gray scales present over the entirety of the wings and body. *Tarema* are similar to *Alheita*, particularly by the small size of the moths of both genera, and the postmedial lunule. However, the ventral surface of *Tarema* is not as plainly maculated due to the presence of postmedial lines and the light gray scales.

###### Apomorphies.

(1) Uniquely shaped, often spiked projections emanating from the costal valva apodeme (Fig. 14a); (2) Robust gnathos plate not affixed to tegumen along its length as in related genera (Fig. 14b; Suppl. material 3: Plate 3, 11: A); (3) Pair of elongated spines on phallus, one shorter spine present dorsally, the other more elongated extending laterally nearly length of phallus (Fig. 14c).

###### Remarks.

St Laurent et al. (2018a) did not include this genus in their phylogeny and instead placed this genus in Lacosominae: Alheitini based on morphology, due to strong similarities with the genus *Alheita* that was included. Our ongoing molecular work, which includes broadened taxon coverage, and *Tarema*, fully supports this placement (St Laurent et al. in prep.). Furthermore, the morphological analyses carried out in the present study place *Tarema* nested within Alheitini, either sister to *Herbinalla* (constrained ML analysis, Fig. 1) or sister to the remainder of Alheitini (unconstrained ML and parsimony analyses, Suppl. materials 5, 7 respectively).

##### Thaelia

Taxon classificationAnimaliaLepidopteraMimallonidae

Herbin, 2016

Figs 16 , 53 , 102 , 103

###### Type species.

*Thaelialinamariae* Herbin, 2016.

###### Diagnosis.

The coloration of *Thaelia* species is variable, but wing patterning is consistent. Externally, *Thaelia* have an apical postmedial line and elongated, falcate forewings, thereby making them appear very distinct from other Alheitini (all other Alheitini genera have preapical postmedial lines and less falcate, shorter, more triangular wings).

###### Apomorphies.

See diagnosis and remarks below.

###### Remarks.

The definition of *Thaelia* here follows St Laurent et al. (2018a), based on phylogenetic placement of *T.linamariae* Herbin (type species of *Thaelia*), *T.subrubiginosa* (Dognin), and *T.anysia* (Schaus) within a robustly supported clade (fig. 1 in St Laurent et al. (2018a)). All *Thaelia* species, *sensu* this work, were examined morphologically, and included in our morphological phylogenetic analyses, and all form a well-supported clade in all analyses (see Fig. 1, but also Suppl. materials 5 and 7). There are three distinct male genitalia patterns within this genus: (1) gnathos present, valva apodeme extensions absent, and cornuti present (*T.linamariae*, *T.beniensis* Herbin); (2) gnathos present, valva apodeme extensions present, cornuti absent (*T.anysia*); 3) gnathos absent, valva apodeme extensions present, cornuti absent (*T.subrubiginosa*, *T.inornata* (Druce)). We do not consider it warranted to further break *Thaelia* into separate genera at this time considering the consistent external morphology among these species. Ongoing molecular phylogenetics which include denser sampling of *Thaelia* further support a single genus concept for the species that we place in *Thaelia*.

#### Lacosomini Dyar, 1893

##### Citralla

Taxon classificationAnimaliaLepidopteraMimallonidae

St Laurent & Kawahara gen. n.

http://zoobank.org/24EE693F-1210-4450-A84B-8E6163C58BC4

Figs 115–117 , 118, 119 , 120

###### Type species.

*Trogopterarumina* Druce, 1894: 355, by present designation.

###### Etymology.

The name for this new genus is derived from *citrus* (Latin) referring to the lemon-yellow coloration of the type species of *rumina*, the only recognized species in the genus. The name is feminine.

###### Diagnosis.

This new genus can be recognized by the following combination of characters: bright yellow coloration with gray and pink shading on the tornal region of the forewing and anal angle of the hindwing. The postmedial line of the forewing is faint, crenulate, and incomplete, existing only apically, as a small splotch halfway across the wing, and along the tornal shading. The hindwing displays similar maculation. Ventrally, the antemedial area of the forewing is shaded gray and pink, making *Citralla* and *Zaphanta* the only Mimallonidae genera with completely shaded antemedial regions of the ventral surface of the forewings. *Citralla*, however, lacks the ventral antemedial line present in *Zaphanta*. The prothoracic tibia has a prominent tuft of pink scales that is seen nowhere else in Mimallonidae. The male genitalia are simple, but unique in the absence of gnathos and transtilla projections, and by the simple triangular uncus and narrow valvae. The phallus is nondescript and largely similar to that of *Lacosoma*. The female genitalia are most similar to the related *Vanenga*, but display narrower papillae anales, ostium bursae, and ductus bursae. In *Vanenga* the confluence of the ostium bursae and ductus bursae is almost as wide as segment VIII, but in *Citralla* this part of the ductus bursae is only about one quarter the width of VIII, compare Figs 57, 119.

###### Apomorphies.

Combination of the following characters: (1) Gnathos reduced to narrow bars below uncus which lack both mesal extensions and subuncus projections typical of related genera (*Lacosoma* and *Vanenga*); (2) Simple, smooth, triangular uncus and narrow valvae (relative to sharply triangular or extremely narrow uncus of *Lacosoma* and *Vanenga*).

###### Description.

**Male.***Head*: Gray-brown, eyes very large, occupying more than two-thirds area of head, bordered posteriorly by dark scales; antenna coloration light tan, antenna bipectinate to tip, distalmost 10–12 pectinations significantly shorter; labial palpus three segmented, but segments difficult to discern due to compact scaling. *Thorax*: Coloration light yellow with scales along posterior prothoracic margin and junction with abdomen very faint pink and gray, ventrum pale gray. *Legs*: Coloration variable, prothoracic leg: femur light purple-gray, tibia yellow with light gray scales before juncture with tarsus, prominent tuft of pink scales present on inner margin of tibia apex, tarsus yellow. Mesothoracic leg: femur and tibia light gray, tarsus yellow with some gray scaling apically. Metathoracic leg: all segments predominantly yellow with some gray scaling at terminus of tibia and apex of tarsus. Tibial spurs elongate, narrow, dorsally covered in scales, ventral surface and tip naked, length roughly half length of first tarsal segment. *Forewing dorsum*: Forewing length: 9–14 mm, avg: 11 mm, wingspan: 19–27 mm, n = 16. Triangular, margin nearly straight. Ground color light yellow. Antemedial faint, pink, irregular, antemedial area may be slightly suffused with pink; preapical postmedial line irregular, incomplete, existing only near tornus, apex, and halfway across length of wing as single splotch. Postmedial line outwardly shaded with gray and pink, particularly along tornus where pink suffusion reaches wing margin. Discal mark present as light gray ovoid splotch. Fringe checkered off-white and orange-brown, slightly crenulate. *Forewing ventrum*: Nearly identical to forewing dorsum, but antemedial line absent and antemedial area completely shaded by pink and gray, discal spot more pronounced. *Hindwing dorsum*: Coloration, patterning as for forewing dorsum, but discal mark faint or absent, antemedial line absent. *Hindwing ventrum*: Following same pattern as forewing ventrum. Frenulum present as single bristle. *Venation*: Typical of Mimallonidae. *Abdomen*: Dorsal coloration as for thorax, but slightly darker. *Genitalia*: (Fig. 118) n = 3. Vinculum ovoid, ventrally inwardly notched. Uncus simple, triangular, ventrally membranous. Gnathos and transtilla absent, but sclerotized bars extend downward from uncus/tegumen junction. Valvae narrow, triangular, simple; base of valvae extend centrally above vinculum base such that valvae cannot be fully spread. Juxta fused to phallus, encircling it, extending dorsally above phallus as flattened process. Phallus cylindrical, basally truncated. **Female.***Head*: As for male, but antennae smaller overall. *Thorax*, *Legs*: As for male. *Forewing dorsum*: Forewing length: 11.5–15.0 mm, avg: 13.8, wingspan: 26–30 mm, n = 7. As for male, but slightly broader overall. *Forewing ventrum*: As for male, but slightly broader overall. *Hindwing dorsum*: As for male, but slightly broader overall. *Hindwing ventrum*: Following same pattern as forewing ventrum. Frenulum as multiple bristles. *Abdomen*: As for male, but more robust. *Genitalia*: (Fig. 119) n = 1. Tergite VIII forms smooth, thickened posteriorly directed arch, mesally with cup-like indentation at dorsal base of papillae anales. VIII weakly sclerotized laterally. Apophyses anteriores thick, truncated distally, roughly half length of apophyses posteriores which are outwardly bent halfway along length. Lamella ante- and postvaginalis poorly preserved, but weakly sclerotized without distinguishing features. Ductus bursae long, narrow, about three times the length of VIII-X, ductus widest at convergence with ostium bursae, but remaining very narrow along remainder of length. Corpus bursae small in length in comparison to elongated ductus bursae and large papillae anales, shape balloon-like. Papillae anales narrow, elongated, ventrally angled such that apical ridges of papillae anales and opening between them situated ventrally in nearly same plane as ostium bursae.

###### Remarks.

*Citralla* is here described for the unique species *C.rumina* comb. n., which is distributed from Guatemala to southeastern Brazil. Further taxonomic investigations into the various populations of *C.rumina* will undoubtedly reveal several cryptic species, as we have observed slight external morphological distinctions in populations in Southeastern Brazil and the Amazon, in comparison with topotypical material from Panama and nearby Costa Rica.

Ongoing molecular phylogenetic work which includes *Citralla* consistently places this genus sister to *Vanenga*, which together form a clade sister to *Lacosoma* (St Laurent in prep.). Morphological analyses are less consistent in placement, with our morphological analyses recovering *Citralla* sister to (unconstrained ML analysis) or nested within *Lacosoma* (constrained ML and MP analyses). Regardless, tribal placement of the new genus is confidently in Lacosomini. The substantial reduction of the gnathos and juxtal configuration are most similar to those of both *Lacosoma* and *Vanenga* than to any other know Mimallonidae genus. But the uncus shape and pink scale tufts on the forelegs are unique to *C.rumina*. The larvae of *C.rumina* (Fig. 120) have been reared and photographed by Janzen and Hallwachs (2017), revealing morphology remarkably similar to various species of *Lacosoma*, two species of which have been reared by the first author (St Laurent and Carvalho 2017b, St Laurent et al. 2017). In particular, the striated appearance of the head and prothoracic shield, as well as the less rugose anal plate are largely consistent with *Lacosoma* as opposed to the more uniform coloration and generally more robust anal shield of other groups such as Mimalloninae and Cicinninae. We therefore, consider these characters as additional information supporting our decision to place *Citralla* within the Lacosominae: Lacosomini.

According to Janzen and Hallwachs (2017), the host plant of *C.rumina* in Costa Rica is *Eugeniasalamensis* Donn. Smith (Myrtaceae). Myrtaceae is a host plant family that is frequently a larval resource for Mimallonidae (St Laurent et al. 2018).

##### Lacosoma

Taxon classificationAnimaliaLepidopteraMimallonidae

Grote, 1864

Figs 20 , 56 , 111 , 112 , 163 , 164–165 ; Suppl. material 3: Plate 1

###### Type species.

*Lacosomachiridota* Grote, 1864.

###### Diagnosis.

*Lacosoma* is one of the most speciose genera of Mimallonidae, and displays a great deal of variation. They can easily be recognized by their genitalia (see Apomorphies below), but are externally more variable. Externally, *Lacosoma* are some of the smallest Mimallonidae in overall size, most species have crenulated wing margins and pink, salmon, and gray coloration. The general shape and size of *Lacosoma* species is more diagnostic than any one other external character. The combination of the general characters of small size, falcate forewings, and crenulated margins allow for the recognition of most species in the genus. Few Central American species display straight wing margins (such as *L.elassa* (Franclemont) and *L.morgani* Herbin), but are of the usual size and coloration for *Lacosoma* in general. The more uniquely colored species display the typical falcate wing shape and crenulate wing margins. Examples of uniquely colored species include those that are all pink: *L.maldera* Schaus; nearly all black: *L.syrinx* (Druce) and *L.briasia* Schaus (Fig. 163); and pink and yellow: *L.valera* Schaus and *L.valeroides* Herbin and C. Mielke.

###### Apomorphies.

(1) Subuncus projections extend from ventral margins of tegumen, extended outward most usually as a simply triangular protrusion (Fig. 20a), but in some Central American species (for example *L.elassa*, *L.morgani*) these projections are more elongated and bent; (2) Gnathos projected ventrally below uncus, connecting to complex dorsal projections of juxta, together forming intricate sclerotizations with variable projections (Fig. 20b; Suppl. material 3: Plate 1, 6: 0, 6: A).

##### Vanenga

Taxon classificationAnimaliaLepidopteraMimallonidae

Schaus, 1928

Figs 21 , 57 , 113 , 114

###### Type species.

*Perophoramera* Dognin, 1924.

###### Diagnosis.

Small mimallonids with short triangular forewings with distinct straight postmedial line, ground coloration pale tan-orange with pink coloration throughout the antemedial and medial areas.

###### Apomorphies.

(1) Uncus as long (roughly length of valva), narrow spine-like in shape, not obviously triangular as in essentially all other mimallonid genera (Fig. 21a); (2) Subuncus projections similar in shape to those of *Lacosoma*, but originating from downward lateral projections of tegumen extending well below costal margin of valva (Fig. 21b), as opposed to extending directly outward from ventral margin of tegumen above or very near costal valva margin as in *Lacosoma* (se Fig. 20a).

### Druenticinae St Laurent & Kawahara, 2018

#### Druenticini St Laurent & Kawahara, 2018

##### Druentica

Taxon classificationAnimaliaLepidopteraMimallonidae

Strand, 1932

Figs 22 , 58 , 121 , 122

###### Type species.

*Cicinnuspartha* Schaus, 1905.

###### Diagnosis.

Most *Druentica* are silvery gray medium sized mimallonids with a distinct, usually straight postmedial line, and a preapical black dot along the costa where the postmedial line angles towards the costa, meeting it. Most species have straight forewing edges, but a few (such as *D.rotundula* (Dognin), *D.muta* (Dognin), *D.mutara* (Schaus), and *D.brosica* (Schaus)) have crenulated wing margins not unlike some *Lacosoma* and *Mimallo*, hence erroneous placement of several species of *Druentica* in these unrelated genera. *Druentica* is another of the larger mimallonid genera, and thus there is more interspecific variation in this genus than in other genera previously treated above. However, male genitalia of *Druentica* are immediately recognizable as such.

###### Apomorphies.

Combination of the following characters: (1) Slightly curved tusk-like arms extending from valva base (Fig. 22a); (2) Paired, mesally separated, elongated gnathos arms with distal flattening extend from immediately below uncus, gnathos arms roughly equal to length of uncus (Fig. 22b).

##### Lepismalla

Taxon classificationAnimaliaLepidopteraMimallonidae

St Laurent & Kawahara gen. n.

http://zoobank.org/17A09A1B-0CEC-4FBA-904C-C826A5136BA8

Figs 131 , 132

###### Type species.

*Cicinnusmontagnaniae* Herbin, 2012: 14, by present designation.

###### Etymology.

The name for this new genus is derived from the small, silvery coloration of the sole *Lepismalla* species, reminiscent of silvery Zygentoma in the genus *Lepisma* Linneaus. The ending –alla/-allo has been commonly applied in Mimallonidae. The genus name is feminine.

###### Diagnosis.

The single species of *Lepismalla*, *L.montagnaniae* comb. n., is recognizable by the almost complete lack of markings (except for faint irregular postmedial lines and heavy black discal marking) on the dorsal surface of the wings, which combined with the small size and falcate wing shape is unique in the family Mimallonidae. Genitalia are robust structures, with prominent gnathos arms that extend distally below the saccular margin of the valvae. In this way the gnathos arms are somewhat similar to those of related genera *Procinnus* Herbin and *Micrallo* St Laurent and C. Mielke, but extend much farther ventrally relative to the valvae. The basal valva arms typical of Druenticini are present, but highly reduced, and are flanked by setae covered regions of the sacculus. This genus lacks the claw-like sternite VIII extensions in the male genitalia which are typical of the related genus *Procinnus*, and has much shorter valvae tusks than either *Procinnus* or *Micrallo*.

###### Apomorphies.

(1) Large gnathos arms extend ventrally below saccular edge of valvae such that valvae appear dorsally to gnathos (Fig. 131I); (2) Base of valvae with tiny tusk-like arms (smaller than in any other Druenticini) (Fig. 131II).

###### Description.

**Male.***Head*: Light brown, eyes very large, occupying more than two-thirds area of head; antenna coloration light tan, antenna entirely bipectinate, distalmost quarter length of antennae with pectinations markedly shorter; labial palpus apparently two segmented, though a third segment may be present but small. *Thorax*: Light brown, lightly speckled with darker petiolate scales. *Legs*: Coloration as for thorax, vestiture thick, long. Tibial spurs elongate. *Forewing dorsum*: Forewing length: 14.5–16.0 mm, avg.: 14.6 mm, wingspan: 28–35 mm, n = 6. Triangular, margin concave mesally forming falcate apex. Ground color silvery gray and light brown, overall lightly flecked with dark brown petiolate scales. Antemedial line absent; preapical postmedial line faint, outwardly convex, consisting of numerous barely distinguishable individual dark brown petiolate scales. Discal mark present as irregular black splotch heavily contrasting against light ground color and otherwise largely unmarked surface. Fringe light brown, lightening to cream near tornus. *Forewing ventrum*: As for forewing dorsum, but more brown than gray, anal region light tan, apex lighter gray than surrounding brown area; postmedial line slightly more well-defined; discal mark fainter than for dorsum. *Hindwing dorsum*: Coloration, patterning as for forewing dorsum, but discal mark much fainter to nearly absent. Fringe nearly white along most of wing margin. *Hindwing ventrum*: Following same pattern as forewing ventrum, but lighter overall, more of a continuation of light tan of anal area of forewing ventrum. Frenulum as single bristle, though difficult to see. *Venation*: Typical of Mimallonidae. *Abdomen*: Dorsal coloration as for thorax, ventrally lighter gray. Vestiture appearing thinner in comparison with thorax. Sternite of VIII with narrow pair of sclerotizations. *Genitalia*: (Fig. 131) n = 2. Vinculum ovoid, ventrally slightly pointed. Uncus simple, broad, triangular, distally narrowed to thin point. Gnathos massive, the most distinct aspect of the genitalia, gnathos heavily sclerotized with paired mesal arms which extend beyond and below saccular edge of valvae, gnathos arms distally and inwardly membranous, small tooth present at apex of each arm, gnathos arms fused mesally at base by narrow sclerotized band. Valvae small relative to gnathos, somewhat rectangular in shape, terminally with slight saccular lobe. Pair of small (length less than one third length of valvae), tusk-like curled sclerotized arms extend outward from saccular edge of valvae, tusk-like arms inwardly flanked by protruding setae covered sacculus. Juxta as thin sclerotization ventral to phallus forming connection with vinculum. Phallus curved when viewed laterally, viewed dorsally/ventrally phallus flattened distally with membranous opening such that phallus appears spade-like with sclerotized ring forming margins of spade shape, basal half of phallus as membranous sack. **Female.** Unknown

###### Remarks.

*Lepismalla* is described for the unique Amazonian taxon *L.montagnaniae*, which is so far outwardly and by male genitalia, unlike any other Mimallonidae, though is most similar to related genera *Procinnus* and *Micrallo*. In the original description, it was suggested that “*Cicinnus*” *montagnaniae* might be more properly placed in a new genus (Herbin 2012). After examining genitalia of all genera in prior to and in preparation of the present work, it is clear the genitalia of *L.montagnaniae* do not conform to any other generic concept of Mimallonidae, and certainly this species is unique externally as well. However, the subfamily and tribal apomorphies provided by St Laurent et al. (2018a) for Druenticini are consistent with the general male genitalia characters seen in *L.montagnaniae*. Our morphological and ongoing molecular phylogenetic analyses also consistently place this taxon as sister to the druenticine genus *Procinnus*, within a robustly supported Druenticini clade (St Laurent et al. in prep.).

The shape of the phallus of *Lepismalla* is very similar to that of other druenticine genera *Micrallo* and *Procinnus*. The phallus in all three genera is flattened with a unsclerotized central region in the distal half, which is clearly visible from the dorsal or ventral aspect. These genera also display a membranous sack-like region along the basal dorsal half of the phallus, though the size of this structure differs between the genera. Paired sclerotizations of the VIII sternite typical of Druenticinae, including *Micrallo* and *Procinnus*, are present in *Lepismalla*, but are reduced to narrow sclerotized strips in the otherwise membranous intersegmental region. The silvery gray coloration of *Lepismalla* is a coloration scheme seen in almost all Druenticini genera (except the darkly colored *Ulaluma* and some *Procinnus*), and the gnathos configuration is not unlike that of *Procinnus* and *Micrallo*, but simply more robust and ventrally farther reaching, such that in *Lepismalla* the gnathos arms actually reach below the saccular edge of the valvae. The basal valvae arms typical of Druenticini are present in *Lepismalla*, albeit greatly reduced.

*Lepismallamontagnaniae* is rare in collections, with only a handful of specimens known to us in global collections. Almost all examined material comes from the Amazon Rainforest, although one specimen, unfortunately destroyed in the fire at the Museu Nacional, Rio de Janeiro, Brazil, was from Mato Grosso do Sul, Brazil, in quite a different habitat on the border of Cerrado and Pantanal. An additional specimen in CPAC is from Distrito Federal in the Brazilian Cerrado. Future, finer scale, examinations of all known *Lepismalla* specimens may eventually reveal that this is not a monotypic genus, though it is certainly not diverse.

##### Micrallo

Taxon classificationAnimaliaLepidopteraMimallonidae

St Laurent & C. Mielke, 2016

Figs 23 , 60 , 123 , 124

###### Type species.

*Micrallominutus* St Laurent & C. Mielke, 2016.

###### Diagnosis.

Tiny to small (forewing length: 11.5–15 mm) silver gray mimallonids, with elongate triangular forewings, and well-defined, straight forewing postmedial lines that are perpendicularly angled toward the costa, immediately separates this genus from others in the family.

###### Apomorphies.

(1) Deeply curled, concave tegumen + vinculum shaped like a broad cesta of Jai alai in the lateral aspect; (2) Basally membranous, distally narrowed, terminally clubbed valvae (Fig. 23a); (3) Membranous pockets encase valvae, contain thick, elongate deciduous setae (Fig. 23b).

###### Remarks.

St Laurent et al. (2018a) did not include this genus in their phylogeny and instead placed this genus in Druenticinae: Druenticini based on morphology, due to strong genitalia similarities with the sampled genus *Procinnus*, particularly the flattened, spade-like phallus, which we now also observe in the newly described *Lepismalla*. All morphological phylogenetic analyses carried out here also place *Micrallo* within Druenticini, either sister to *Ulaluma* (parsimony analysis) or to *Procinnus* + *Lepismalla* (both ML analyses), thus confirming the tribal placement of St Laurent et al. (2018a).

An undescribed species that likely belongs to this genus is larger and browner than *M.minutus*, but otherwise bears the same arrangements of wing markings and genital configuration.

##### Pamea

Taxon classificationAnimaliaLepidopteraMimallonidae

Walker, 1855

Figs 25 , 62 , 125 , 126

###### Type species.

*Pameaalbistriga* Walker, 1855.

###### Diagnosis.

Quite similar to some of the smaller Andean *Druentica* species, but instead consistently have white outlined postmedial lines and white suffusions submarginally which contrast against a pale gray ground color finely stippled with darker petiolate scales. *Pamea* lack the black costal dot of *Druentica*.

###### Apomorphies.

In general, similar to *Druentica* but (1) Gnathos arms smaller, flatter distally, and very weakly sclerotized, almost membranous at their termini (Fig. 25a); (2) Basal valvae arms coiled (forming loop) (Fig. 25b), arms never simply curved as in *Druentica* (e.g., Fig. 22a).

###### Remarks.

St Laurent et al. (2018a) did not include this genus in their phylogeny and instead placed this genus in Druenticinae: Druenticini based on morphology, due to clear similarities to the genus *Druentica* (particularly the gnathos shape), *Druentica* was included by these authors. As was the case with the two previously treated Druenticini genera not formally included in any molecular phylogenetic analyses, the male genitalia morphology of *Pamea* allow confident placement of this genus within Druenticini. Our morphological phylogenetic analyses confirm this placement, consistently placing *Pamea* sister to the remainder of Druenticini (both ML analyses). Our ongoing molecular work which includes *Pamea*, also support this placement (St Laurent et al. in prep.).

##### Procinnus

Taxon classificationAnimaliaLepidopteraMimallonidae

Herbin, 2016

Figs 26 , 59 , 127 , 128

###### Type species.

*Procinnuscahureli* Herbin, 2016.

###### Diagnosis.

Narrow, elongate, sharply acutely triangular wings; coloration varies from silver-gray to brown, but wing shape along with lack of hyaline patches on all wings, and presence of well-defined discal marking unify this small genus.

###### Apomorphy.

(1) Pair of elongated, sharp projections extend from either side of VIII sternite, which together with the basal valva arms typical of Druenticini and low-set gnathos arms, give the lower half of the genitalia an overall appearance of possessing a massive multi-fingered claw (Fig. 26a).

###### Remarks.

We note that one undescribed species is known to the first author to belong to this genus based on clear male genitalia characters, but has significantly stouter wings than other *Procinnus* and a preapical postmedial line. The coloration and discal markings of this undescribed taxon are however, typical of this genus. Also in this and another undescribed *Procinnus* species known to the first author, there are fewer sternite VIII arms, but those that are present are the same shape and size as in other described taxa. This combined with the valvae and gnathos shapes are not unlike those of more typical *Procinnus*.

##### Ulaluma

Taxon classificationAnimaliaLepidopteraMimallonidae

St Laurent & Kawahara, 2018

Figs 24 , 61 , 129 , 130

###### Type species.

*Cicinnusvalva* Schaus, 1905.

###### Diagnosis.

Small (average forewing length = 14.1 mm) blackish purple ground color, with yellow postmedial lines on all wings. The forewing margin is sharply pointed mesally at the tip of CuA1.

###### Apomorphy.

The combined absence of the gnathos and the presence of valvae tusks (Fig. 24).

#### Luramini St Laurent & Kawahara, 2018

##### Lurama

Taxon classificationAnimaliaLepidopteraMimallonidae

Schaus, 1928

Figs 27 , 63 , 133–135

###### Type species.

*Perophorapenia* Dognin, 1919.

###### Diagnosis.

Yellow-brown, or in fresh specimens, more golden tan, with distinctly brown antemedial and postmedial lines, and veins accented by the same brown coloration, all of which strongly contrast against the lighter ground color.

###### Apomorphies.

Combination of the following characters: (1) Rectangular valvae, distally concave along margin (Fig. 27a), (2) Parallel setae-covered lobes projecting from the valva apodeme (Fig. 27b) (considered “transtilla” in St Laurent (2016)); (3) Gnathos absent; (4) Thin, fishhook-shaped phallus (Fig. 27c).

##### Ulmara

Taxon classificationAnimaliaLepidopteraMimallonidae

Schaus, 1928

Figs 28 , 64 , 136 , 137 ; Suppl. material 3: Plate 6

###### Type species.

*Cicinnusrotunda* Dognin, 1916.

###### Diagnosis.

Unique, blueish (even somewhat iridescent in fresh specimens) black mimallonids usually with crenulated wing margins, broad wings; comparatively large antennae with some of the longest pectinations in Mimallonidae (this trait is similar to *Cunicumara*). The toothed postmedial lines are inwardly lined with pale brown which contrasts against the dark ground color.

###### Apomorphies.

Similar to *Lurama*, but the combination of the following characters distinguishes *Ulmara*: (1) gnathos present (Fig. 28a) and (2) sternite VIII with bilobed feet-like sclerotizations covered in elongate setae (Fig. 28b).

### Cicinninae Schaus, 1912

#### Psychocampini St Laurent & Kawahara, 2018

##### Biterolfa

Taxon classificationAnimaliaLepidopteraMimallonidae

Schaus, 1928

Figs 29 , 65 , 138 , 139

###### Type species.

*Cicinnusalthea* Schaus, 1905.

###### Diagnosis.

All three species of *Biterolfa* are similar, primarily differentiated by genitalia characters. Externally *Biterolfa* are brown to red-brown with a dark gray-brown postmedial line outwardly margined with a thin gray submarginal region which narrows from the tornus to the falcate apex. Distinctive, parallel, white outlined dark streaks span across the middle of the forewing towards the costa.

###### Apomorphies.

Combination of following characters: (1) Broad, stout, simple valvae without any spines, protrusions, or arms (Fig. 29a); (2) Juxta with paired knob-like lobes situated laterally on either side of phallus (Fig. 29b); (3) Prominent gnathos, mesally unsclerotized, with pair of robust, thickly sclerotized arms (Fig. 29c); ventrum of arms may be toothed, terminus of gnathos arms distinctly narrowed with finger-like process at tip of each arm, often with secondary dorsal apical protrusion from tip of arm.

###### Remarks.

See remarks of *Psychocampa* below.

##### Psychocampa

Taxon classificationAnimaliaLepidopteraMimallonidae

Grote & Robinson, 1867

Figs 30 , 66 , 140 , 141 , 166

###### Type species.

*Psychocampaconcolor* Grote & Robinson, 1867.

###### Diagnosis.

Externally *Psychocampa* display typical cicinnine shape and large size, and always lack hyaline patches, however it is difficult to generalize about this relatively large genus beyond that. Despite external variability across the genus, *Psychocampa* genitalia are quite homogenous, and therefore species belonging to this genus are easily recognizable by their genitalia, which are dramatically distinct and simple in structure in comparison with all other Cicinninae genera (except for the sister genus *Biterolfa*, see below).

###### Apomorphies.

Largely as for *Biterolfa* but gnathos arms more gradually narrowed, never with teeth or dorsal apical protrusions (in addition to the tapering tip).

###### Remarks.

This is one of the largest Mimallonidae genera in terms of number of species, particularly after the present checklist and the work of St Laurent et al. (2018a) which cumulatively transferred many species from *Cicinnus* to *Psychocampa* based on the exceptionally homogenous and readily diagnostic genitalia of the latter genus. As stated above, it is difficult to diagnose *Psychocampa* based on external morphology because there are several clear species-groups within the genus which may eventually prove to be more appropriately placed in new genera that can more succinctly be unified by external morphology (though genitalia morphology is so consistent across all of these groups that additional atomization of *Psychocampa* into several genera may prove to be unwarranted). Genitalia morphology is largely homogenous across all Psychocampini, not only in *Psychocampa*, and this is clearly evident in comparing the genitalia of sister genera *Psychocampa* and *Biterolfa*, see Figs 29 and 30. However, external maculation and convincing phylogenetic results of St Laurent et al. (2018a) placing *Biterolfa* as sister to the larger clade of *Psychocampa*, which was intentionally sampled to include most of the clear species-groups of the genus, supports their valid separation as two distinct genera. Further ongoing molecular phylogenetics which continues to sample Psychocampini more densely also continues to support the separation of these two genera. Our morphological phylogenetic results here fully support the transfer of various species from *Cicinnus* to *Psychocampa*, as well as the close relationship between *Psychocampa* and *Biterolfa*, though with our increased taxon sampling, the validity of *Biterolfa* is called into question in a morphology-only context because it is nested within *Psychocampa* in unconstrained ML and parsimony morphological phylogenetic analyses. Despite this, we maintain *Biterolfa* and *Psychocampa* as valid separate genera considering the molecular results of St Laurent et al. (2018a) and the previously mentioned ongoing molecular work.

#### Bedosiini St Laurent & Kawahara, 2018

##### Bedosia

Taxon classificationAnimaliaLepidopteraMimallonidae

Schaus, 1928

Figs 31 , 67 , 142 , 143

###### Type species.

*Cicinnusfraterna* Schaus, 1905.

###### Diagnosis.

*Bedosia* is a rather diverse genus in coloration, shape, and size, but species belonging to this genus always have a B-shaped forewing hyaline patch (as is common in Cicinninae) and well defined postmedial lines on all wings. Most species have brown accented veins and triangular wings with straight margins or those that are slightly convex. Male genitalia, however, clearly unify this genus and distinguish *Bedosia* from the externally very similar *Bedosiallo*.

###### Apomorphies.

Combination of the following characters: (1) Valvae situated on dorsal half of vinculum (Fig. 31a) immediately below uncus such that ventral portion of vinculum extends downward as a well-defined projection (Fig. 31d); (2) Heavily concentrated paired set of setae emanate from diaphragm (Fig. 31b); (3) Gnathos arms massive and extremely robust, variable in shape but always arm-like and with species-specific modified tips, extended outward more so than upward (Fig. 31c).

##### Bedosiallo

Taxon classificationAnimaliaLepidopteraMimallonidae

St Laurent & Kawahara, 2018

Figs 32 , 68 , 144 , 145

###### Type species.

*Cicinnusforbesi* Schaus, 1928.

###### Diagnosis.

Much like *Bedosia* in external shape and key features, but usually with much narrower wings; the genitalia, however, are very different from those of *Bedosia*, and are diagnostic of the genus (see apomorphies below).

###### Apomorphies.

Combination of following characters: (1) Small, heavily sclerotized gnathos situated very near uncus with mesal pair of closely parallel arms (distally fused in some species) (Fig. 32a); (2) Simple, narrow widely splayed valvae relative to widened ovoid vinculum (Fig. 32b).

###### Remarks.

*Bedosiallo* was found to be a strongly supported clade in St Laurent et al. (2018a), where two species of this genus that apparently represented two distinct species-groups within *Bedosiallo* were sampled and found sister to one another, which together were sister to *Bedosiafraterna*, type specie *Bedosia*. Although sampling of *Bedosia* was relatively poor in St Laurent et al. (2018a), we have since examined the genitalia of nearly all *Bedosia* species (including those erroneously placed in *Cicinnus* by Schaus (1928)), finding consistent complex male genitalia, which are readily divergent from the much simpler genitalia of *Bedosiallo*. In the description of *Bedosiallo*, all described species were dissected and their genitalia figured, displaying the consistent genitalia in that genus, as well as the consistent difference from all known *Bedosia* species (St Laurent et al. 2018a). Below in the checklist we transfer two additional species to *Bedosiallo* from *Cicinnus*: *B.minimalis* Herbin and C. Mielke, comb. n. and *B.gentilis* (Schaus), comb. n. These two species display genitalia much more in line with those of the described *Bedosiallo* species than to any *Bedosia* species, but do differ in some respects, particularly in the more closely fused gnathos, triangular valvae shape, juxtal configuration, and presence of cornuti (see Annotations in Section 4). *Bedosiallominimalis* was included in our morphological phylogenetic analyses, and was placed within *Bedosiallo* in all analyses (Suppl. materials 5, 7) except the constrained ML analysis (Fig. 1). We attribute the somewhat unique genitalia of *B.minimalis* to result in this conflicting placement, and while *B.gentilis* and *B.minimalis* may eventually prove to belong to an entirely new genus considering such unique traits, we are confident that these small moths are more closely related to *Bedosiallo* than *Bedosia* in comparing the genitalia of all species in Bedosiini, and certainly do not belong in *Cicinnus* as they were originally placed.

#### Cicinnini Schaus, 1912

##### Aceclostria

Taxon classificationAnimaliaLepidopteraMimallonidae

Vuillot, 1893

Figs 33 , 69 , 146 , 147

###### Type species.

*Aceclostriamus* Vuillot, 1893.

###### Diagnosis.

Silvery-gray to gray brown ground color, sinuate, diffuse postmedial lines, falcate forewings, and broad darker gray to dark gray brown discal marks that vary from fully scaled to fully hyaline within species (often from the same series); maculation overall is dark and diffuse giving the wings a soiled appearance.

###### Apomorphy.

Asymmetric, complex genitalia with phallus situated on left side (when viewed ventrally) of vinculum (Fig. 33a) with single vinculum tusk reaching out above phallus.

###### Remarks.

St Laurent et al. (2018a) did not include this genus in their phylogeny and instead placed this genus in Cicinninae: Cicinnini based on morphology, namely the presence of all apomorphies of Cicinninae and Cicinnini in what was at the time the single *Aceclostria* species, *A.mus*. Our ongoing molecular work, which includes broadened taxon coverage, and *Aceclostria*, fully supports this placement (St Laurent et al. in prep.). The morphological phylogenetic analyses carried out here support this placement as well, with *Aceclostria* always recovered within Cicinnini, sister to (unconstrained ML and parsimony analysis, Suppl. materials 5 and 7 respectively) or nested within (constrained ML analysis, Fig. 1) the broader, so far poorly resolved *Cicinnussensu lato* clade.

See annotations in Section 4 for information pertaining to the novel inclusion of *A.cordubensis* comb. n. and *A.nigrescens* comb. n. et stat. rev. in *Aceclostria*. *Aceclostria* was long considered a monotypic genus, but morphological examinations, including the genitalia of *A.cordubensis*, conclusively support these additional poorly known taxa in this genus. This genus is apparently closely associated with Anacardiaceae (see Annotations).

The *Cicinnus* s.l. clade is discussed in more detail below as it is the most poorly resolved set of taxa in the family.

##### Aleyda

Taxon classificationAnimaliaLepidopteraMimallonidae

Schaus, 1928

Figs 34 , 70 , 148 , 149

###### Type species.

*Cicinnusaccipiter* Dognin, 1916.

###### Diagnosis.

Wings are exceptionally narrow and elongate with thin hyaline patch streaks on all wings (hyaline streak mesally kinked on hindwing, lunate on forewing). The antemedial line forms a convex semicircle, whereas the postmedial line is incomplete and mostly limited to the proximal margin of the forewing. The ante- and postmedial lines are curved toward each other or are tangent below discal cell.

###### Apomorphies.

Combination of the following characters: (1) Valva with saccular curl/fold which holds robust vincular tusks (Fig. 34a); (2) Phallus fused with proximally bent juxtal structure below (Fig. 34b).

##### Arcinnus

Taxon classificationAnimaliaLepidopteraMimallonidae

Herbin, 2016

Figs 37 , 150 , 151

###### Type species.

*Arcinnushoedli* Herbin, 2016.

###### Diagnosis.

Externally not particularly distinct from several other species of *Cicinnus*. *Arcinnus* are generally smaller than most *Cicinnus* and always lack hyaline patches on the hindwings (similarly patterned *Cicinnus* have hyaline patches on both fore and hindwings). Genitalia were the primary means by which this genus was originally described, and we also consider the male genitalia to have the most robust diagnostic characters defining this genus.

###### Apomorphies.

(1) Uncus vestigial and not synscleritous with tegumen (Fig. 37a); (2) Most substantial component of genitalia the broad, curved vincular arms which are apparently homologous to those seen in most Cicinninae, albeit much more heavily sclerotized, thicker, and apically blunt (Fig. 37b).

###### Remarks.

St Laurent et al. (2018a) recovered *Arcinnus* as a valid genus sister to *Euphaneta* + *Aleyda*. In the present study, *Arcinnus* is recovered nested within the poorly resolved *Cicinnussensu lato* clade. We hesitate to synonymize this genus at this time pending broader molecular sampling of the *Cicinnus* s.l. clade, which is ongoing and consistently supports the validity of *Arcinnus*.

We were unable to figure female genitalia of *Arcinnus*, but direct the reader to figs 54 and 55 in Herbin (2016), where the female genitalia of the type species, *A.hoedli* are presented.

##### Cicinnus

Taxon classificationAnimaliaLepidopteraMimallonidae

Blanchard, 1852

Figs 36 , 71 , 153 , 154 , 167–168 , 169–171 ; Suppl. material 3: Plate 4

###### Type species.

*Cicinnusorthane* Blanchard, 1852.

###### Diagnosis.

See below in the checklist for specific information pertaining to different species-groups within *Cicinnus*, but in general *Cicinnus* can be recognized by their falcate forewings, moderate to relatively large size compared to most other Mimallonidae genera. Small, B-shaped hyaline patches, when present, are found on all four wings or forewings only. Species without hyaline patches are usually heavily marked with irregular blotches and speckles.

###### Apomorphies.

These apomorphies refer to those of *Cicinnussensu stricto* in St Laurent et al. (2018c) and do not apply to *Cicinnus* s. l.: (1) Vincular arms may (e.g., *C.melsheimeri* (Harris) or may not (e.g., *C.orthane* Blanchard) be present; (2) Valvae mostly membranous, mesally more well-sclerotized, often with distinct “clasper” (Fig. 36a).

###### Remarks.

Annotations following the checklist are provided for each group of *Cicinnus* as we currently define them: *Cicinnus* s. s. (*Cicinnus* Group 1) and *Cicinnus* s. l. (which includes *Cicinnus* s. l. Groups 2, 3, and some species of uncertain placement). The annotations include diagnostic characters which can be treated as apomorphic of these preliminary groupings of similar species, and may eventually be used to define formal genera (e.g., the available name *Gonogramma* Boisduval, see Annotation 98, may eventually be resurrected to include the species treated here as *Cicinnus* s. l.). The morphological phylogenetic analyses do not fully resolve the relationships of species within *Cicinnus*, with relatively low support values for the various groups within the genus. However, *Cicinnus* s. l. is consistently recovered within a broader Cicinnini clade, thus tribal placement of the included species is not in question. Additional molecular sampling of *Cicinnus* and related genera in order to better define the clades within *Cicinnus* s. l. will be a focus of our future molecular work.

##### Euphaneta

Taxon classificationAnimaliaLepidopteraMimallonidae

Schaus, 1928

Figs 35 , 72 , 152

###### Type species.

*Phanetadivisa* Walker, 1855.

###### Diagnosis.

This genus is similar to *Aleyda*, but *Euphaneta* are larger, have broader, more ovoid wings, lunate hyaline patches on all wings, and lack clearly distinct antemedial and postmedial lines, but rather have a dark brown antemedial area which merges with a brown costal region that extends along the length of the wing. *Euphaneta* are cryptically colored, apparently camouflaged resembling a woody stem.

###### Apomorphy.

Hood-like juxtal process (Fig. 35a) dorsal to weakly sclerotized phallus which is shorter than the juxtal hood above it; this juxtal process is a large, substantial structure comprising almost entire diaphragmal area.

###### Remarks.

We were unable to include the female of *E.divisa*, however sexual dimorphism in *Euphaneta* is not overly significant, with females only being larger and with broader wings than the males.

##### Isoscella

Taxon classificationAnimaliaLepidopteraMimallonidae

St Laurent & Carvalho, 2017b

Figs 38 , 73 , 157 , 158 ; Suppl. material 3: Plate 5

###### Type species.

*Perophoraventana* Dognin, 1897.

###### Diagnosis.

*Isoscella* can be recognized by the following combination of characters: narrow, triangular wings with single ovoid (or more circular in some species) discal hyaline patch. The similar genus *Roelmana* always has broader, less elongated wings, and may lack hyaline patches entirely.

###### Apomorphies.

(1) Gnathos rectangular with mesal pair of thin, parallel arms equal in length to lateral bars of gnathos (Fig. 38a); (2) Vincular arms thick, not thin and tusk-like, in most species arms dorsally covered in setae (Fig. 38b); (3) Shorter, secondary pair of setae covered plate-like vincular arms located on either side of phallus (Fig. 38c).

###### Remarks.

In our morphological phylogenetic analyses *Isoscella* and the related *Roelmana* below, were poorly differentiated phylogenetically due to strong morphological similarity between these two genera. However, *Roelmana*, which displays more interspecific variation than within *Isoscella* (see below) was strongly supported as a monophyletic group in St Laurent et al. (2018a), as well as in ongoing molecular phylogenetics that continues to sample more *Roelmana* species. These molecular phylogenetic studies include the several morphologically distinct species-groups of *Roelmana*, which formed a clade sister to *Isoscella*. The most morphologically divergent *Isoscella* species, *I.andina* St Laurent and Carvalho, was not sequenced, but was included in our morphological analyses. This unique species is nested within *Isoscella* in all analyses except the constrained ML analysis (Fig. 1), rendering *Isoscella* paraphyletic in that analysis. We consider the placement of this taxon as sister to all Cicinnini in this analysis anomalous considering the presence of all generic *Isoscella* apomorphies in *I.andina*. The observed placement in Fig. 1 may be due to certain aspects of patterning (e.g., complete, straight ventral forewing postmedial lines in *I.andina*) which are unique to this species, whereas genitalia are typical of *Isoscella*. Therefore, based on the unconstrained analysis, we consider *Isoscella* to represent a monophyletic lineage, whose relationship to species of *Roelmana* will likely be more clearly elucidated with denser taxon sampling in our future molecular work.

##### Roelmana

Taxon classificationAnimaliaLepidopteraMimallonidae

Schaus, 1928

Figs 39 , 74 , 155 , 156 ; Suppl. material 3: Plate 6

###### Type species.

*Cicinnusmaloba* Schaus, 1905.

###### Diagnosis.

Wing shape of *Roelmana* is typical of Cicinnini, but much less variable overall within this genus than in other cicinnine genera. Forewings are sharply falcate, with the anterior margin of the forewing concave, particularly along the tornus. Two distinct wing morphological groups are found in this genus: one that has a hyaline patch on each wing (such as type species *R.maloba* (Schaus) and *R.pluridiscata* (Schaus)), whereas the other group entirely lacks hyaline patches and are instead diffusely colored with faint patterning (such as *R.laguerrei* (Herbin) and *R.beneluzi* (Herbin), comb. n.). However, genitalia are mostly homogenous in this genus and provide clear generic placement for these two otherwise outwardly distinct groups.

###### Apomorphies.

Similar to *Isoscella* but (1) Proximal component of gnathos more heavily sclerotized, more rounded than rectangular, with mesal arms more robust and thicker (Fig. 39a); (2) Primary (the longest) vincular arms sharp and tusk-like, not covered in setae (Fig. 39b).

###### Remarks.

One species, *R.beneluzi*, displays genitalia that are distinct from other *Roelmana*, and our transfer of this species from *Cicinnus* to *Roelmana* is preliminary. See Annotation 126 for further discussion.

### Annotated checklist of Mimallonidae

**Mimallonoidea Burmeister, 1878**

**Mimallonidae Burmeister, 1878**

Perophoridae Plötz, 1885; (Packard 1895), preoccupied

Ptochopsychidae Grote, 1896

Lacosomatidae Brues & Melander, 1915

**Zaphantinae St Laurent & Kawahara, 2018: 739**

***Zaphanta* Dyar, 1910: 85**

***infantilis*** Dyar, 1910: 85* (Guyana, USNM)^1^

***fraterna*** Schaus, 1912: 48, stat. rev. (Costa Rica: Limón, USNM)^2^

**Incertae Sedis**

***Cunicumara* St Laurent, 2016: 86**

***anae*** St Laurent, 2016: 88* (Bolivia: Santa Cruz, CMNH)^3^

***Menevia* Schaus, 1928: 665**

*lantona* species-group

***lantona*** (Schaus, 1905: 327)* (*Cicinnus*) (French Guiana, USNM)

***magna*** St Laurent & Dombroskie, 2016: 49 (Brazil: Santa Catarina, DZUP)

***rosea*** St Laurent & Dombroskie, 2016: 44 (Ecuador: Napo, CMNH)

***torvamessoria*** St Laurent & Dombroskie, 2016: 47 (Peru: Puno, NHMUK)

*lucara* species-group

***lucara*** (Schaus, 1905: 328) (*Cicinnus*) (French Guiana, USNM)

***menapia*** St Laurent & Dombroskie, 2016: 57 (Guatemala: Izabal, USNM)

***mielkei*** St Laurent & Dombroskie, 2016: 59 (Brazil: Minas Gerais, DZUP)

*ostia* species-group

***ostia*** (Druce, 1898: 447) (*Perophora*) (Panama: Chiriquí, MNHU)

***pallida*** Herbin & C. Mielke, 2014: 147 (Brazil: Maranhão, DZUP)

***parostia*** Schaus, 1928: 667 (Unknown, USNM)^4^

*perostia*‡ in Becker (1996), misspelling

*plagiata* species-group: *plagiata* subgroup

***alurca*** Herbin & C. Mielke, 2014: 146 (Brazil: Maranhão, DZUP)

*ulcara*‡ in Herbin & C. Mielke (2014), misspelling

***australis*** St Laurent & Dombroskie, 2016: 86 (Brazil: Santa Catarina, CUIC)

*elegans*‡ Franclemont, unavailable manuscript name^5^

***plagiata*** (Walker, 1855: 1341) (*Mimallo*) (Brazil: Rio de Janeiro, NHMUK)^6^

*superba*‡ Jones, unavailable manuscript name^5^

*plagiata* species-group: *vulgaris* subgroup

***cordillera*** St Laurent & Dombroskie, 2016: 107 (Peru: Puno, NHMUK)

***delphinus*** St Laurent & Dombroskie, 2016: 109 (Brazil: Distrito Federal, CPAC)

***franclemonti*** St Laurent & Dombroskie, 2016: 97 (Brazil: Santa Catarina, CUIC)

*falco*‡ Franclemont, unavailable manuscript name^5^

***vulgaris*** St Laurent & Dombroskie, 2016: 89 (French Guiana, CUIC)

***vulgaricula*** St Laurent & Dombroskie, 2016: 103 (Brazil: Amazonas, CMNH)

***Roelofa* Schaus, 1928: 640**

***elyanae*** Herbin & C. Mielke, 2014: 142 (Brazil: Maranhão, DZUP)

***hegewischi*** (Druce, 1887: 227) (*Perophora*) (Mexico, MNHU)^7^

*hegewishi*‡ Schaus, 1928: 640, misspelling

***maricia*** Schaus, 1928: 640 (Brazil: Rio de Janeiro, ZSM)

***narga*** (Schaus, 1905: 329) (*Cicinnus*) (Surinam, USNM)

*maera* (Schaus, 1913: 5) (*Cicinnus*) (Brazil: Santa Catarina, USNM)

***olivia*** (Schaus, 1896b: 52)* (*Perophora*) (Colombia, USNM)

***Tolypida* Schaus, 1928: 663**

***amaryllis*** (Schaus, 1896a: 143)* (*Hydrias*) (Brazil: São Paulo, USNM)^8^

*alboflava* (Dognin, 1917: 17) (*Cicinnus*) (Brazil: Santa Catarina, USNM)

***spitzi*** Pearson, 1984: 463 (Brazil: Goiás, NHMUK)

**Aurorianinae St Laurent & Kawahara, 2018: 741**

***Auroriana* St Laurent & C. Mielke, 2016: 123**

***colombiana*** St Laurent & C. Mielke, 2016: 127* (Colombia: Meta, NHMUK)

***florianensis*** (Herbin, 2012: 23) (*Cicinnus*) (French Guiana, MNHN)

***gemma*** St Laurent & C. Mielke, 2016: 134 (Brazil: Santa Catarina, DZUP)

**Mimalloninae Burmeister, 1878**

***Eadmuna* Schaus, 1928: 663**

***esperans*** (Schaus, 1905: 327)* (*Cicinnus*) (Brazil: Espírito Santo, USNM)

***guianensis*** St Laurent & Dombroskie, 2015: 59 (French Guiana, CMNH)

***paloa*** Schaus, 1933: 487 (Brazil: São Paulo, USNM)^9^

***pulverula*** (Schaus, 1896b: 52) (*Perophora*) (Brazil: São Paulo, USNM)^10^

***Macessoga* Schaus, 1928: 664**

***aelfrida*** Schaus, 1928: 664 (Brazil: Minas Gerais, ZSM)

***fabia*** (Druce, 1887: 227)* (*Perophora*) (Panama, MNHN)

***hoppia*** Schaus, 1928: 665, stat. n. (Brazil: Rio de Janeiro, MNHU)^11^

***hyginia*** Schaus, 1928: 665, stat. n. (Brazil: Minas Gerais, ZSM)^11^

***laxa*** (Dognin, 1912: 173) (*Cicinnus*) (Argentina: Misiones, USNM)^12^

*lucero*‡ in Piñas (2004), *nomen nudum*^13^

***Mimallo* Hübner, 1820: 190**

*Mamillo*‡ in Weyenbergh (1874), misspelling

***almeidai*** Pearson, 1951: 325 (Brazil: Rio de Janeiro, CEIOC)

***amilia*** (Cramer, 1780: 130)* (*Bombyx*) (Surinam, NHMUK)

*vorax* (Sepp, [1832]: 47) (*Bombyx*) (Surinam, Unknown)

*aemilia*‡ in Herrich-Schäffer (1865), misspelling

***grisea*** (Schaus, 1896b: 52), comb. n. (*Perophora*) (Brazil: Paraná, USNM)^14^

***hector*** Dognin, 1924: 30 (Brazil: Santa Catarina, USNM)

***neoamilia*** Pearson, 1951: 322 (Brazil: Rio de Janeiro, CEIOC)

*saturata* Walker, 1855: 1340, *nomen dubium* (Brazil: Rio de Janeiro, Unknown)^15^

***Tostallo* St Laurent & C. Mielke, 2016: 119**

***albescens*** (Jones, 1912: 435)* (*Perophora*) (Brazil: São Paulo, NHMUK)

**Lacosominae Dyar, 1893**

**Trogopterini St Laurent & Kawahara, 2018: 745**

***Reinmara* Schaus, 1928: 654**

***andensis*** St Laurent, Herbin & C. Mielke, 2017c: 110 (Bolivia: “N. Yungas”, MNHN)

***atlantica*** St Laurent, Herbin & C. Mielke, 2017c: 108 (Brazil: Espírito Santo, DZUP)

***enthona*** (Schaus, 1905: 325)* (*Cicinnus*) (French Guiana, USNM)

***ignea*** St Laurent, Herbin & C. Mielke, 2017c: 123 (Brazil: Santa Catarina, DZUP)

***minasa*** Schaus, 1928: 655 (Brazil: Minas Gerais, MNHU)

***occidentalis*** St Laurent, Herbin & C. Mielke, 2017c: 112 (Ecuador: El Oro, MWM)^16^

***wolfei*** Herbin & C. Mielke, 2014: 144 (Brazil: Maranhão, DZUP)

***Trogoptera* Herrich-Schäffer, [1856]: 60**

*Tomoptera*‡ in Burmeister, 1878, misspelling

***althora*** Schaus, 1928: 652 (Guatemala: Izabal, USNM)^17^

***belilia*** Schaus, 1928: 653 (Brazil: Pará, USNM)

***callinica*** Schaus, 1928: 653 (Brazil: Rio Grande do Sul, MNHU)

***dietricha*** Schaus, 1934: 94 (Brazil: Rio de Janeiro, USNM)

***erosa*** Herrich-Schäffer, [1856]: 60* (Brazil: Rio de Janeiro, Unknown)^18^

***excavata*** (Walker, 1855: 1154) (*Pamea*) (Brazil, probably Rio de Janeiro, HEC)^18^

***guianaca*** Schaus, 1928: 652 (French Guiana, USNM)^19^

***jonica*** Schaus, 1928: 653, stat. n. (Paraguay: Guairá, USNM)^20^

***mahala*** Schaus, 1928: 652, stat. n. (Ecuador: Loja, USNM)^21^

***mana*** Schaus, 1928: 654 (French Guiana, CMNH)

***maroniensis*** Dyar, 1910: 86 (*Pamea*) (French Guiana, USNM)

*maroniensis*‡ Dognin, 1910: 39 (*Pamea*) (French Guiana, USNM)^22^, preoccupied

***micalha*** Schaus, 1928: 653, stat. n. (Mexico: Veracruz, USNM)^23^

***noaha*** Schaus, 1928: 652, stat. n. (Mexico: Veracruz, USNM)^24^

***notata*** (Walker, 1855: 1155) (*Pamea*) (Brazil, probably Rio de Janeiro, HEC)^25^

***salvita*** Schaus, 1928: 653 (Brazil: São Paulo, MNHU)^26^

***sao*** Druce, 1894: 355 (Costa Rica: Limón, NHMUK)

***semililacea*** (Dognin, 1916: 33) (*Pamea*) (French Guiana, USNM)

***tirzaha*** Schaus, 1928: 653 (Panama: Chiriquí, USNM)

**Alheitini St Laurent & Kawahara, 2018: 743**

***Adalgisa* Schaus, 1928: 670**

***croesa*** Schaus, 1928: 671* (Brazil: Santa Catarina, USNM)^27^

***stellifera*** Schaus, 1928: 671 (Paraguay: “Molinas”, USNM)

***Alheita* Schaus, 1928: 668**

***adelitae*** Herbin, 2016: 202 (French Guiana, MNHN)

***anoca*** (Schaus, 1905: 327)* (*Cicinnus*) (French Guiana, USNM)

***beroalda*** Schaus, 1928: 668 (Guatemala: Izabal, USNM)

***counamama*** Herbin, 2015: 86 (French Guiana, MNHN)

***cymbelina*** Schaus, 1928: 668 (Brazil: Pará, USNM)

***hermieri*** Herbin, 2015: 85 (French Guiana, MNHN)

***obscura*** Herbin, 2016: 200 (French Guiana, MNHN)

***pulla*** (Dognin, 1912: 173) (*Cicinnus*) (Colombia, USNM)

***pulloides*** (Dognin, 1921: 19) (*Perophora*) (Colombia: Cundinamarca, USNM)^28^

***rionica*** Schaus, 1928: 669 (Brazil: Amazonas, USNM)

***subnotata*** (Dognin, 1921: 18) (*Perophora*) (Peru: Mariscal Ramón Castilla, USNM)^29^

***Arianula* Herbin, 2012: 25**

***feiranovensis*** Herbin & C. Mielke, 2014: 139 (Brazil: Maranhão, DZUP)

***haxairei*** Herbin, 2012: 27* (French Guiana, MNHN)

***Fatellalla* St Laurent & Kawahara, gen. n.** ^30^

***fatella*** (Schaus, 1905: 326)*, comb. n. (*Cicinnus*) (French Guiana, USNM)

***Herbinalla* St Laurent & Kawahara, 2018: 754^31^**

***caudina*** (Schaus, 1905: 326)* (*Cicinnus*) (French Guiana, USNM)

***Tarema* Schaus, 1896b: 55**

***bruna*** St Laurent, Herbin, & C. Mielke, 2017b: 134 (Brazil: São Paulo, NHMUK)

***fuscosa*** Jones, 1908: 173 (Brazil: Paraná, NHMUK)

***rivara*** Schaus, 1896b: 55* (Brazil: São Paulo, USNM)

*macarina* Schaus, 1928: 670 (Brazil: São Paulo, USNM)^32^

***Thaelia* Herbin, 2016: 178**

***anysia*** (Schaus, 1928: 651) (*Cicinnus*) (French Guiana, CMNH)^33^

***beniensis*** Herbin, 2016: 182 (Bolivia: Beni, MNHN)

***inornata*** (Druce, 1905: 90), comb. n. (*Bombyx*) (Peru: Puno, NHMUK)^34^

***linamariae*** Herbin, 2016: 179* (French Guiana, MNHN)

***subrubiginosa*** (Dognin, 1916: 19) (*Cicinnus*) (Colombia: Cundinamarca, USNM)^33^

**Lacosomini Dyar, 1893**

***Citralla* St Laurent & Kawahara, gen. n.** ^35^

***rumina*** (Druce, 1894: 355)*, comb. n. (*Trogoptera*) (Panama: Chiriquí, NHMUK)

***Lacosoma* Grote, 1864: 77**

*Naniteta* Franclemont, 1973: 10^36^

***arantium*** Herbin, 2016: 191 (French Guiana, MNHN)

***arizonicum*** Dyar, 1898: 44 (USA: Arizona, USNM)

***asea*** Schaus, 1928: 662 (Colombia: Cundinamarca, USNM)

***aurora*** Dognin, 1916: 17 (Colombia: Boyacá, USNM)

***bigodia*** Schaus, 1928: 662 (Brazil: Amazonas, USNM)

***briasia*** Schaus, 1928: 659 (Colombia: Quindío, USNM)^37^

***canens*** Herbin, 2016: 198 (French Guiana, MNHN)

***cantia*** Schaus, 1928: 661 (Brazil: Amazonas, USNM)

***chiridota*** Grote, 1864: 78* (USA: Pennsylvania, ANSP)

***diederica*** Schaus, 1928: 660 (Bolivia: La Paz, USNM)^38^

***elassa*** (Franclemont, 1973: 11) (*Naniteta*) (USA: Texas, USNM)^36^

***horii*** St Laurent & C. Mielke, 2018: 20 (Brazil: Paraná, DZUP)

***julietta*** Dyar, 1913: 316 (Mexico: Veracruz, USNM)

***ladema*** Dognin, 1920: 11 (Colombia: Boyacá, USNM)

***ludolpha*** Schaus, 1928: 660 (Venezuela: Carabobo, USNM)

*streckeri*‡, unavailable manuscript name^39^

***lygia*** (Schaus, 1912: 56) (*Cicinnus*) (Costa Rica: Limón, USNM)

***maldera*** Schaus, 1934: 94 (Brazil: Rio de Janeiro, USNM)

***medalla*** Dyar, 1913: 316 (Mexico: Morelos, USNM)

***miradorensis*** Herbin & Monzón, 2015: 178 (Guatemala: Petén, UVGC)

***morgani*** Herbin & Monzón, 2015: 180 (Guatemala: Petén, UVGC)^40^

***ostrinum*** Herbin, 2016: 196 (French Guiana, MNHN)

***otalla*** Schaus, 1905: 330 (French Guiana, USNM)

***oyapoca*** Schaus, 1928: 662 (French Guiana, USNM)

***perplexa*** Schaus, 1920: 151 (Guatemala: Izabal, USNM)

***philastria*** Schaus, 1928: 660 (Brazil: Amazonas, USNM)

***puniceum*** Herbin, 2016: 193 (French Guiana, MNHN)

***raydela*** Schaus, 1928: 660 (Guatemala: Izabal, USNM)^41^

***rosea*** (Dognin, 1905: 120) (*Perophora*) (Colombia: Cauca, USNM)

***schausi*** Dognin, 1923: 17 (French Guiana, USNM) (replacement name)^42^

*rosea*‡ Schaus, 1905: 328, preoccupied

***subrufescens*** (Dognin, 1916: 32) (*Cicinnus*) (Colombia: Tolima, USNM)^43^

***syrinx*** (Druce, 1898: 447) (*Mimallo*) (Panama: Chiriquí, MNHU)

***turnina*** Schaus, 1928: 660 (Brazil: Amazonas, USNM)

***ursmara*** (Schaus, 1928: 656), comb. n. (*Druentia*) (Bolivia: Cochabamba, USNM)^44^

***valera*** Schaus, 1928: 662 (Venezuela: Trujillo, USNM)

***valeroides*** Herbin & C. Mielke, 2014: 145 (Brazil: Maranhão, DZUP)

***violacea*** (Sepp, [1848]: 67), (*Bombyx*) (Surinam, Unknown)^45^

***vulfreda*** Schaus, 1928: 659 (Colombia: Chocó, MNHU)

***zonoma*** Schaus, 1928: 662 (Mexico: Veracruz, USNM)

***Vanenga* Schaus, 1928: 664**

***mediorosea*** St Laurent & Herbin, 2017: 95 (Brazil: Santa Catarina, CUIC)^46^

*flavirosa*‡ Jones, unavailable manuscript name

*meroides*‡/*meroidea*‡ Schaus, unavailable manuscript name

*roseatincta*‡ Schaus, unavailable manuscript name

***mera*** (Dognin, 1924: 31)* (*Perophora*) (Brazil: Pará, USNM)

**Druenticinae St Laurent & Kawahara, 2018: 745**

**Druenticini St Laurent & Kawahara, 2018: 746**

***Druentica* Strand, 1932: 145 (replacement name)^47^**

*Druentia*‡ Schaus, 1928: 655, preoccupied

***alsa*** (Schaus, 1910: 422) (*Cicinnus*) (Costa Rica: Cartago, USNM)

***brosica*** (Schaus, 1928: 636) (*Mimallo*) (French Guiana, USNM)^48^

***caquetensis*** (Schaus, 1928: 655) (*Druentia*) (Colombia: Caquetá, MNHU)

***coralie*** Herbin, 2016: 167 (French Guiana, MNHN)

***corana*** (Schaus, 1928: 657) (*Druentia*) (Colombia: Quindío, MNHU)

***derrica*** (Schaus, 1928: 657) (*Druentia*) (Peru: Puno, USNM)

***differenciata*** (Bryk, 1953: 185), comb. n. et stat. n. (*Cicinnus*) (Peru: San Martín,

NHRS)^49^

***fanoveira*** Herbin & C. Mielke, 2014: 141 (Brazil: Maranhão, MNHN)

***garretti*** (Schaus, 1934: 92), comb. n. (*Mimallo*) (Brazil: Rio de Janeiro, USNM)^50^

***imperita*** (Dognin, 1905: 120) (*Perophora*) (Peru: Puno, USNM)

***inscita*** (Schaus, 1890: 46) (*Perophora*) (Mexico: Veracruz, USNM)

***inscitoides*** (Dognin, 1923: 24), stat. rev. (*Perophora*) (Brazil: Amazonas, USNM)^51^

***lola*** (Schaus, 1905: 328), comb. n. (*Cicinnus*) (French Guiana, USNM)^52^

***macallia*** (Schaus, 1928: 655) (*Druentia*) (Colombia: Putomayo, MNHU)

***melastoma*** Herbin, 2016: 171 (French Guiana, MNHN)

***muta*** (Dognin, 1912: 172) (*Cicinnus*) (French Guiana, USNM)

***mutara*** (Schaus, 1933: 486) (*Druentia*) (Brazil: Rio de Janeiro, USNM)

***narita*** (Dognin, 1912: 172) (*Cicinnus*) (Colombia: Valle del Cauca, USNM)

***putidula*** (Dognin, 1912: 171), comb. n. (*Cicinnus*) (Colombia: Valle del Cauca, USNM)^53^

***partha*** (Schaus, 1905: 325)* (*Cicinnus*) (French Guiana, USNM)

***patawa*** Herbin, 2016: 169 (French Guiana, MNHN)

***rotundula*** (Dognin, 1916: 21) (*Cicinnus*) (Colombia: Tolima, USNM)

***scissa*** (Herrich-Schäffer, [1856]: 60) (*Euclea*) (Brazil, NHMUK?)

***zikana*** (Schaus, 1928: 655) (*Druentia*) (Brazil: Rio de Janeiro, MNHU)

***Lepismalla* St Laurent & Kawahara, gen. n.^54^**

***montagnaniae*** (Herbin, 2012: 14)*, comb. n. (*Cicinnus*) (French Guiana, MNHN)

***Micrallo* St Laurent & C. Mielke, 2016: 136^55^**

***minutus*** St Laurent & C. Mielke, 2016: 139* (Brazil: Piauí, DZUP)

***Pamea* Walker, 1855: 1153^56^**

*Pomea*‡ in Burmeister, 1878, misspelling

***albistriga*** Walker, 1855: 1154* (Brazil, probably Rio de Janeiro, NHMUK)

***dotta*** Schaus, 1928: 667 (Brazil: Paraná, USNM)

***nana*** (Herrich-Schäffer, [1856]: 60) (*Euclea*) (Brazil: Rio de Janeiro, Unknown)

***Procinnus* Herbin, 2016: 175**

***acuta*** (Schaus, 1892: 327) (*Perophora*) (Brazil: Rio de Janeiro, USNM)

***cahureli*** Herbin, 2016: 176* (French Guiana, MNHN)

***plana*** (Walker, 1855: 1338) (*Mimallo*) (Brazil, Unknown, probably lost)

*diagonalis* (Herrich-Schäffer, [1856]: 60) (*Euclea*) (Brazil, NHMUK)

***producta*** (Dognin, 1901: 176), comb. n. (*Perophora*) (Colombia: Cauca, USNM)^57^

***Ulaluma* St Laurent & Kawahara, 2018: 755**

***valva*** (Schaus, 1905: 329)* (*Cicinnus*) (French Guiana, USNM)

**Luramini St Laurent & Kawahara, 2018: 748**

***Lurama* Schaus, 1928: 667**

*Luramana* Strand, 1932: 147 (unnecessary replacement name)

***penia*** (Dognin, 1919: 6)* (*Perophora*) (Colombia: Distrito Capital, USNM)^58^

***quindiuna*** Schaus, 1928: 668 (Colombia: Quindío, MNHU)^59^

***Ulmara* Schaus, 1928: 666**

***azurula*** St Laurent, 2016: 83 (Peru: Huánuco, AMNH)^60^

***conjuncta*** St Laurent, 2016: 79 (Ecuador: Loja, CMNH)

***dombroskiei*** St Laurent, 2016: 84 (Peru: Puno, NHMUK)

***rotunda*** (Dognin, 1916: 20)* (*Cicinnus*) (Colombia: Tolima, MNHU)

**Cicinninae Schaus, 1912**

**Psychocampini St Laurent & Kawahara, 2018: 753**

***Biterolfa* Schaus, 1928: 666**

***althea*** (Schaus, 1905: 326)* (*Cicinnus*) (French Guiana, USNM)

***rana*** St Laurent, Giusti, & Herbin, 2017: 89 (Ecuador: Napo, MGCL)

*yupanqui*‡ in Piñas (2004), *nomen nudum*^61^

***tinalandia*** St Laurent, Giusti & Herbin, 2017: 93 (Ecuador: Pichincha, MGCL)

***Psychocampa* Grote & Robinson, 1867: 374^62^**

***alcuna*** (Dognin, 1918: 26), stat. rev. (*Cicinnus*) (French Guiana, USNM)^63^

***bactriana*** (Butler, 1878: 77), comb. n. (*Perophora*) (Brazil: Amazonas/Pará, NHMUK)^64^

***batesii*** (Newman, 1854: 5), comb. n. (*Perophora*) (Brazil: Pará, NHMUK, HEC)^65^

***bilinea*** (Schaus, 1904: 141), comb. n. (*Perophora*) (Brazil: Paraná, USNM)^66^

***callipius*** (Schaus, 1928: 650), comb. n. (*Cicinnus*) (French Guiana, MNHU)^67^

***camarinus*** (Schaus, 1928: 648), comb. n. (*Cicinnus*) (Peru: Loreto, MNHU)^68^

***candacus*** (Schaus, 1928: 649), comb. n. (*Cicinnus*) (French Guiana, USNM)^67^

***concolor*** Grote & Robinson, 1867: 375* (Brazil: Pará, Unknown)

*cunona* (Schaus, 1905: 330) (*Cicinnus*) (French Guiana, USNM)

*stenia* (Dyar, 1910: 86) (*Cicinnus*) (Brazil: Pará, USNM)

***funebris*** (Schaus, 1896b: 51), comb. n. (*Perophora*) (Brazil: Paraná, USNM)^69^

***gaujoni*** (Dognin, 1922: 26), comb. n. (*Perophora*) (Ecuador: Loja, USNM)^67^

***hamata*** (Walker, 1855: 975) (*Perophora*) (Brazil: Rio de Janeiro, NHMUK)^70^

*jaruga* (Jones, 1912: 436), syn. n. (*Perophora*) (Brazil: São Paulo, NHMUK)^71^

***joanna*** (Schaus, 1905: 321) (*Cicinnus*) (French Guiana, USNM)^72^

***kohlli*** Herbin, 2012: 9 (French Guiana, MNHN)

***lacuna*** (Schaus, 1910: 418), stat. rev. (*Cicinnus*) (Costa Rica: Cartago, USNM)^73^

***madenus*** (Schaus, 1928: 648), comb. n. (*Cicinnus*) (Brazil: Amazonas, CUIC)^74^

***magnapuncta*** (Kaye, 1901: 157), comb. n. (*Perophora*) (Trinidad, NHMUK)^75^

***manalca*** (Schaus, 1928: 648), comb. n. (*Cicinnus*) (French Guiana, CMNH)^76^

***marona*** (Schaus, 1905: 323) (*Cicinnus*) (French Guiana, USNM)^77^

***mulatro*** (Schaus, 1920: 151), comb. n. (*Cicinnus*) (Guatemala: Izabal, USNM)^78^

***narseres*** (Schaus, 1928: 647), comb. n. (*Cicinnus*) (Bolivia: La Paz, USNM)^67^

***unalca*** (Schaus, 1905: 325) (*Cicinnus*) (Guyana, USNM)^79^

***undiscata*** (Dognin, 1923: 33), stat. rev. (*Perophora*) (Brazil: São Paulo, USNM)^80^

***viemanda*** (Schaus, 1928: 648), comb. n. (*Cicinnus*) (Brazil: Amazonas, USNM)^81^*melini* (Bryk, 1953: 185), syn. n. (*Cicinnus*) (Peru: Loreto, NHRS)^82^

**Bedosiini St Laurent & Kawahara, 2018: 752**

***Bedosia* Schaus, 1928: 657**

***balca*** (Schaus, 1905: 323) (*Cicinnus*) (Guyana, USNM)

***dulcis*** (Schaus, 1910: 420), comb. n. (*Cicinnus*) (Costa Rica: Cartago, USNM)^83^

***euthymius*** (Schaus, 1928: 649), comb. n. (*Cicinnus*) (Colombia: Chocó, MNHU)^84^

***fraterna*** (Schaus, 1905: 330)* (*Cicinnus*) (French Guiana, USNM)

***gilia*** (Schaus, 1905: 322) (*Cicinnus*) (French Guiana, USNM)

***itamaraty*** (Foetterle, 1902: 641) (*Mimallo*) (Brazil: Rio de Janeiro, NMW)^85^

***ligina*** (Schaus, 1910: 419) (*Cicinnus*) (Costa Rica: Cartago, USNM)

***olasis*** (Schaus, 1928: 645), comb. n. (*Cicinnus*) (“Central America”, USNM)^86^

***strigifera*** (C. & R. Felder, 1874: 8) (*Perophora*) (Brazil, NHMUK)^87^

***trailii*** (Butler, 1878: 77) (*Perophora*) (Brazil: Amazonas, NHMUK)

*venata* (Dognin, 1916: 31) (*Cicinnus*) (French Guiana, USNM)

***turgida*** (Schaus, 1910: 420) (*Cicinnus*) (Costa Rica: Limón, USNM)

***yenuga*** Herbin, 2016: 188 (French Guiana, MNHN)

***Bedosiallo* St Laurent & Kawahara, 2018: 756**

*forbesi* species-group

***eugenia*** (Schaus, 1905: 324) (*Cicinnus*) (French Guiana, USNM)

***forbesi*** (Schaus, 1928: 644)* (*Cicinnus*) (French Guiana, USNM)^88^

***sylvia*** (Schaus, 1920: 152) (*Cicinnus*) (Guatemala: Izabal, USNM)

***volucris*** (Schaus, 1910: 421) (*Cicinnus*) (Costa Rica: Cartago, USNM)

*gentilis* species-group^89^

***gentilis*** (Schaus, 1910: 419), comb. n. (*Cicinnus*) (Costa Rica: Cartago, USNM)

***minimalis*** (Herbin & C. Mielke, 2014: 137), comb. n. (*Cicinnus*) (Brazil: Maranhão, DZUP)

*moengus* species-group

***moengus*** (Schaus, 1928: 649) (*Cicinnus*) (Surinam, CUIC)

***styx*** (Herbin, 2012: 6) (*Psychocampa*) (French Guiana, MNHN)

**Cicinnini Schaus, 1912**

***Aceclostria* Vuillot, 1893: CLXXXII**

*Aceclostria*‡ Schaus, 1928: 670, preoccupied

***cordubensis*** (Berg, 1882: 279), comb. n. (*Mimallo*) (Argentina: Córdoba, Unknown)^90^

*schulzii* (Weyenbergh, 1882: 141) (*Mimallo*) (Argentina: Córdoba, MNHU)

***mus*** Vuillot, 1893: CLXXXII* (Brazil: São Paulo, Unknown)

*callinica*‡ Schaus, unavailable manuscript name^91^

*deprava* (Schaus, 1896b: 52) (*Perophora*) (Brazil: São Paulo, USNM)

*villaricensis* (Schaus, 1933: 487) (*Eadmuna*) (Paraguay: Guairá, USNM)^92^

***nigrescens*** (Schaus, 1896b: 51), stat. rev. et comb. n. (*Perophora*) (Brazil: Paraná, USNM)^93^

***Aleyda* Schaus, 1928: 641**

***accipiter*** (Dognin, 1916: 20)* (*Cicinnus*) (Panama: Chiriquí, USNM)

***heppneri*** St Laurent, McCabe, & Malm, 2018: 162 (Peru: San Martín, MGCL)^94^

***Arcinnus* Herbin, 2016: 183**

***hoedli*** Herbin, 2016: 185* (French Guiana, MNHN)

***xingua*** (Dognin, 1923: 23), comb. n. (*Perophora*) (Brazil: Pará, USNM)^95^

***Cicinnus* Blanchard, 1852: 66^96^**

*Saccophora*‡ Harris in Doubleday, 1841: 101, preoccupied

*Perophora*‡ Harris, 1841: 299, preoccupied

*Gonogramma* Boisduval, 1872: 96^97^

*Ptochopsyche* Grote, 1896: [4] (replacement name for *Perophora* Harris)

Group 1 (*Cicinnussensu stricto*)^98^

***chaubaudi*** Dyar, 1914b: 391 (Mexico: Estado de México, USNM)

***incerta*** (Möschler, 1878: 676) (*Mimallo*) (Surinam, MNHU)^99^

***latris*** Schaus, 1910: 418 (Costa Rica: Cartago, USNM)^100^

***melgibsoni*** Herbin & Monzón, 2015: 184 (Guatemala: Petén, UVGC)

***melsheimeri*** (Harris in Doubleday, 1841: 101) (*Saccophora*) (USA: Pennsylvania, MCZ)

*melsheimerii* (Harris, 1841: 299) (*Perophora*) (USA: Pennsylvania, MCZ)^101^

*egenaria* (Walker, 1866: 1575) (*Arhodia*) (No locality given, but probably USA, NHMUK)^102^

*primolus* Schaus, 1928: 647 (No locality given, but probably USA, MNHU)^102^

***mexicana*** (Druce, 1898: 446) (*Perophora*) (Mexico: Veracruz, USNM)

***orthane*** Blanchard, 1852: 66* (Chile: Concepción [probably erroneous, likely Brazil], NHMUK)^103^

*orthana*‡ Berg, 1876: 170, misspelling

***pudens*** Schaus, 1911: 191 (Costa Rica: Cartago, USNM)

***solvens*** Dyar, 1914a: 453 (Panama: Panama, USNM)^104^

***tuisana*** Schaus, 1910: 417 (Costa Rica: Cartago, USNM)^105^

Group 2 (*Cicinnussensu lato*)^106^

***bahamensis*** St Laurent & McCabe, 2016: 561 (Bahamas: Great Exuma, CUIC)

***belaria*** (Schaus, 1928: 637), comb. n. (*Psychocampa*) (Brazil: Minas Gerais, MNHU)^107^

***cerradensis*** Herbin & C. Mielke, 2014: 133 (Brazil: Maranhão, DZUP)

***conlani*** Herbin & C. Mielke, 2014: 135 (Brazil: Maranhão, DZUP)

***corallina*** Dognin, 1918: 27 (Colombia: Cundinamarca, USNM)^108^

***corcovada*** (Schaus, 1892: 326) (*Perophora*) (Brazil: Rio de Janeiro, USNM)

***despecta*** (Walker, 1855) (*Mimallo*) (Brazil: Rio de Janeiro, NHMUK)^109^

*curtisea* (Weyenbergh, 1874: 220) (*Mimallo*) (Argentina, probably Buenos Aires, Unknown)^109, 110^

*sanguinolenta* (C. & R. Felder, 1874: 8) (*Perophora*) (Brazil, NHMUK)^109, 111^

*sachinius* Schaus, 1934: 93 (Brazil: Minas Gerais, USNM)^109, 112^

***eminens*** (Dognin, 1923: 16) (*Perophora*) (Colombia: Distrito Capitol, USNM)^113^

***externa*** (Moore, 1882: 358) (*Perophora*) (Brazil: São Paulo, Unknown)

*roscida* Dognin, 1910: 38, syn. rev. (Brazil: São Paulo, USNM)^114^

***falcoargenteus*** St Laurent & McCabe, 2016: 566 (Venezuela: Carabobo, CUIC)

***felderia*** Schaus, 1928: 643 (Mexico: Guerrero, USNM)

***floris*** St Laurent, 2017: 23 (Panama: Panamá [Taboga Island], NHMUK)

***fogia*** Schaus, 1905: 321 (French Guiana, USNM)

***giustii*** St Laurent, 2017: 25 (Colombia: Valle del Cauca, USNM)

***hanseni*** Herbin & Monzón, 2015: 182 (Guatemala: Petén, UVGC)

***malca*** Schaus, 1905: 322 (French Guiana, USNM)

***manicora*** (Schaus, 1928: 637), comb. n. (*Psychocampa*) (Brazil: Amazonas, USNM)^115^

***maranhensis*** Herbin & C. Mielke, 2014: 132 (Brazil: Maranhão, DZUP)

***motagus*** Schaus, 1920: 151 (Guatemala: Izabal, USNM)

***musa*** (Schaus, 1896b: 51) (*Perophora*) (Brazil: Paraná, USNM)

***packardii*** (Grote, 1865: 251) (*Perophora*) (Cuba, ANSP)

*packardi*‡ misspelling, various authors e.g., Schaus (1928)

***patawaensis*** Herbin, 2012: 11 (French Guiana, MNHN)

***submarcata*** Schaus, 1905: 320 (French Guiana, USNM)

***thermesia*** (Jones, 1921: 353) (*Perophora*) (Brazil: São Paulo, NHMUK)^116^

Group 3 (*Cicinnussensu lato*)^117^

***beta*** Schaus, 1910: 421 (Costa Rica: Cartago, USNM)^118^

***gaia*** Herbin, 2015: 84 (French Guiana, MNHN)^119^

***kawensis*** Herbin, 2012: 16 (French Guiana, MNHN)

***mamorensis*** Herbin, 2012: 21 (Bolivia: Beni, MNHN)

***trini*** St Laurent & Cock, 2017: 55 (Trinidad, USNM)

***veigli*** (Schaus, 1934: 92) (*Psychocampa*) (Brazil: Minas Gerais, USNM)

Group undetermined (*Cicinnussensu lato*)

***bibula*** (Dognin, 1924: 31), comb. n. (*Perophora*) (Brazil: Pará, USNM)^120^

***haromana*** Herbin & C. Mielke, 2014: 134 (Brazil: Maranhão, DZUP)

***lemoulti*** Schaus, 1905: 329, comb. rev. (*Cicinnus*) (French Guiana, USNM)^121^

***mawaja*** (Dognin, 1922: 27), comb. n. (*Perophora*) (Brazil: Pará, USNM)^122^

***Euphaneta* Schaus, 1928: 637 (replacement name)**

*Phaneta*‡ Walker, 1855: 1382, preoccupied

***divisa*** (Walker, 1855: 1383)* (*Phaneta*) (Brazil: Pará, NHMUK)

***romani*** Bryk, 1953: 186 (Peru: Ucayali, NHRS) ^123^

***Isoscella* St Laurent & Carvalho, 2017b: 93**

***andina*** St Laurent & Carvalho, 2017b: 106 (Peru: Junín, MGCL)

***ecuadoriana*** St Laurent & Carvalho, 2017b: 97 (Ecuador: Napo, MWM)

***leva*** St Laurent & Carvalho, 2017b: 101 (Peru: Puno, NHMUK)

***peigleri*** St Laurent & Carvalho, 2017b: 104 (Ecuador: Carchi, MWM)

***ventana*** (Dognin, 1897: 243)* (*Perophora*) (Venezuela: Mérida, USNM)^124^

***Roelmana* Schaus, 1928: 671^125^**

***beneluzi*** (Herbin, 2012: 19), comb. n. (*Cicinnus*) (French Guiana, MNHN)^126^

***brasiliensis*** (Herbin & C. Mielke, 2014: 138), comb. n. (*Cicinnus*) (Brazil: Maranhão, DZUP)^127^

***doralica*** (Schaus, 1928: 637), comb. n. (*Psychocampa*) (Colombia: Boyacá, USNM)^128^

***fenestrata*** (Jones, 1912: 436), comb. n. (*Perophora*) (Brazil: Paraná, NHMUK)^127^

***laguerrei*** (Herbin, 2012: 2) (*Psychocampa*) (French Guiana, MNHN)

***maloba*** (Schaus, 1905: 324)* (*Cicinnus*) (French Guiana, USNM)

***pluridiscata*** (Dognin, 1916: 18) (*Cicinnus*) (Peru: Puno, USNM)

***prominens*** (Schaus, 1910: 417), comb. n. (*Cicinnus*) (Costa Rica: Cartago, USNM)^129^

***vitreata*** (Schaus, 1905: 324), comb. n. (*Cicinnus*) (French Guiana, USNM)^128^

## Annotations

1 The type locality of *Z.infantilis* has remained unclear since its original description. Dyar (1910: 85) mentioned four male specimens from two locations (Rockstone, Guyana and St. Jean, French Guiana), but did not say which locality corresponded to the type that he did explicitly reference (“*Type*: No, 13065, U.S. National Museum”). We were able to locate this type from Rockstone, Guyana and consider it to be the holotype. Two additional specimens are curated with the holotype in the type collection of the USNM matching the other locality (St. Jean), and are therefore considered paratypes.

2 As part of an ongoing revision of *Zaphanta*, which includes dissections of all *Zaphanta* species and populations, it is apparent that the Central American *Z.fraterna* stat. rev. is markedly distinct from the Amazonian *Z.infantilis* (St Laurent and Giusti in prep.). We therefore revalidate *Z.fraterna* in the present checklist.

3 The monotypic *Cunicumara* was considered “*incertae sedis*” along with *Menevia, Roelofa*, and *Tolypida* by St Laurent et al. (2018a), due to the fact that these genera did not form a monophyletic group, despite displaying similar genitalia structures in each genus. Our morphological phylogenetic results also support the close association between *Cunicumara* and *incertae sedis* genera, as stated above in the remarks of that genus treatment.

4 Unfortunately, the type locality of *M.parostia* is unknown. It has been suggested that *M.pallida* may be a synonym of *M.parostia* (St Laurent and Dombroskie 2016) due to their similar appearance (small size, maculation). However, the genitalia of the type of *M.parostia* have not been compared to those of female *M.pallida*, thus the validity of *M.pallida* remains uncertain, especially considering that the type locality of *M.parostia* will likely never be known.

5 The taxonomy of *M.plagiata* and related species was confusing until the revision of *Menevia* by St Laurent and Dombroskie (2016). These authors explained the existence of several manuscript names and pseudotypes. Franclemont had labeled several pseudotypes of *M.australis* and *M.franclemonti* with the manuscript names *M.elegans*‡ and *M.falco*‡ respectively, which are both unavailable. These specimens are in the CUIC. Jones also labeled a pseudotype of *M.plagiata* with the unavailable manuscript name *Perophorasuperba*‡ in the NHMUK.

6 The original primary type of *M.plagiata* is lost, along with many types described by Walker originating from the Fry collection (Becker 2001). St Laurent and Dombroskie (2016) designated a neotype for this species, thereby stabilizing the uncertainty of the name.

7 Becker (1996) incorrectly listed Veracruz, Mexico as the type locality for *R.hegewischi*, when in fact the primary type in ZSM only has written on data labels “Mexico”. In Druce’s (1887) original description, he stated that a specific locality within Mexico was not known “but most likely came from the southern part of that country” (Druce 1887: 227).

8 Confusion has surrounded the type locality and original combination of *Tolypidaamaryllis*, the type species of *Tolypida*. *Motadaamaryllis* Schaus, 1896a: 142 and *Hydriasamaryllis* Schaus, 1896a: 143, both bearing the specific name *amaryllis*, were described by Schaus in the same publication, and both had the type locality of Castro, Paraná, Brazil (Schaus 1896a). *Motadaamaryllis* is a synonym of the Arctiinae*Paraclesbrunnea* (Hübner), whereas *Hydriasamaryllis* is a mimallonid and was transferred to *Tolypida* by Schaus (1928). Problems with the incorrect original combination can be traced back to Fletcher and Nye (1982) who first listed *Motada* as the original combination for *T.amaryllis* when listing the type species of the mimallonid genus *Tolypida*. This incorrect original combination was repeated by Becker (1996). Furthermore, an apparently incorrect type locality (Castro, Paraná, Brazil) was given by Schaus (1896a) in the original description of *Hydriasamaryllis*, which was repeated by Becker (1996). The name-bearing type specimen in the USNM is labeled as being from São Paulo. The inconsistency in the reported type locality between Schaus’ original description and the apparent type specimen in the USNM was first noted by Pearson (1984) in his revision of *Tolypida*. Pearson (1984) mentioned that the type locality given by Schaus (1896a) did not match what was written on the labels of the type specimen, and also pointed out that wing measurements reported by Schaus do not match original description. Therefore, we designate a lectotype for the specimen in the USNM from São Paulo (Fig. 159) as it is the only known *Tolypidaamaryllis* specimen labeled as “type” in Schaus’ handwriting, and we correct the type locality of *T.amaryllis* to be São Paulo. This specimen bears the following labels: Sao Paulo S.E. Brazil./ Type No. 11431 U.S.N.M/ Collection Wm Schaus/ USNM-Mimal: 1127/ *Hydriasamaryllis* Type. Schaus/. A red handwritten label reading “LECTOTYPE♂ *Hydriasamaryllis* Schaus, des. by St Laurent” has been added to this specimen.

9 *Eadmunapaloa* was treated by Becker (1996) as a synonym of *E.esperans* without justification. St Laurent and Dombroskie (2015) showed that these two sympatric species display distinct morphological differences warranting their treatment as separate species. The type locality listed in Becker (1996) (Paraguay), is incorrect.

10 *Eadmunapulverula* was transferred from *Cicinnus* to *Eadmuna* by St Laurent and Dombroskie (2015) based on external and genital morphology. This species is known only from a single female specimen, though a second specimen has since been discovered in the NHMUK. However, this other specimen is lacking an abdomen so it will be impossible to know for certain its identity since the diagnostic characters of this rare species are in the genitalia and a ventral abdominal stripe.

11 *Macessogahoppia* stat. n. and *M.hyginia* stat. n. were both originally described as “forms” of *M.aelfrida* by Schaus (1928). However, an ongoing revision of *Macessoga* (St Laurent and Herbin, in prep.) has determined that these “forms” will likely prove to be valid species. Article 45.6.4 permits such a status change, from a “form” to a species (ICZN 1999).

12 *Macessogalaxa* was placed in *Druentica* by Schaus (1928), without justification. St Laurent et al. (2018a) transferred this species to *Macessoga* based on molecular and morphological grounds. Our morphological analyses support this finding.

13 As mentioned in St Laurent et al. (2017c), all names proposed by Piñas in “Mariposas del Ecuador” (Piñas Rubio 2004, 2007) are unavailable *nomina nuda* as they do not satisfy Article 13.1.1 of the ICZN (no written description given) (ICZN 1999). Therefore, we officially designate the name *lucero*‡ Piñas Rubio as *nomen nudum*. In fact, the taxon figured in Piñas Rubio as “*lucero*” is undescribed, and belongs to a likewise undescribed genus that is known to R. St Laurent. This genus is most similar to *Macessoga*, and thus we include the *nomen nudum* with *Macessoga* in our list for organizational purposes.

14 *Mimallogrisea* comb. n. was placed in *Trogoptera* since Schaus (1928), without justification. Examination of the male genitalia of *M.grisea* reveals consistency with all other known *Mimallo* species (see Suppl. material 3: Plate 3). Specifically, *Mimallo* species share the presence of narrow, twisted valva apodeme projections. Furthermore, *M.grisea* has a forewing hyaline patch, which is found in all *Mimallo* species and is absent in *Trogoptera*. The morphological phylogenetic analyses consistently recover *M.grisea* nested within *Mimallo*. Ongoing molecular phylogenetics also support placement of *M.grisea* in *Mimallo*.

15 *Mimallosaturata*‡ was treated as *nomen dubium* by St Laurent and Dombroskie (2016) due to the likelihood that the type is lost, and the fact that Walker’s (1855) description matched no known mimallonid. We confirm that *M.saturata* remains an unknown taxon and should be maintained as *nomen dubium*.

16 St Laurent et al. (2017c) speculated that “*Psychocampanocturna*‡ Piñas [Rubio]” figured in Piñas Rubio (2007) could be *Reinmaraoccidentalis*. Subsequent to the revision of *Reinmara* (St Laurent et al. 2017c), St Laurent became aware of the publication of Piñas Rubio (2004), in which the collecting data for “*Psychocampanocturna*‡” is given as: “Ecuador, Napo, San Rafael, 1550 m” leading us to believe that the specimen figured in Piñas Rubio (2004) is not likely *R.occidentalis*, a species that seems to be restricted to low elevations of the western slopes of the Andes. *Reinmaraoccidentalis* also lacks a forewing tornal notch, which is present but reduced in the specimen figured in Piñas (2004). As mentioned in St Laurent et al. (2017c), all names proposed by Piñas Rubio in “Mariposas del Ecuador” are unavailable *nomina nuda* as they do not satisfy Article 13.1.1 of the ICZN (no written description given) (ICZN 1999).

17 *Trogopteraalthora* was described from an indeterminate number of specimens from Guatemala and Costa Rica. Considering that this may lead to some confusion in identifying “true” *T.althora*, we designate a lectotype for the specimen labeled as the “type” in the USNM (Fig. 160). This specimen bears the following labels: Cayuga Guat/ May/ Schaus and Barnes coll./ Type No. 33569 U.S.N.M/ USNM-Mimal: 1048/ *Trogopteraalthora* Type Schaus/. A red handwritten label reading “LECTOTYPE♂ *Trogopteraalthora* Schaus, des. by St Laurent” has been added to the specimen.

18 The identity of *Trogopteraerosa* has not been clear due to the lack of any type material, besides the figure in Herrich-Schäffer, [1856]. *Trogopteraerosa* is darker, with more sharply angled forewing apices than in the other common southeastern Brazilian species *Trogopteranotata*/*excavata* which may or may not prove to be synonymous names since the type of *T.notata* is male and that of *T.excavata* is female.

19 St Laurent and Cock (2017) designated a lectotype for *T.guianaca* from French Guiana because Schaus (1928) described this species from syntypes from different localities, likely including multiple taxa. *Trogopteraguianaca* was originally described as a subspecies of *T.notata* but was later treated as a valid species by Becker (1996) without any indication of its new status. We maintain *T.guianaca* as a valid species.

20 *Trogopterajonica* stat. n. was originally treated as a subspecies of *T.althora* by Schaus (1928). However, externally *T.jonica* is unlike *T.althora*, particularly in terms of wing shape and maculation. *Trogopterajonica* is more similar to species from Brazil than to Central American species such as *T.salvita*. Schaus (1928) drew this comparison in the description of *T.salvita*, despite naming *T.jonica* as a subspecies of Central American *T.althora* in the same work. *Trogopterajonica* is mostly known from Paraguay, Bolivia and Brazil, and is very similar to the Brazilian *T.salvita* which displays straighter wing margins in the female and darker maculation in both sexes. A substantial series of specimens from Paraguay, Bolivia, and Mato Grosso, Brazil (*T.jonica*) and Goiás, Brazil (*T.salvita*) in the NHMUK, and comparison to the types of both species revealed that the identities of these two species are often confused in collections. To further complicate the matter, the type of *T.jonica* is male, whereas that of *T.salvita* is female. The aforementioned series in the NHMUK, which contains both sexes of both species, allowed us to determine their distinction as two separate species.

21 *Trogopteramahlaha* stat. n. was originally treated as a “form” of *T.althora* by Schaus (1928). However, *T.mahlaha* is undoubtedly a distinct species based on external morphology and distribution (*T.mahlaha* is from Ecuador, whereas *T.althora* was described from Guatemala).

22 *Trogopteramaroniensis* (Dyar) is a senior homonym of *T.maroniensis* (Dognin). Schaus (1928), as the first reviser, treated Dyar’s name as the valid name to apply to this Amazonian *Trogoptera* species. Slight maculation differences (the straightness of the forewing postmedial line) between the respective types of *T.maroniensis* (Dyar) and *T.maroniensis* (Dognin) can likely be attributed to individual variation as examination of several other specimens from French Guiana likewise revealed variation in the same character. In order to clarify their synonymy, genitalia should be examined of both types in a formal revision of *Trogoptera*.

23 *Trogopteramicalha* stat. n. was originally treated as a subspecies of *T.althora* by Schaus (1928). The wing shape of *T.micalha* is broader, and more rounded than either *T.althora* or *T.noaha* (also described from Veracruz, Mexico). This taxon likely should be treated as a valid species, and we propose this classification pending a comprehensive revision of *Trogoptera*.

24 *Trogopteranoaha* stat. n. was originally treated as a “form” of *T.althora* by Schaus (1928). Externally, *T.noaha* is similar in appearance to *T.althora*, but displays reduced maculation on all wings. Rather than maintain the name *noaha* as a “form”, we elevate this taxon to species pending a comprehensive revision of *Trogoptera*.

25 *Trogopteranotata* is a problematic species because its holotype specimen lacks specific locality data within Brazil, though which was probably Rio de Janeiro since most of the Walker-era Mimallonidae were collected in that Brazilian state. Schaus (1896c) originally thought that *T.notata* and *T.excavata* represented different sexes of the same species, though in his revision of Mimallonidae, he treated them as distinct species (Schaus 1928). We believe that *T.notata* and *T.excavata* may be conspecific, but pending a more thorough revision of *Trogoptera*, we treat them as distinct as did Schaus (1928).

26 *Trogopterasalvita* has been often confused with *T.jonica*. However, the two species appear to be distinct, with the wing margins of female *T.salvita* being more convex. *Trogopterasalvita* is found in the Brazilian Cerrado, whereas *T.jonica* is so far known from Paraguay and neighboring Argentina, Brazil, and Bolivia. The type locality given by Schaus (1928) “Santa Catarina” is the incorrect Brazilian state; the type locality of *T.salvita*, which is Casa Branca, Brazil, is an old location in western/northern São Paulo state (C. Mielke pers. comm.). Becker (1996) was seemingly uncertain about this locality, listing it as “SC/SP”. This distinction is important because the type locality of *T.salvita* at Casa Branca is within the Cerrado biome, to which this species is endemic. This information allowed direct comparisons to several specimens in the NHMUK, which were also collected in the Cerrado (Goiás, Brazil), thereby allowing the recognition of this species, and its distinction from *T.jonica*.

27 *Adalgisacroesa* was described from an indeterminate number of specimens from Santa Catarina, Brazil, and French Guiana. Considering the likelihood that *Adalgisa* from these two localities may eventually prove to be distinct species, and in order to stabilize the identity of *A.croesa*, we designate a lectotype for the specimen labeled as the “type” in the USNM. This specimen (Fig. 161) bears the following labels: 695 Blumenau, X Sta. Cath. [illegible]/ spec. fig/ Type No. 33597 U.S.N.M/ USNM-Mimal: 1125/ *Adalgisacroesa* Type Schaus/. A red handwritten label reading “LECTOTYPE♂ *Adalgisacroesa* Schaus, designated by St Laurent” has been added to the specimen. The type locality is therefore Blumenau, Santa Catarina, Brazil.

28 *Alheitapulloides* was described from four male specimens from Guatemala, Colombia, French Guiana, and Peru. Although Dognin (1921) listed the type being from Medina, Colombia, the presence of possible syntypes from distant localities encouraged us to designate a lectotype for the specimen labeled as the “type” from Medina, Colombia in the USNM. The lectotype specimen (Fig. 162) bears the following labels: Medina, Ost Colomb. Coll. Fassl/ Dognin Collection/ Type No. 29692 U.S.N.M/ USNM-Mimal: 1115/ *Perophorapulloides* Type♂ Dognin./. A red handwritten label reading “LECTOTYPE♂ *Perophorapulloides* Dognin, designated by St Laurent” has been added to the specimen.

29 Becker (1996) incorrectly listed the type locality of *A.subnotata* as “Brazil (AM)”. The correct locality is Pebas, Peru.

30 We describe a new genus, *Fatellalla* gen. n., for *F.fatella* comb. n. Although this genus was not part of the phylogenetic analyses of St Laurent et al. (2018a), *Fatellalla* is unambiguously part of Lacosominae: Alheitini based on male genitalia characteristics and our morphological phylogenetic analyses. See genus description above for diagnosis, apomorphies, description, and figures for this genus.

31 The monotypic genus *Herbinalla* was described by St Laurent and Kawahara in St Laurent et al. (2018a) to include the enigmatic species *H.caudina* based on both phylogenomic and morphological analyses, this species was incorrectly placed in *Alheita* by Schaus (1928).

32 *Taremamacarina* was treated as synonym of *T.rivara* by St Laurent et al. (2017b), who noted that the type of *T.macarina* was the sexually dimorphic female of *T.rivara*.

33 *Thaeliasubrubiginosa* and *T.anysia* were erroneously placed in *Alheita* and *Cicinnus* (respectively) by Schaus. Both species were transferred to *Thaelia* by St Laurent et al. (2018a) based on their phylogenetic results. External morphology also supports the new combinations of these two species. However, genitalia of *T.subrubiginosa* and *T.anysia* are rather distinct from the type species of *Thaelia* figured in Herbin (2016).

34 *Thaeliainornata* comb. n. was omitted from Becker (1996). Although *T.inornata* was not included in St Laurent et al. (2018a), examination of genitalia and external morphology of *T.inornata* suggests it is morphologically very similar to *T.subrubiginosa*, and we therefore transfer it to *Thaelia*. The morphological phylogenetic analyses consistently recover *T.inornata* nested within *Thaelia*.

35 We describe a new genus, *Citralla* gen. n., for *C.rumina* comb. n. Based on male genitalia, larval characteristics, and our morphological phylogenetic analyses, we can unambiguously place this new genus in Lacosominae: Lacosomini and ongoing molecular phylogenetics supports the novelty of this new genus (St Laurent in prep.). See genus description above for diagnosis, apomorphies, description, and figures for this genus. The type locality for “*Trogoptera*” *rumina* in Becker (1996) was given as Costa Rica, but this was erroneous as the type specimen and original description clearly indicate “Volcan de Chiriqui,” Panama, as the type locality.

36 *Naniteta* was described by Franclemont (1973) to include *Lacosomaelassa*, described in the same work. The new genus was compared only to the unrelated *Cicinnus* by Franclemont. St Laurent et al. (2018a) showed that another species, *L.morgani*, which displays genitalia morphology that is essentially identical to those of *L.elassa*, and similar external morphology, was nested within *Lacosoma*. *Naniteta* was thereby synonymized by St Laurent et al. (2018a). We can confirm this synonymy here as *L.elassa* was firmly nested within *Lacosoma* in all analyses. It remains uncertain whether *L.elassa* and the similar *L.morgani* are synonymous, as the genitalia and external morphology of the two species are quite similar. *Lacosomaperplexa* and *L.miradorensis* are also similar to these two species, but they are slightly larger, and an examination of the genitalia of a topotypical specimen of *L.perplexa* (St Laurent diss.: 4-16-18:1, USNM) revealed characters distinct from types of *L.miradorensis*, *L.morgani*, and *L.elassa*.

37 *Lacosomabriasia* was described from an indeterminate number of specimens from far reaching localities in Brazil, Colombia, and French Guiana. Considering that this may lead to some confusion in identifying “true” *L.briasia*, we designate a lectotype for the specimen labeled as the “type” in the USNM (Fig. 163). This specimen bears the following labels: Buena Vista Colombia/ Type No. 33579 U.S.N.M/ S.C. Patchett Coll./ USNM-Mimal: 1075/ *Lacosomabriasia* Type Schaus/. A red handwritten label reading “LECTOTYPE♂ *Lacosomabriasia* Schaus, des. by St Laurent” has been added to the specimen.

38 *Lacosomadiederica* was described from an indeterminate number of specimens from Brazil, Bolivia, and Paraguay. Considering that this may lead to some confusion in identifying “true” *L.diederica*, we designate a lectotype for the specimen labeled as the “type” in the USNM (Fig. 164). This specimen bears the following labels: Rio Songo Bolivia 750 m Coll. Fassl/ Dognin Collection/♂ genitalia slide, 6 Mch. ’28 C.H. #5/ Genitalia slide By C.H. #5 USNM 86061/ Type No. 33582 U.S.N.M/ USNM-Mimal: 1079/ *Lacosomadiederica* Type Schaus/. A red handwritten label reading “LECTOTYPE♂ *Lacosomadiederica* Schaus, des. by St Laurent” has been added to the specimen.

39 There is a specimen labeled “Type” in CMNH of “*Lacosomastreckeri*‡” from San Esteban Valley (Carabobo), Venezuela. The specimen appears to be *L.ludolpha* and perhaps for this reason was never formally described, hence the name appears to be an unavailable manuscript name.

40 *Lacosomamorgani* may be a synonym of *L.elassa*; both species are nearly identical in male genitalia and external morphology, but the two species are described from distant localities (Guatemala for *L.morgani*, and southern Texas, USA for *L.elassa*). More material of *L.elassa*, especially from farther south, would be needed to compare directly with *L.morgani*.

41 *Lacosomaraydela* was described from an indeterminate number of specimens from Guatemala and Costa Rica. Considering that this may lead to some confusion in identifying “true” *L.raydela*, we designate a lectotype for the specimen labeled as the “type” in the USNM (Fig. 165). This specimen bears the following labels: Cayuga Guat/ Febr./ Schaus and Barnes coll/♂ genitalia slide, 31 Mch. ’28 C.H. #37/ Genitalia Slide By C.H. #37 USNM 86060/ Type No. 33581 U.S.N.M/ USNM-Mimal: 1078/ *Lacosomaraydela* Type Schaus/. A red handwritten label reading “LECTOTYPE♂ *Lacosomaraydela* Schaus, designated by St Laurent” has been added to the specimen.

42 *Lacosomaschausi* was proposed as a replacement name by Dognin (1923) for *L.rosea*‡ Schaus, 1905 (preoccupied). This is not to be confused with *L.rosea* (Dognin, 1905: 120), a separate species, and the source of the preoccupation of the name “*rosea*”.

43 *Lacosomasubrufescens* was transferred to *Lacosoma* from *Trogoptera* by St Laurent and Mielke (2018) based on genitalia. This species is closely related to *L.asea* and *L.ursmara*. All three of these *Lacosoma* species are Andean, inhabiting moderate to high elevations, and together form a unique morphological group within the genus.

44 *Lacosomaursmara* comb. n. is transferred from *Druentica* based on the discovery of female *L.asea*, which is remarkably similar to the female type of *L.ursmara*. The two species are from far separated type localities (*L.asea* in Colombia, *L.ursmara* in Bolivia), so we do not treat them as synonyms pending an inclusive revision of *Lacosoma*. Both species are Andean endemics and are relatively rare in collections. The morphological phylogenetic analyses consistently recover *L.ursmara* nested within *Lacosoma*.

45 *Lacosomaviolacea* is a taxon of uncertain identity. No specimens have been found attributed to this name, and the only information pertaining to this species are the original figures and text in Sepp [1818]. Externally, the specimen figured in Sepp is unlike any other known Mimallonidae, particularly the blue coloration and arrangement of the postmedial line on the ventrum of the forewings. In fact, the color blue is not known from any Mimallonidae. It is possible that Sepp’s illustration is a misrepresentation of a more characteristically colored mimallonid species, and thus we consider Sepp’s illustration unreliable for accurately assigning the name *violacea*. Forbes (1942) believed that the specimen figured in Sepp is a member of Thyrididae, and Schaus (1928) was uncertain that *L.violacea* should be treated as a *Lacosoma*. However, the larva and feeding behavior illustrated and described in Sepp [1818] unquestionably belongs to Mimallonidae, though it is impossible to ascertain whether the larva and “blue” adult are conspecific.

46 *Vanengamediorosea* was described for the *Vanenga* populations inhabiting the Brazilian Atlantic Forest, the Pampa, Paraguay, and Argentina. This species has had at least three manuscript names variously applied to it in the CUIC, NHMUK, and USNM, but was never formally described until St Laurent and Herbin (2017). Previous authors may have decided it could be conspecific with the much more rarely collected, Amazonian *V.mera*. No names attributed to this species are available except for *V.mediorosea*.

47 *Druentica* was proposed by Strand (1932) as a replacement name for the preoccupied *Druentia* Schaus.

48 *Druenticabrosica* was transferred to *Druentica* from *Mimallo* in St Laurent et al. (2018a) based on molecular and morphological (external and genitalia) data.

49 *Druenticadifferenciata* comb. n. et stat. n. was described by Bryk (1953) as a subspecies of “*Cicinnusputidula*” (=*Druenticaputidula* comb. n., *sensu* this work). By external morphology alone, *D.differenciata* and *D.putidula* are easily distinguishable by the dark gray ground color and contrasting white postmedial line of *D.putidula*, whereas *D.differenciata* is a more typical *Druentica*, externally essentially indistinguishable from several species such as *D.scissa* and *D.melastoma*, among others. There is no doubt that the name *differenciata* should not be associated with *D.putidula* by subspecific status. In fact, the recently described *D.melastoma* may eventually prove to be synonymous with *D.differenciata* because external and genitalia characters of the types of both species are nearly identical (St Laurent pers. obs.). However, considering that *D.melastoma* was described from French Guiana and *D.differenciata* from Peru, we cannot say for certain if they should be synonymized, at least pending a revision of *Druentica*. COI barcodes are available for *D.melastoma*, thus it would be desirable to compare them to topotypical *D.differenciata*. For now, we transfer *D.differenciata* from *Cicinnus* and maintain it as a valid species level taxon for we are at least certain of its generic placement. Our morphological phylogenetic analyses all consistently placed this species in *Druentica*.

50 *Druenticagarretti* comb. n. displays external and genital morphology (MWM genitalia preparation 35.544) similar to *D.brosica*, and the former is unlike any described *Mimallo*. Pearson (1951), in his revision of *Mimallo*, recognized that *D.garretti* and *D.brosica* did not belong in *Mimallo*, but did not transfer these species out of *Mimallo*, or suggest a better genus in which to place them. All morphological phylogenetic analyses consistently recover *D.garretti* nested within *Druentica*, thus supporting this new combination.

51 *Druenticainscitoides* stat. rev. was treated as a synonym of *D.scissa* by Schaus (1928). Upon the first author’s examination of both types, we determine that *D.inscitoides* should be maintained as a valid species pending a revision of *Druentica*.

52 *Druenticalola* comb. n. is hereby transferred to *Druentica* from *Lacosoma* based on external and genital morphology. *Druenticalola* is more similar to *D.mutara* than any *Lacosoma* species, though a genitalia dissection (St Laurent diss.: CPC2 in CRAS) of *D.lola* from French Guiana reveals distinct basal valvae arms, valvae, and phallus structure consistent with more typical *Druentica* species. The morphological phylogenetic analyses consistently recover *D.lola* nested within *Druentica*.

53 *Druenticaputidula* comb. n. is hereby transferred to *Druentica* from *Cicinnus* based on external and genital morphology. All morphological phylogenetic analyses consistently recover *D.putidula* nested within *Druentica*.

54 We describe a new genus, *Lepismalla* gen. n., for the unique species *L.montagnaniae* comb. n. Based on male genitalia and our morphological phylogenetic analyses, we can unequivocally place this new genus in Druenticini, refuting placement in *Cicinnus* as the original combination had proposed. See genus description above for diagnosis, apomorphies, description, and figures for this genus. Ongoing molecular phylogenetics which includes *Lepismalla* concurs with the novelty of *Lepismalla* as a valid genus, and its placement in Druenticini (St Laurent in prep.).

55 *Micrallo* was preliminarily placed in Druenticini by St Laurent et al. (2018a) based on morphology: external and genitalia characteristics of both sexes of the type species of *Micrallo* are similar in several respects to those of *Procinnus*. *Micrallo* was one of the genera missing from the molecular phylogeny of St Laurent et al. (2018a). As discussed above in the generic treatment, *Micrallo* is consistently recovered nested within a broader Druenticini clade in all morphological phylogenetic analyses, thus confirming its original placement by St Laurent et al. (2018a).

56 The identity of the three species of *Pamea* is uncertain. We have not been able to locate the type(s) of *P.nana*, but the figures in Herrich-Schäffer (1856) display a male specimen not unlike the male types of *P.albistriga* and *P.dotta*. Pending a revision of *Pamea*, the three species are currently treated as valid. Kirby (1892) considered *P.nana* a synonym of *P.albistriga*, though this synonymy was overlooked by later authors (e.g., Schaus 1928, Becker 1996). Examination of the *Pamea* specimens at the NHMUK reveals several specimens labeled by earlier workers as either *nana* or *albistriga*, supporting the idea that the names are likely synonymous. Other cases do exist of Herrich-Schäffer describing the same species previously (by a matter of a year or two) described by Walker, for example *Procinnusplana* (Walker) and its synonym *P.diagonalis* (Herrich-Schäffer). *Pamea* was not included in St Laurent et al. (2018a), but our morphological phylogenetics and ongoing molecular phylogenomics that both do include *Pamea*, strongly support the placement of this genus in Druenticini.

57 We transfer *Procinnusproducta* comb. n. from *Cicinnus* to *Procinnus* based on external and genitalia morphology. The genitalia of a male were examined by brushing, and are unambiguous, matching very closely the described *Procinnus* species. Due to the rarity of this species we did not fully dissect *P.producta* at this time, and thus did not code it for morphology. The genitalia were readily visible due to brushing, however, and thus we are confident of its generic placement. In the original description, Dognin (1901) recognized the similarity to *P.plana* (as its synonym *P.diagonalis*) The date of publication of the name was incorrectly listed as 1905 in Schaus (1928), Gaede (1931), and Becker (1996).

58 As explained in St Laurent (2016), the genitalia preparation of the type, and only known specimen of *L.penia*, is missing. However, this species is externally distinct from *L.quindiuna* and thus both species were treated as valid, which is followed here.

59 St Laurent (2016) determined that the abdomen attached to the type of *L.quindiuna* originated from an entirely different Lepidoptera specimen. Unfortunately, the lack of genitalia from either type specime of *Lurama* results in some uncertainty as to the identity of the two species in this genus. However, examination of 12 male genitalia preparations from the entire distribution of *L.quindiuna* (with the exception of the type locality), revealed uniform genitalia characteristics despite minor external differences across populations (St Laurent 2016). Attempts should be made to rediscover *L.penia* in nature or in natural history collections. Furthermore, examination of the genitalia, and a study of additional topotypical *L.quindiuna* would be desirable as well.

60 Subsequent to the description of *U.azurula*, an additional specimen from Peru: Junín: 38 km SW of Mazamari, 2540 m (deposited in the MGCL) has been examined. This specimen was collected roughly halfway between the type localities of *U.azurula* and *U.dombroskiei*, and did not show any intermediate characters between these two species. This specimen displays external and male genitalia characteristics typical of *U.azurula*, supporting the validity of this rarely collected species.

61 As mentioned in St Laurent et al. (2017c, d), all names proposed by Piñas Rubio in “Mariposas del Ecuador” are unavailable *nomen nuda* as they do not satisfy Article 13.1.1 of the ICZN (no written description given) (ICZN 1999). In their revision of *Biterolfa*St Laurent et al. (2017d) previously designated the name *yupanqui*‡ as *nomen nudum*.

62 See St Laurent et al. (2018a) for a discussion on the great confusion surrounding taxa placed in the paraphyletic genus *Psychocampa*. These authors transferred four species to *Psychocampa* from *Cicinnus* based on molecular and morphological data. We now transfer the remaining species previously placed in *Cicinnus* to *Psychocampa* following the phylogeny of St Laurent et al. (2018a), exhaustive genitalia examinations of nearly all species, morphological phylogenetics in the present study, and ongoing phylogenomics.

63 *Psychocampaalcuna* stat. rev. was previously treated as synonym of *P.hamata* (Dognin 1923, Schaus 1928, Gaede 1931, Becker 1996). Genitalia and external morphology support the revalidation of *P.alcuna*, but we recommend a formal revision of this species complex. *Psychocampahamata* was described from Rio de Janeiro, Brazil whereas *P.alcuna* was described from French Guiana. Therefore, previous discussions of “*Psychocampahamata*” in St Laurent et al. (2018a) refer to *P.alcuna*. St Laurent et al. (2018a) transferred *P.hamata* (=*P.alcuna*) to *Psychocampa* based on molecular and morphological evidence. While we here recognize the separation of *P.alcuna* and *P.hamata* as distinct species, we also note that these two species are morphologically very similar, being one another’s Amazonian and Brazilian Atlantic Forest counterparts in a species pair as is commonly encountered in Mimallonidae (e.g., *Meneviavulgaris* and *M.franclemonti* in St Laurent and Dombroskie (2016)). Thus, the transfer made in St Laurent et al. (2018a) remains valid and allows the transfer of *alcuna* and *hamata* to *Psychocampa*. Furthermore, since the previous status of the name *alcuna* was as a synonym of “*Cicinnushamata*” in Becker (1996), and *hamata* was transferred to *Psychocampa* by St Laurent et al. (2018a) along with its associated synonyms by default, the present combination of *P.alcuna* is not a new combination, but only a revived status. *Psychocampaalcuna* was consistently recovered within a broader *Psychocampa* + *Biterolfa* clade in all morphological phylogenetic analyses.

64 *Psychocampabactriana* comb. n. belongs to a relatively large group of similar species including *P.marona*, a species included in the phylogenetic study of St Laurent et al. (2018a). *Psychocampamarona* was nested within *Psychocampa* in St Laurent et al. (2018a), and genitalia are nearly identical to those of *P.concolor*, the type species of *Psychocampa*. We therefore transfer *P.bactriana* (and the remaining similar species) to *Psychocampa* from *Cicinnus* based on this molecular and morphological evidence. Genitalia information is based on males that we have examined that correspond to the female holotype of *P.bactriana*. The type of *P.bactriana* was historically dissected, but the preparation has since been lost.

65 *Psychocampabatesii* comb. n. is very similar to *P.bactriana*. Upon direct comparison of the holotype of *P.bactriana* (NHMUK) and several syntypes of *P.batesii* (NHMUK, HEC), *P.batesii* is a decidedly smaller and lighter colored with reduced maculation. The forewing postmedial line in *P.batesii* runs closer to the wing margin than in any other similar species. *Psychocampabatesii* also resembles *P.callipius*, but in the latter species the forewing postmedial line approaches the discal spot and is more distal from the wing margin. An in-depth revision of this complex of species is warranted.

66 Although outwardly similar to *Cicinnus* s. s., a dissection of a male *P.bilinea* comb. n. surprisingly revealed genitalia diagnostic of *Psychocampa*, and therefore we move this species to *Psychocampa* from *Cicinnus*. All of our morphological phylogenetic analyses support this transfer as well.

67 *Psychocampacallipius* comb. n., *P.candacus* comb. n., *P.gaujoni* comb. n., and *P.narseres* comb. n. are hereby transferred to *Psychocampa* from *Cicinnus* based on phylogenetic and morphological data which places *P.marona* within the *Psychocampa* clade *sensu*St Laurent et al. (2018a). These species were not included in the morphological phylogenetic analyses due their close similarity to *P.marona*, which was sampled for molecular data in St Laurent et al. (2018a). Genitalia of all of these species have since been examined subsequent to the morphological phylogenetic analyses in this article, and support their placement in *Psychocampa*.

68 Since the original description of “*Cicinnus*” *camarinus* in Schaus (1928), there has been uncertainty about the identity of this species. Recently, the first author examined the female type specimen in the MNHU. Surprisingly, *P.camarinus* comb. n. is externally extremely similar to *P.alcuna* from French Guiana and *P.hamata* from Rio de Janeiro, Brazil (see Annotations 63 and 70 regarding the separation of *P.alcuna* and *P.hamata*). *Psychocampacamarinus* was described from Iquitos, Loreto, Peru. Many Amazonian mimallonids described from French Guiana are known from Peru (e.g., St Laurent and Dombroskie 2016, St Laurent et al. 2017d), therefore it is possible that *P.camarinus* may actually prove to be a synonym of *P.alcuna*. However, upon examining several specimens of *P.alcuna* from the eastern Amazon and French Guiana (the latter is the type locality of *P.alcuna*), and comparing them to NHMUK specimens from Peru, it is clear that Peruvian material is distinctly larger and displays slightly more elongated wings. Genitalia are also distinct between the two species, in *P.camarinus* (Peru, St Laurent dissection: NHMUK010402320, NHMUK) the phallus is broader and relatively shorter than the more narrowed phallus of *P.alcuna* (French Guiana, St Laurent dissection 17-3-18:9, MGCL). We therefore maintain *P.camarinus* as valid, and merely transfer it to *Psychocampa* based on its close similarity to *P.alcuna* and *P.hamata*. All of our morphological phylogenetic analyses support this new combination as *P.camarinus* was consistently recovered nested within a broader *Psychocampa* + *Biterolfa* clade.

69 *Psychocampafunebris* comb. n. is hereby transferred to *Psychocampa* based on examination of genitalia which revealed characters completely in line with those of *Psychocampasensu*St Laurent et al. (2018a). This species is likely quite closely related to the previously listed species such as *P.callipius*, *P.candacus*, *P.gaujoni*, and *P.narseres*, but displays much darker coloration such that most patterning is obscured. This uniquely colored species is one of the only described *Psychocampa* to display diurnal behavior (St Laurent and Carvalho 2017a, St Laurent and Carvalho, in prep.). All of our morphological phylogenetic analyses support this new combination as *P.funebris* was consistently recovered nested within a broader *Psychocampa* + *Biterolfa* clade.

70 As previously discussed in Annotation 63, *Psychocampahamata* was transferred to *Psychocampa* based on morphology and the molecular results of St Laurent et al. (2018a). However, the specimen used in their study was from French Guiana, and thus actually represented *P.alcuna* (reinstated from synonymy under *P.hamata*). *Psychocampaalcuna* and *P.hamata* are likely to be sister species considering external and genitalia similarities, thus the transfer made in St Laurent et al. (2018a) was correct. The name *P.hamata* is restricted to the species present in Southeastern Brazil and adjacent regions, genitalia are largely consistent between these populations, but the phallus is narrower and flatter in *P.hamata* than *P.alcuna*.

71 Yet another taxonomic issue concerning the *Psychocampahamata/alcuna* complex arises from the name *jaruga*, proposed by Jones (1912). Jones described *Perophorajaruga* syn. n. from an indeterminate number of specimens from Guarujá, Santos, São Paulo, Brazil. A male specimen labeled as the type is present in the NHMUK and its examination by the first author reveals that it is a close morphological match for the other southeastern Brazilian species: *P.hamata*. In his revision of Mimallonidae, Schaus (1928: 649) stated the following in regards to “*Cicinnusjaruga*”: “The species is unknown to me and the description faulty. Owing to the peculiar black tipped white scales, it is evidently close to *hamata* and *camarinus*.” Therefore, as early as Schaus (1928), there was some concern regarding the identity of this species, already attributing the name *jaruga* to at least being a relative of *P.hamata*/ *alcuna*/ *camarinus*. Therefore, it is uncertain as to why Becker (1996) synonymized the name *jaruga* with the quite distinct “*Cicinnus*” *joanna* (=*Psychocampajoannasensu*St Laurent et al. (2018a) and this paper). We therefore newly synonymize *jaruga* with *P.hamata*, as its synonymy with *P.joanna* was apparently erroneous.

72 *Psychocampajoanna* and *P.unalca* were transferred to *Psychocampa* from *Cicinnus* by St Laurent et al. (2018a) on molecular and morphological grounds. The genitalia of these, and related species (e.g., *P.lacuna*, *P.undiscata*, and *P.viemanda*) are typical of *Psychocampa* and are transferred to that genus here.

73 *Psychocampalacuna* stat. rev. was treated as synonym by Schaus (1928), and this synonymy was followed by subsequent authors (Gaede 1931, Becker 1996). St Laurent and Cock (2017) hypothesized that the synonyms of *P.joanna* would likely prove to be valid species. We revalidate *P.lacuna* based on external and genitalia differences between topotypical *P.joanna* (French Guiana, St Laurent diss.: LEP24627 in MGCL) and near topotypocal *P.lacuna* (Costa Rica, Sixaola River, St Laurent diss.: NHMUK010402321 in NHMUK). In the darker colored *P.joanna*, the gnathos arms are much thinner in comparison with the thick and robust gnathos arms of *P.lacuna*. The phallus of *P.joanna* is also thinner from the lateral aspect (wider when viewed dorsally, flatter when viewed anteriorly) than in *P.lacuna*. Furthermore, the two species have clearly disjunct distributions. The type locality of *P.lacuna* is Costa Rica, but the fist author has examined material from as far north as Guatemala, suggesting that this species is likely widely distributed in Central America. *Psychocampajoanna*, on the other hand, is an Amazonian species. Because the previous status of the name *lacuna* was as a synonym of “*Cicinnusjoanna*” in Becker (1996), and *joanna* was transferred to *Psychocampa* by St Laurent et al. (2018a) along with its associated synonyms by default, the present combination of *P.lacuna* is not a new combination, but only a revived status. Schaus described *P.lacuna* from an indeterminate number of syntypes from Costa Rica, though two specimens (a male and a female) in the USNM are labeled “type”, therefore we designate the male specimen (Fig. 166) as the lectotype. This specimen bears the following labels: JuanVinas CR/ June/ Collection Wm Schaus/ *Cicinnuslacuna* Type♂ Schaus/Type No. 16959 U.S.N.M/. A red handwritten label reading “LECTOTYPE♂ *Cicinnuslacuna* Schaus, designated by St Laurent” has been added to the specimen.

74 *Psychocampamadenus* comb. n. possesses the genitalia typical of *Psychocampa* (*sensu*St Laurent et al. 2018a; based on dissection of genitalia NHMUK010402309 in NHMUK), and is hereby transferred to *Psychocampa* from *Cicinnus*. *Psychocampamadenus* is most similar in external morphology to *P.manalca*, which we also transfer to *Psychocampa* on morphological grounds. All of our morphological phylogenetic analyses support this new combination as *P.madenus* was consistently recovered nested within a broader *Psychocampa* + *Biterolfa* clade.

75 *Psychocampamagnapuncta* comb. n. belongs to a complex of similar species in this genus. As per St Laurent and Cock (2017), only females from Trinidad have so far been collected or photographed, and thus there is some uncertainty as to the identity of the male and validity of this species. St Laurent and Cock (2017) designated and figured the lectotype of *P.magnapuncta*. This species was not included in our morphological phylogenetic analyses due to its rarity, and the fact that males remain unknown (and thus could not be coded for male genitalia). External morphology is sufficiently close to other *Psychocampa* species coded for morphology and sequenced in St Laurent et al. (2018a) to warrant this transfer.

76 *Psychocampamanalca* comb. n. possesses the genitalia typical of *Psychocampa* (*sensu*St Laurent et al. 2018a; based on dissection of genitalia NHMUK010402307 in NHMUK), and is thereby transferred to *Psychocampa* from *Cicinnus*. All of our morphological phylogenetic analyses support this new combination as *P.manalca* was consistently recovered nested within a broader *Psychocampa* + *Biterolfa* clade.

77 *Psychocampamarona* was transferred to *Psychocampa* from *Cicinnus* by St Laurent et al. (2018a) on morphological and molecular grounds. This species displays external and genital characteristics most similar to *P.bactriana*, *P.batesii*, *P.candacus*, *P.callipius*, *P.gaujoni, P.magnapuncta*, and *P.narseres*, therefore these species are herein transferred to *Psychocampa*.

78 *Psychocampamulatro* comb. n. was first noted to be related to *P.undiscata* by Dognin (1923). Although *P.mulatro* is rare in collections, the first author recently located several males belonging to this species (other known specimens are female) among the reared material originating from D. Janzen and W. Hallwach’s rearing efforts in Guanacaste, Costa Rica, deposited in the USNM. Based on external morphology and genitalia (examined from brushing), *P.mulatro* undoubtedly belongs in *Psychocampa* and we transfer this species here.

79 *Psychocampaunalca* was transferred to *Psychocampa* from *Cicinnus* by St Laurent et al. (2018a) on molecular and morphological grounds.

80 *Psychocampaundiscata* stat. rev. was erroneously synonymized with *P.joanna* in Becker (1996), who incorrectly listed the type locality of *P.undiscata* as “Fr. Guiana” which gave the appearance of a valid synonymy considering the type locality of *P.joanna* is French Guiana. However, the correct type locality of *P.undiscata* is São Paulo, Brazil, and the morphology of this southeastern Brazilian and Argentinian species is noticeably different from the Amazonian *P.joanna* and Central American *P.lacuna*. Namely in *P.undiscata* (Franclemont dissections 1431, 1432 in CUIC) the gnathos arms are thicker and more robust than in *P.joanna*, and in this way more closely resemble *P.lacuna*. Furthermore, the phallus of *P.undiscata* is substantially flattened, wide, and spoon-like, immediately distinguishing it from related species. Dark, chocolate brown coloration and distribution in southeast Brazil and Argentina also help distinguish *P.undiscata* from the comparatively lighter colored Amazonian *P.joanna* and even lighter Central American *P.lacuna*. We therefore revalidate this taxon. Because the previous status of the name *undiscata* was as a synonym of “*Cicinnusjoanna*” in Becker (1996), and because *joanna* was transferred to *Psychocampa* along with its associated synonyms by St Laurent et al. (2018a), the present combination of *Psychocampaundiscata* is not a new combination, but a revived status.

81 *Psychocampaviemanda* comb. n. is externally difficult to distinguish from *P.unalca*, but *P.viemanda* is larger with darker maculation and has wider submarginal areas. Considering the close relationship of *P.unalca* and *P.viemanda*, and the incontrovertible evidence placing *P.unalca* in *Psychocampa* based on external morphology and molecular data in St Laurent et al. (2018a), we hereby transfer *P.viemanda* to *Psychocampa*. The genitalia of *P.viemanda* are also typical of *Psychocampa*. All of our morphological phylogenetic analyses support this new combination as *P.viemanda* was consistently recovered nested within a broader *Psychocampa* + *Biterolfa* clade.

82 The name *melini* syn. n. was proposed as a subspecies of *P.joanna* by Bryk (1953), and the name was omitted by Becker (1996). Examination of the external morphology of the type of *melini* revealed that it is not conspecific with *P.joanna*, but rather with *P.viemanda*, and we hereby synonymize the name *melini* with *viemanda*. This new synonymy is not also a new combination because St Laurent et al (2018a) had already transferred *P.joanna* to *Psychocampa*. Examination of the genitalia of the type revealed structures typical of *Psychocampa*, and not largely distinct from those *P.unalca* or *P.joanna*. Genitalia of many *Psychocampa* species are nearly identical even when external morphology is very distinct between species. For example, *P.joanna* is more externally distinct from *P.unalca* and *P.viemanda*, than *P.unalca* is from *P.viemanda*. However, the genitalia of all three species are essentially the same. *Psychocampa* have some of the most homogenous genitalia structures of any mimallonid genus, even the sister genus *Biterolfa* display genitalia characters largely diagnostic of *Psychocampa*.

83 *Bedosiadulcis* comb. n. is hereby transferred to *Cicinnus* based on a genitalia examination of a Costa Rican specimen (genitalia vial NHMUK010402311 in NHMUK). Genitalia of *B.dulcis* are quite similar in all respects to the genitalia of *B.fraterna*, the type species of *Bedosia*. Although maculation of *B.dulcis* is unlike other *Bedosia* species, the presence of a straight postmedial line, hyaline patch on the forewings (absent from hindwings) and overall wing shape are congruent with the diagnostic characters given for Bedosiini in St Laurent et al. (2018a). All of our morphological phylogenetic analyses support this new combination as *B.dulcis* was consistently recovered nested within *Bedosia*.

84 *Bedosiaeuthymius* comb. n. is most similar in external morphology to *B.dulcis*, more so than any *Cicinnus* and is therefore transferred to *Bedosia*. We have also examined the genitalia, confirming placement of *euthymius* in *Bedosia*. Ongoing molecular phylogenomics which include *B.euthymius* furthermore supports this transfer (St Laurent et al. in prep.).

85 Herbin (2016) designated a lectotype for *B.itamaraty*, figuring the specimen. Apparently the original syntypes of *B.itamaraty* includes multiple species according to Herbin (2016).

86 *Bedosiaolasis* comb. n. is similar to *B.dulcis*, as originally mentioned by Schaus (1928), and is therefore transferred to *Bedosia*. This is a relatively rare species in collections, and unfortunately the type locality, “Central America”, is not specific, though the first author is aware of a potentially conspecific species that is commonly collected in Guatemala (several specimens in MGCL and CRAS). This species appears to be closely related to *B.dulcis* and *B.euthymius* which are found in Central America and western Colombia respectively, suggesting a unique Central American/western Andean lineage of *Bedosia* lacking morphologically similar species in other regions of South America. Examination of the genitalia of the type of *B.olasis* (by brushing) and full dissections of the Guatemalan population, displays genitalia typical of *Bedosia*.

87 Authorship of *B.strigifera* (and other Mimallonidae described by C. and R. Felder (1874)) was determined with the aid of Nässig and Speidel (2007), hence it is in disagreement with the authorship listed in Becker (1996). The type locality of *B.strigifera* is given only as “Brazil” but the type specimen in the NHMUK is a good match for a common southeastern Brazilian species, therefore the original collecting locality of *B.strigifera* was likely one of the heavily collected regions from the 1800s such as Rio de Janeiro or São Paulo.

88 In the original description, Schaus (1928) stated that the type deposition for *B.forbesi* was the CUIC. However, we confirm that the type is not currently in the CUIC and is instead in the USNM.

89 The species *Bedosiallogentilis* comb. n. and *B.minimalis* comb. n., previously both placed in *Cicinnus*, display genitalia characteristics very close to *Bedosiallo*. At the time of the *Bedosiallo* description, St Laurent et al. (2018a) did not realize this close similarity, and thus did not place these two species in this genus. We hereby transfer these two species to *Bedosiallo*. Upon examining the male genitalia of *B.minimalis*, and the genitalia of both sexes of two undescribed species near *B.gentilis* (*B.gentilis* is known to the first author only by the female type), it is clear that these species share a close relation to those placed in *Bedosiallo*, namely in the external appearance in which the hyaline discal spot is lined with dark scales (a trait shared with most Bedosiini) and the wing veins which are accentuated by light brown scales. The male genitalia are quite similar to those in the *B.forbesi* species-group, both have a large, concave juxtal complex attached dorsally to the phallus. The simple triangular valvae and robust gnathos are also similar to those described from *Bedosiallo*, although in *B.cfgentilis* and *B.minimalis* the gnathos arms appear distally fused. However, upon close examination, they are slightly separated as in all other *Bedosiallo*, only substantially thicker and shorter. In an undescribed species of *Bedosiallo* near *B.gentilis* from Southeast Brazil, the gnathos arms are fully fused. The phallus of these newly included species is thicker than in all others in the genus, and contain cornuti. *Bedosiallominimalis* was consistently placed within Bedosiini in all morphological phylogenetic analyses, and within *Bedosiallo* in all but the constrained ML analysis (in which *B.minimalis* was placed sister to *Bedosia* + *Bedosiallo*). Regardless, *Bedosiallo* is the most appropriate genus based on morphology for these two species. Because of the noted unique features of *B.gentilis* and *B.minimalis*, we place them within a third species group within *Bedosiallo*.

90 *Aceclostriacordubensis* comb. n. displays the complex asymmetrical genitalia typical of *A.mus*, type species of *Aceclostria*. In *A.cordubensis* the complicated juxtal arrangement has more robust and more distinctly curved dorsal processes, a shorter spine that protrudes over the phallus, and the gnathos arms are smaller than in *A.mus*; but otherwise these features are extremely similar to all examined *Aceclostria* dissections examined thus far. Externally *A.cordubensis* is earthen in coloration, with indistinct suffuse maculation, and postmedial lines arranged in much the same way as in *A.mus*. Furthermore, in *A.cordubensis* and *A.mus*, the discal cell varies from fully scaled to completely hyaline in specimens collected from the same series. Such pronounced intraspecific variation in hyaline patches appears to be so far unique to *Aceclostria*. Distribution in dryer regions of central South America and usage of *Schinus* Linnaeus (Anacardiaceae) as host plants also supports the inclusion of *A.cordubensis* in *Aceclostria* (Schreiter 1943). Although the type of *A.cordubensis* has not been located in collections in Argentina or Europe (F. Penco pers. comm., St Laurent pers. obs.), the type of its synonym, *schulzii*, was found in the MNHU. Despite the missing type of *A.cordubensis*, the identity of this species is not in question due to the fact that both *cordubensis* and *schulzii* come from the same type locality (Cordobá, Argentina) and figures of *A.cordubensis* in Schreiter (1943) and the type specimen of *schulzii* appear to be conspecific. *Aceclostriacordubensis* is one of the few Mimallonidae found far south in Argentina (Córdoba), and additional specimens examined by the first author in the ZSM from localities even farther south in Argentina and near the Chilean border, are a close match to *A.cordubensis*.

91 A specimen of *Aceclostriamus* labeled as “*Cicinnuscallinica*‡ Schaus” is present in the USNM. This specimen apparently represents an unpublished manuscript name because no description exists in the literature using this name and this specimen, and therefore is an unavailable name. The specimen is from the same type locality as *A.villaricensis*, which is a synonym of *A.mus*. It is worth noting that “*Cicinnuscallinica*‡” should not be confused with the valid and obviously distinct *Trogopteracallinica* Schaus.

92 Upon dissecting *A.mus* specimens from the type localities of both *mus* (São Paulo, Brazil, genitalia preparation NHMUK010402316 in NHMUK) and *villaricensis* (Villarica, Paraguay genitalia preparation NHMUK010402317 in NHMUK), we determined that there are no external nor male genitalia differences between these populations and maintain the synonymy of Becker (1996). Other populations from the Cerrado of Central Brazil, however, do differ externally and in genitalia, and likely represent an undescribed species.

93 *Aceclostrianigrescens* stat. rev. et comb. n. has been treated as a synonym of *A.cordubensis* since Schaus (1928). Upon examination of “true” *A.cordubensis* from central and southern Argentina, it is clear that *A.nigrescens* has significantly less falcate forewing apices and distinct maculation patterns when compared to *A.cordubensis*. Furthermore, *A.nigrescens* is known only from Castro, Paraná, Brazil where *A.cordubensis* does not occur (St Laurent pers. obs.). These reasons permit us to revive the status of *C. nigrescens* as a valid species.

94 Authorship of *Aleydaheppneri* is St Laurent, McCabe, and Malm, 2018 as per St Laurent et al. (2018b), but the authorship was erroneously attributed to “St Laurent and McCabe” for the holotype label data in that article. The actual holotype label and throughout the rest of the article correctly reads “St Laurent, McCabe, and Malm, 2018”. Furthermore, in the abstract of St Laurent et al. (2018b) it reads: “*Aleydaheppneri* St Laurent, McCabe and Malm, sp. n. from Panamá and French Guiana is newly described” but “Panamá” should be replaced with Peru as the type locality of *A.heppneri* is in Peru, not Panama. The correct type locality information is given elsewhere in the article.

95 We hereby transfer *Arcinnusxingua* comb. n. from *Cicinnus* upon examination of the genitalia of the type specimen in the USNM (St Laurent diss.: 4-16-18:3 in USNM), which displays characters consistent with *A.hoedli*, the type species of *Arcinnus*. We considered the possibility that *A.hoedli* could be a junior synonym of *A.xingua*, but from comparing the genitalia of the type specimen of *A.xingua* to three dissections of *A.hoedli* (including the holotype dissection figured in Herbin (2016)), it is evident that the blunt vincular arms typical of *Arcinnus* are much more robust in the type of *A.xingua* than in any of the *A.hoedli* dissections. The valvae of *A.xingua* also terminate much more abruptly than in *A.hoedli*, forming a sharp distal edge. All of our morphological phylogenetic analyses support this transfer.

96 *Cicinnus* is the most species-rich mimallonid genus. A great deal of taxonomic confusion has been associated with *Cicinnus*, and the genus has largely existed as a wastebasket taxon since its inception. Much of the confusion of the identification of “true” *Cicinnus* can be traced back to the type species, *C.orthane*. This species, described from Chile, is one of the only mimallonids recorded from that country, and therefore has been speculated to be erroneously labeled (St Laurent et al. 2018c). Comparisons of the putative female syntype of *C.orthane*, which was subsequently designated as the lectotype, in the NHMUK, revealed external and genitalia morphology consistent with a common southeastern Brazilian species similar to *C.incerta*, *C.solvens*, and related species. Examination of males from the Brazilian population helped to solidify our understanding of the morphology of “true” members of this genus. St Laurent et al. (2018a) sampled two morphologically distinct *Cicinnus* species-groups in order to begin to understand the relationships of *Cicinnus*, but insufficient sampling in *Cicinnussensu stricto* and *Cicinnussensu lato* leaves many open questions about the systematics of *Cicinnus*. St Laurent et al. (2018a) recovered two robustly supported clades of *Cicinnus*: *Cicinnus* s. l. and *Cicinnus* s. s., the latter which included *C.incerta*, a close relative of type species *C.orthane*. This dichotomy of *Cicinnus* suggests at least two genera must be recognized to eliminate paraphyly in *Cicinnus* s. l. In order to reorganize *Cicinnus* for the present study, we break the genus into three morphologically distinct groups, and a fourth uncertain grouping lacking defining morphological characteristics. We do not formally assign names to these groups at this time, but separate them for organizational purposes. The group numbers are unrelated to Schaus’ (1928)*Cicinnus* numbering system. Group 1 is *Cicinnus* s. s. and includes the “true” *Cicinnus* as per the phylogenetic results of St Laurent et al. (2018a). The remaining *Cicinnus* s. l. may eventually be transferred to a revalidated *Gonogramma*, see Annotation 97 below.

97 *Gonogramma* was named by Boisduval (1872) in association with *Psychocampa*, and has since been considered, apparently only inexplicitly, a synonym of *Cicinnus*. *Gonogramma* was proposed without any associated species, so Fletcher and Nye (1982) gave *C.despecta* as the type species of the genus but suggested that *C.despecta* was congeneric with the type of *Cicinnus*, and hence inferred that *Gonogramma* and *Cicinnus* were synonymous. The generic combination was apparently overlooked in the following years as Becker (1996) maintained *C.despecta* in *Cicinnus* and did not provide *Gonogramma* in the list of synonymies of *Cicinnus*. St Laurent et al. (2018a) raised the possibility that *Gonogramma* could be revalidated to include the “*Cicinnussensu lato*” species that have hyaline patches on the wings since this feature, as well as the genitalia of these taxa, are distinct from those of *Cicinnus* s. s. Ongoing molecular research, which includes many more of the *Cicinnus* s. l. species, as well as the type species of *Gonogramma*, *C.despecta*, supports the use of *Gonogramma* and the eventual transfer of all *Cicinnus* s. l. (Groups 2–4) species to this genus, including at least one that does not have hyaline patches on the wings (St Laurent et al. in prep.).

98 Group 1, which we treat here as *Cicinnussensu stricto*, includes species lacking hyaline patches on both the fore and hindwings, diffuse coloration with heavy speckling of dark brown to black petiolate scales, and short mesal arms of the gnathos. The gnathos originates from near the dorsal juncture between the vinculum and the base of the uncus. Vincular arms may be present or absent (the only Cicinnini group to have this character vary). St Laurent et al. (2018a) sampled *C.incerta* and *C.melsheimeri* (the former being a proxy for *Cicinnus* type species *C.orthane*) and found them sister to each other in a robustly supported clade.

99 The year of description for *C.incerta* was incorrectly given as 1877 by Becker (1996), which was apparently when the first volume of Möschler’s “Beiträge zur Schmetterlings-Fauna von Surinam” was published, when in fact *C.incerta* was described in the second volume from 1878.

100 *Cicinnuslatris* was described from an indeterminate number of specimens from two localities in Costa Rica. Considering that syntypes and different localities are involved in the type series representing *C.latris*, we designate a lectotype for the male specimen labeled as the “type” in the USNM (Fig. 168). This specimen bears the following labels: Tuis CR/ Sept./ Collection W. Schaus/ Type No. 18958 [or 16958, the second digit is illegible] U.S.N.M/ USNM-Mimal: 1139/. A red handwritten label reading “LECTOTYPE♂ *Cicinnuslatris* Schaus, designated by St Laurent” has been added to the specimen.

101 The name *melsheimeri* was published twice by Harris in the same year (Harris 1841a,b), under two different combinations: *Saccophoramelsheimeri* and *Perophoramelsheimerii* respectively. Unfortunately, some authors (e.g., St Laurent and McCabe 2016, 2017) incorrectly referenced the *second* publication to use *melsheimeri*, which was Harris’ book *A report on the insects of Massachusetts, injurious to vegetation*, which was published in December of 1841 (Harris 1841b) with the combination *Perophoramelsheimerii*. Doubleday published Harris’ original description (via a correspondence from Harris) of *Saccophoramelsheimeri* earlier that year, in May of 1841 (Harris 1841a). Hopefully our clarification here will prevent others from making this mistake. Neither *Perophora* nor *Saccophora* are valid names as both are preoccupied. Grote (1896) proposed *Ptochopsyche* as a replacement name for *Perophora*, but this name is a synonym of the senior name *Cicinnus* Blanchard, 1852 since both *C.melsheimeri* and type species *C.orthane* are congeneric.

102 St Laurent and McCabe (2017) provided discussion regarding the names *egenaria* and *primolus*, confirming the synonymy of *egenaria* and *primolus* with *C.melsheimeri*.

103 As previously mentioned in Annotation 96, the type locality of *C.orthane* may be erroneous. Mimallonidae are exceedingly rarely collected in Chile, with only one other record of the family reported by Ureta (1957). There are also no mimallonid specimens in the major collection in Santiago, Chile (C. Mielke and A. Ugarte pers. comm.). The first author has not seen any mimallonid specimens from Chile despite examining specimens in over 50 institutional and private collections in Europe, Latin America and North America. A specimen labeled “*Cicinnusorthane* Chili” in the NHMUK has been designated as a lectotype by St Laurent et al. (2018c). External and genital morphology of the lectotype matches that of a common southeastern Brazilian species that has gone unnamed, and thus it is possible that the original specimen of *C.orthane* was of Brazilian origin and was mislabeled. Assuming that the lectotype is the same specimen on which Blanchard based his illustration and description, the concept of *Cicinnus* s. s. is solidified.

104 *Cicinnussolvens* was described from three syntypes, two male and one female, from two different localities in Panama. Considering that syntypes and different localities are involved in the type series representing *C.solvens*, we designate a lectotype for the male specimen labeled as the “type” in the USNM (Fig. 169). This specimen bears the following labels: CabimaPan May 20. .11 August Busck/ Type No. 16102 U.S.N.M/ USNM-Mimal: 1038/. A red handwritten label reading “LECTOTYPE♂ *Cicinnussolvens* Dyar, designated by St Laurent” has been added to the specimen.

105 *Cicinnustuisana* was described from an indeterminate number of specimens from two localities in Costa Rica. Considering that syntypes and different localities are involved in the type series representing *C.tuisana*, we designate a lectotype for the male specimen labeled as the “type” in the USNM (Fig. 170). This specimen bears the following labels: JuanVinas CR/ Jan 09/ *Cicinnustuisana* Type Schaus/Type No. 16956 U.S.N.M/ USNM-Mimal: 1138/. A red handwritten label reading “LECTOTYPE♂ *Cicinnustuisana* Schaus, designated by St Laurent” has been added to the specimen.

106 Group 2 (*Cicinnus* s. l., in part) contains species recognizable by hyaline patches on the forewings or on both fore and hindwings, as well as by the absence gnathos arms and the presence of vincular arms in the male genitalia. These taxa will likely eventually be placed in a revalidated *Gonogramma* once molecular phylogenetics can more robustly determine the relationships within Cicinnini.

107 The first author dissected a topotypical male specimen of *C.belaria* comb. n. (St Laurent diss.: 3-15-16:1 in USNM) and hereby transferred this species *Cicinnus* from *Psychocampa* due to genitalia morphology, which are largely indistinguishable from *C.despecta*. Upon close examination of external maculation, it is apparent that *C.belaria* also resembles *C.despecta* externally, differing primarily in more obscure coloration and in size and narrowness of the forewings. All of our morphological phylogenetic analyses support this transfer, consistently placing *C.belaria* within a broader *Cicinnus* s. l. clade.

108 *Cicinnuscorallina* was described from three syntypes from Colombia and Panama. Because syntypes and specimens from different localities are part of the type series of *C.corallina*, we designate a lectotype for the male specimen labeled as the “type” in the USNM (Fig. 167). This specimen bears the following labels: Pacho, Colombia Ost-Cordill. 2200 m Coll. Fassl/ Dognin Collection/ *Cicinnuscorallina* Type♂ Dognin./ Type No. 29684 U.S.N.M/ USNM-Mimal: 1023/. A red handwritten label reading “LECTOTYPE♂ *Cicinnuscorallina* Dognin, designated by St Laurent” has been added to the specimen.

109 As for *Meneviaplagiata* (Annotation 5), the original primary type of *C.despecta* is lost, along with many types described by Walker originating from the Fry collection (Becker 2001). However, Walker’s (1855) original description clearly refers to subsequent authors’ and our concept of *C.despecta*, namely by the presence of a “broad red dorsal stripe” of the abdomen and the “red band” accompanying the outer edge of postmedial lines, characters that do not fit any other mimallonid species known from Rio de Janeiro. Considering the broad distribution of *C.despecta* s. l., and the various names treated as synonymous with *C.despecta* (but from distant type localities) we hereby designate a neotype for a specimen from Rio de Janeiro in NHMUK that most accurately reflects Walker’s description of this species, and thus establishes nomenclatural stability to the name *despecta* (Fig. 171). The specimen’s labels are as follows (individual labels separated by forward slashes): NEO-TYPE [circular, red-edged label]/Rio Janeiro 79·56 (reverse side *Perophorasanguinolenta* Felder)/ NEOTYPE Mimallodespecta Walker, 1855 designated by St Laurent [handwritten red label]/ NHMUK010895844/. Note that the name “*sanguinolenta*” is on one of these labels, but this name was commonly applied to specimens of *C.despecta* in the NHMUK by historical workers. The specimen that we designate as the neotype is not a syntype of *C.sanguinolenta*. The true syntype of *C.sanguinolenta* is a specimen of known location (deposited in the NHMUK), and bears very different labels of Felder’s time. The three synonyms (*sensu*Becker (1996)) of *C.despecta* come from three localities: *curtisea* from Argentina, *sanguinolenta* from Brazil, and *sachinius* from Minas Gerais, Brazil. After comparing the type of *sanguinolenta* to the neotype of *despecta*, it is clear that *sanguinolenta* should remain a synonym of *C.despecta*. However, the type of *curtisea* is not known to us, and the type of *sachinius* appears quite distinct from the typical *C.despecta*. Therefore, we are unable to clearly rectify the synonymy of these names until a thorough revision of *Cicinnus* s. l. is completed.

110 *Cicinnuscurtisea* potentially represents a valid species considering the geographic separation of the type localities of *C.curtisea* and *C.despecta* (see also Annotation 109). The type specimen(s) of *C.curtisea* is/are not known to us, however, other specimens from Buenos Aires are distinct from those from southeastern Brazil.

111 *Cicinnussanguinolenta* is a clear synonym of *C.despecta*, see also Annotation 109.

112 *Cicinnussachinius* is potentially a valid species considering the geographic range and morphological differences between the type of *C.sachinius* and *C.despecta*, see also Annotation 109.

113 *Cicinnuseminens* was transferred to *Cicinnus* from *Psychocampa* by St Laurent et al. (2018a) on molecular and morphological grounds.

114 *Cicinnusroscida* syn. rev. was synonymized with *C.externa* in Dognin (1924), but this synonymy was overlooked by Schaus (1928), Gaede (1931), and Becker (1996). *Cicinnusroscida* was treated as valid as recently as Becker (1996), and we hereby revive this synonymy upon examining numerous specimens of this species and the type of *C.roscida*.

115 Although *C.manicora* comb. n. displays valva and phallus shapes rather unique to this species, the presence of vincular arms, absence of gnathos arms, and presence of hyaline patches on the forewings all are clear indicators of *Cicinnus* s. l. Group 2, and we therefore transfer this species accordingly from *Psychocampa*, where it was placed apparently due to overly narrowed wings and sphingiform shape, characters that are superficially similar to some *Psychocampa*. All of our morphological phylogenetic analyses, as well as ongoing molecular phylogenomics that include this species (St Laurent et al. in prep.), support this transfer, consistently placing *C.manicora* within a broader *Cicinnus* s. l. clade.

116 Schaus (1928) suggested that *C.thermesia* may be a synonym of *C.corcovada*, and that the types are merely the male and female (respectively) of the same species. Both species are similar, but upon examining several specimens in the NHMUK and USNM and comparing these specimens directly to type material, we believe these two names belong to separate species. *Cicinnuscorcovada* displays slightly more rounded forewings, generally with a more convex postmedial line, and redder coloration, whereas *C.thermesia* has more angulate wings, often straighter postmedial lines, and a grayer coloration. A comparison is more easily made between the females of the two species. Unfortunately, whoever dissected the holotype of *C.thermesia* did not properly mount the entire genitalia (only abdominal segments are mounted on the slide); therefore, it will be impossible to accurately identify *C.thermesia* with a proper examination of type genitalia.

117 Group 3 (*Cicinnus* s. l., in part) contains species recognizable by hyaline patches on both the forewings and hindwings, as well as by the presence of gnathos and vincular arms in the male genitalia. Most species in this group were either already described in *Cicinnus*, or were transferred to *Cicinnus* from *Psychocampa* by St Laurent and Cock (2017) based on male genitalia morphology. These taxa will likely eventually be placed in a revalidated *Gonogramma* once molecular phylogenetics can more robustly determine the relationships within Cicinnini.

118 St Laurent and Cock (2017) transferred *C.beta*, along with several other species treated here in *Cicinnus* s. l. Group 3, to *Cicinnus* from *Psychocampa* based on genitalia morphology. However, these authors incorrectly listed this taxonomic change as “comb. n.” when in fact this was a revived combination since Schaus (1910) had originally described this species in *Cicinnus*.

119 *Cicinnusgaia* displays bizarre, atrophied valvae, seemingly making this species appear not to be part of *Cicinnus* s. l. Group 3. However, all characters unique to Group 3 are present in *C.gaia*, including vincular and gnathos arms, and hyaline patches on the fore and hindwings. Tornal diffusions that are present in the above species are also present in *C.gaia*, but are less discernable due to the lack of contrast in this more darkly colored moth.

120 *Cicinnusbibula* comb. n. is a very rare species in natural history collections. We are aware of only seven specimens in collections, and all appear to be female, although one in the MNHN (only examined via photograph) may be male. Considering the rarity of this species, and our inability to examine male genitalia, we preliminarily transfer *C.bibula* to *Cicinnus* s. l. from *Psychocampa* due to the presence of hyaline patches on the forewings, a character absent from all *Psychocampa*. Examination of female genitalia of *C.bibula*, reveals characters quite similar to those of female *Cicinnus* s. l., *Isoscella*, and *Roelmana*, namely the longitudinal wrinkles of the lamella antevaginalis and setae covered lobes on either side of the lamella antevaginalis. These same characters are characteristic of at least three Cicinnini genera, therefore although our generic placement of *C.bibula* is preliminary, the tribal assignment is most certainly correct.

121 The revived placement of *Cicinnuslemoulti* comb. rev. in *Cicinnus* remains uncertain. Externally, *C.lemoulti* is similar to Group 2 of *Cicinnus* s. l., particularly *C.externa*, and displays the typical forewing hyaline patch of this group. However, genitalia are unusual in this species, as it has a broadly triangular uncus, irregularly shaped valvae (Supplementary File 3: Plate 5, left ¾ of genitalia shown), and a phallus with a broad, ridged hammerhead-like dorsal projection (Supplementary File 3: Plate 4). The gnathos, however, is typical of *Cicinnus* s. l. Regardless, *C.lemoulti* most certainly does not belong in *Psychocampa* based on hyaline patch presence and genital morphology. Furthermore, all of our morphological phylogenetic analyses support this transfer, consistently placing *C.lemoulti* within a broader *Cicinnus* s. l. clade.

122 *Cicinnusmawaja* comb. n. is a particularly rare mimallonid, about which we know very little. We have been able to examine the genitalia of two male specimens, genitalia vial NHMUK010402304 and via Daniel Herbin (pers. comm.). The gnathos morphology, absence of vincular arms, and presence of unique saccular extensions (Supplementary File 3: Plate 4) are confounding structures that do not, together, fit any of our groups of *Cicinnus* s. l., and in some ways actually appear more similar to the species belonging to the *Roelmana* + *Isoscella* clade. Based on morphology, this species does not belong to *Psychocampa* where it has long been placed. Due to the lack of typical genitalia characters of Psychocampini, and presence of vincular apodemes (an apomorphy of Cicinnini) we preliminarily transfer this species to *Cicinnus* s. l. to rectify the tribal placement of this enigmatic taxon. All of our morphological phylogenetic analyses support this preliminary transfer, consistently placing *C.mawaja* within a broader *Cicinnus* s. l. clade. Ongoing molecular phylogenomics of Mimallonidae, which includes *C.mawaja*, support the inclusion of this taxon within the genus that will likely eventually be defined as *Gonogramma*, see Annotation 97. This is surprising considering the absence of hyaline patches on the wings, a character observed in all other *Cicinnus* s. l.

123 Originally described as a subspecies of *Euphanetadivisa*, *E.romani* was raised to full species status by Herbin (2016) based on external morphology and male genitalia.

124 *Isoscellaventana* was treated as belonging to *Psychocampa* by Schaus (1928), Gaede (1931), and Becker (1996). St Laurent and Carvalho (2017b) recognized the distinctness of this species, as well as three additional similar new species, and therefore described a new genus in which to place them. St Laurent et al. (2018a) included *I.ecuadoriana* in their phylogenetic analysis and found *Isoscella* to belong to a distinct lineage including several species also postulated by St Laurent and Carvalho (2017b) as probably belonging to *Roelmana*, the sister genus of *Isoscella*. Therefore, the validity of *Isoscella* as a genus distinct from *Psychocampa* has been well supported. See the genus treatment remarks for *Isoscella* above regarding conflicting topological placement of the most unique *Isoscella* species, *I.andina*.

125 Long considered monotypic, *Roelmana* was found to likely include several disparate species by St Laurent and Carvalho (2017b) after considering similar genitalia characters among these species, although these authors did not make any new combinations. St Laurent et al. (2018a) found *R.maloba* to form a lineage sister to *Isoscella* as previously postulated by St Laurent and Carvalho (2017b), and furthermore forming a clade with *R.laguerrei* and *R.pluridiscata* which both display the same uniting male genitalia characters referenced by St Laurent and Carvalho (2017b). St Laurent et al. (2018a) implemented taxonomic changes for the two species found to form a clade with *R.maloba*: *R.laguerrei* and *R.pluridiscata*, transferring them to *Roelmana*. We hereby transfer the remaining disparate species mentioned by St Laurent and Carvalho (2017b). *Roelmana* contains two distinct groups of species, which may formally be determined to be species-groups or genera in a revision of *Roelmana* or a more densely sampled phylogeny. The typical group includes species with hyaline patches on the fore and hindwings (as in the sister genus *Isoscella*), whereas the other group contains species lacking hyaline patches.

126 Of all species previously and here transferred to *Roelmana*, *R.beneluzi* comb. n. displays the most divergent male genitalia, and may eventually prove to be more appropriately placed in a separate new genus. We hesitate to do so here pending ongoing molecular phylogenetic work that will likely be able to include *R.beneluzi*. The gnathos shape however, is consistent with other taxa placed in *Roelmana* here and in St Laurent et al. (2018a), and externally this species displays similar diffuse, weakly defined patterning as in *R.laguerrei* and *R.prominens*. Therefore, *Roelmana* is certainly a more systemically relevant generic assignment than *Cicinnus*. Furthermore, *R.beneluzi* was recovered sister to *R.laguerrei* in all morphological phylogenetic analyses, thus supporting this preliminary transfer.

127 Externally, *R.brasiliensis* comb. n. is nearly identical to the rare *R.fenestrata* comb. n., and in the original description of *R.brasiliensis* this similarity was unfortunately not addressed. *Roelmanafenestrata* is only known from the female type (erroneously reported and labeled as a male), collected in Castro, Paraná, Brazil, a locality that sits between Cerrado and Mata Atlântica. *Roelmanabrasiliensis* is a Cerrado endemic, therefore it is possible that these two names are synonymous. However, the hyaline discal marking on the hindwing is closer to the postmedial line in *R.fenestrata* than in females of *R.brasiliensis*. Pending the availability of male specimens from near the type locality of *R.fenestrata*, and the loss of the type genitalia of *R.fenestrata*, it is not possible to reliably synonymize these names at this time. All morphological phylogenetic analyses consistently recovered *R.brasiliensis* as sister to *R.vitreata* + *R.pluridiscata*, thus supporting this transfer of *R.fenestrata* which could not be coded for morphology for the abovementioned reasons.

128 *Roelmanadoralica* comb. n. is externally most similar to *R.pluridiscata* and *R.vitreata*, the three of which form an apparent species complex that is widely distributed throughout South America. We hereby transfer *R.doralica* and *R.vitreata* to *Roelmana* from *Psychocampa* as per St Laurent et al. (2018a) who found *R.pluridiscata* nested within the newly defined *Roelmana* clade. Genitalia of all three species have been examined to confirm this placement.

129 As discussed in the original description of *R.laguerrei*, *R.prominens* comb. n. is most similar to this species than any other in Mimallonidae, due to the purplish-blue coloration and male genitalia characteristics. St Laurent et al. (2018a) included *R.laguerrei* in their phylogeny, and found it placed within *Roelmana*. This result was unsurprising given the similarity in male genitalia to *R.maloba*, the type species of *Roelmana*. We here formally transfer *R.laguerrei* to *Roelmana*.

## Plates

**Figures 18–21.**
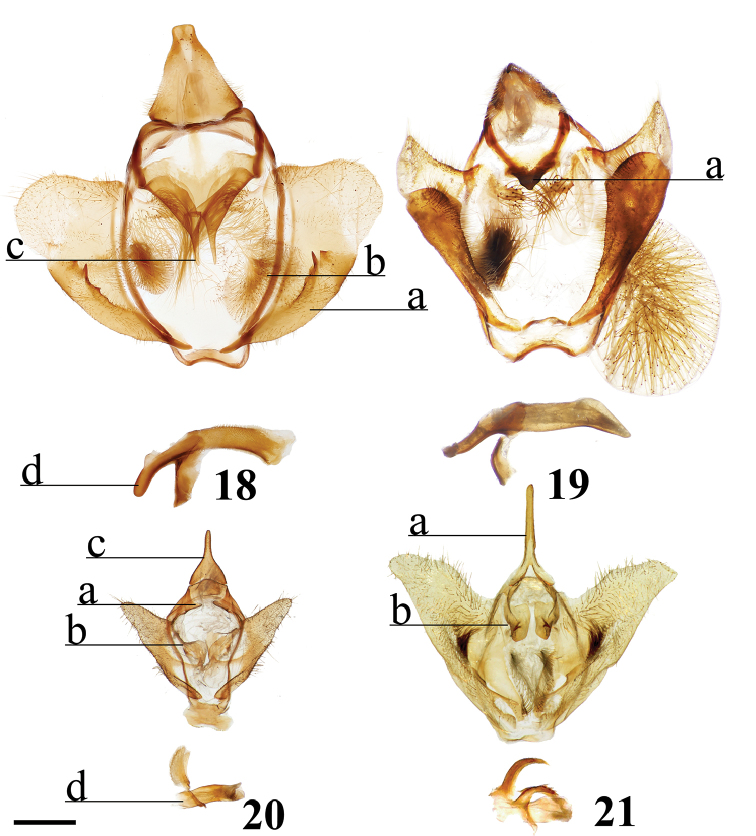
Ventral view of male genitalia of type species of genera belonging to Lacosominae: Trogopterini and Lacosomini. Phallus/juxtal complex is figured below the genitalia in the lateral view. **18***Reinmaraenthona*, St Laurent diss.: 5-18-16:1 (CNC) **19**Trogopteracfnotata, St Laurent diss.: 17-3-18:5 (MGCL) **20***Lacosomachiridota*, St Laurent diss.: 10-5-17:5 [reused with permission from St Laurent et al. (2018a), Systematic Entomology] (CRAS) **21***Vanengamera*, St Laurent diss.: 7-7-16:2 [reused with permission from St Laurent and Herbin (2017), ZooKeys] (NHMUK). Scale bar: 1 mm.

**Figures 22–26.**
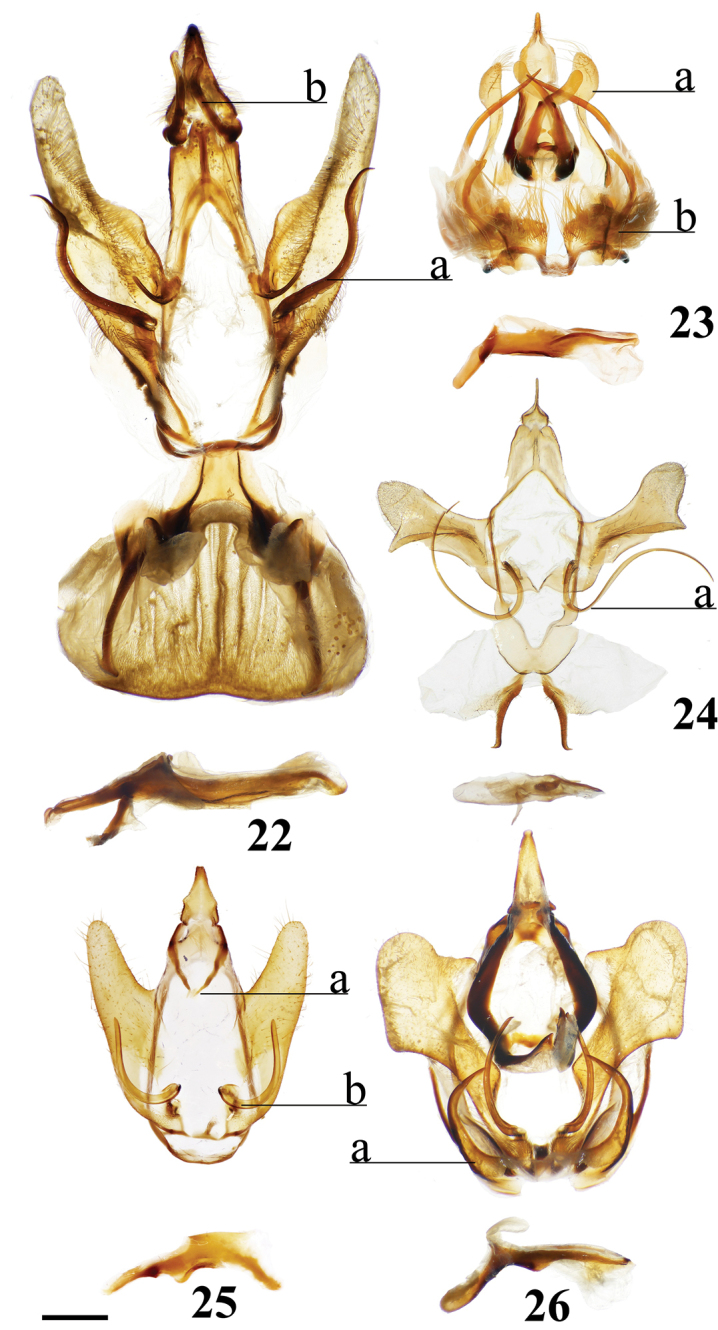
Ventral view of male genitalia of type species of genera belonging to Druenticinae: Druenticini. Phallus/juxtal complex is figured below the genitalia in the lateral view. **22***Druenticapartha*, St Laurent diss.: 3-17-18:3 (MGCL) **23***Micrallominutus* holotype, St Laurent diss.: 10-21-15:1 [reused with permission from St Laurent and Mielke (2016), ZooKeys] (DZUP) **24***Ulalumavalva*, St Laurent diss.: CPC 7 [reused with permission from St Laurent et al. (2018a), Systematic Entomology] (CRAS) **25***Pameaalbistriga* syntype, genitalia vial NHMUK 010402302 **26***Procinnuscahureli*, genitalia vial NHMUK 010402314. Scale bar: 1 mm.

**Figures 27, 28.**
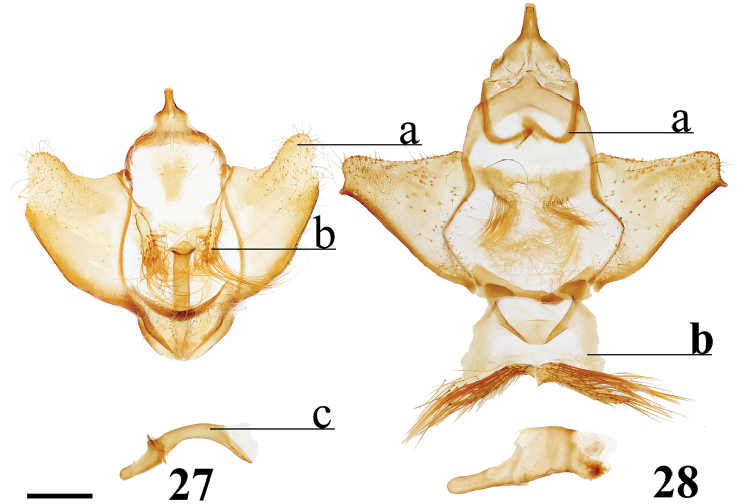
Ventral view of male genitalia of type species (except where noted) of genera belonging to Druenticinae: Luramini. Phallus/juxtal complex is figured below the genitalia in the lateral view. Figures are reused with permission from St Laurent (2016), ZooKeys. **27***Luramaquindiuna*, St Laurent diss.: 2-14-16:5, not the type species of *Lurama* (USNM) **28***Ulmararotunda*, St Laurent diss.: 4-5-16:3 (MNHU). Scale bar: 1 mm.

**Figures 29–32.**
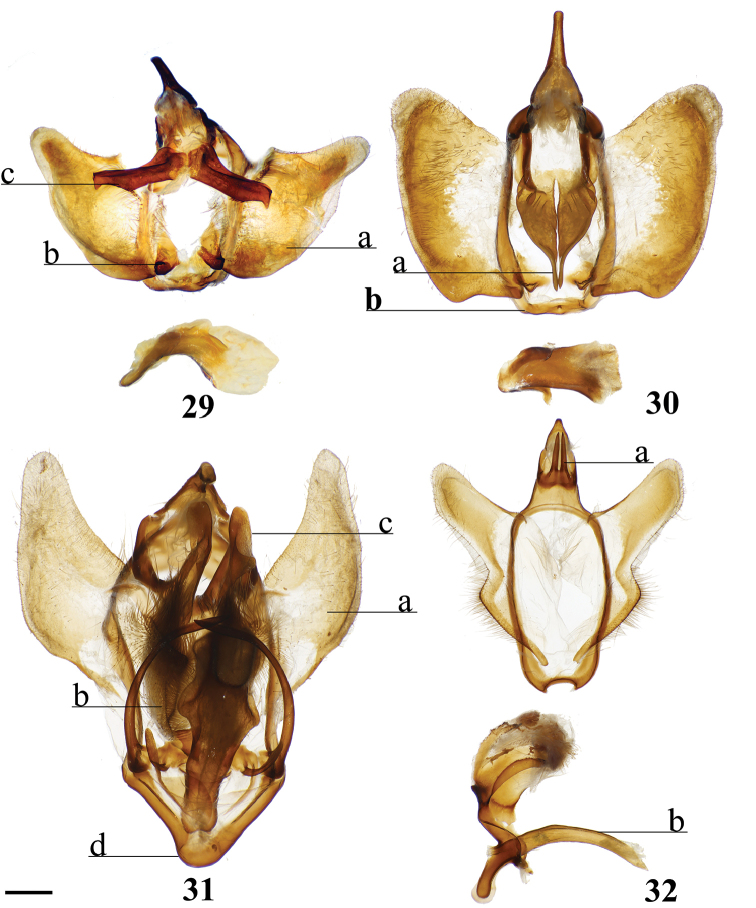
Ventral view of male genitalia of type species of genera belonging to Cicinninae: Psychocampini and Bedosiini. Phallus/juxtal complex, when excised, is figured below the genitalia in the lateral view. **29***Biterolfaalthea*, St Laurent diss.: 11-16:11 [reused with permission from St Laurent et al. (2017d), Tropical Lepidoptera Research] (USNM) **30***Psychocampaconcolor*, St Laurent diss.: LEP53954 [reused with permission from St Laurent et al. (2018a), Systematic Entomology] (MGCL) **31***Bedosiafraterna*, St Laurent diss.: 10-5-17:6 [reused with permission from St Laurent et al. (2018a), Systematic Entomology] (CRAS) **32***Bedosialloforbesi*, St Laurent diss.: 10-2-17:4 [reused with permission from St Laurent et al. (2018a), Systematic Entomology] (MGCL). Scale bar: 1 mm.

**Figures 33–39.**
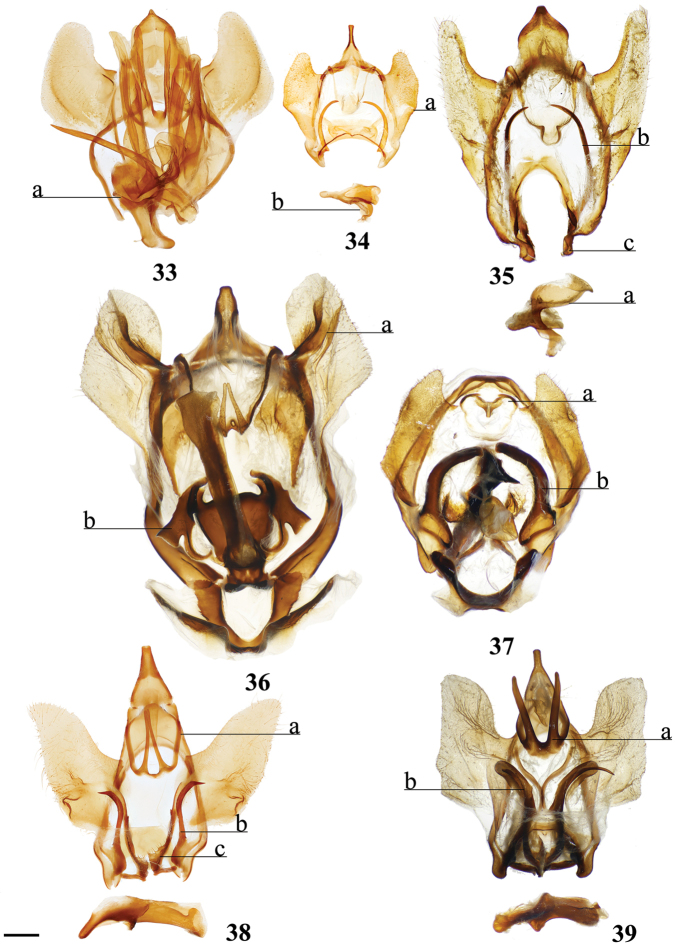
Ventral view of male genitalia of type species of genera belonging to Cicinninae: Cicinnini. Phallus/juxtal complex, when excised, is figured below the genitalia in the lateral view. **33***Aceclostriamus*, St Laurent diss.: 10-8-14:2 (CUIC) **34***Aleydaaccipiter*, genitalia preparation and photo by T Malm, diss.: NHRS-TOBI 000001871 [reused with permission from St Laurent et al. (2018b), SHILAP) (NHRS) **35***Euphanetadivisa*, St Laurent diss.: 3-17-18:1 (MGCL) **36**Cicinnuscforthane, St Laurent diss.: 10-30-17:1 [reused with permission St Laurent et al. (2018c), Zootaxa] (MGCL) **37***Arcinnushoedli*, St Laurent diss.: LEP20643 (MGCL) **38***Isoscellaventana*, St Laurent diss.: 4-29-16:2 [reused with permission from St Laurent and Carvalho (2017b), Journal of the Lepidopterists’ Society] (USNM) **39***Roelmanamaloba*, St Laurent diss.: 3-22-18:1 (MGCL). Scale bar: 1 mm.

**Figures 40–44.**
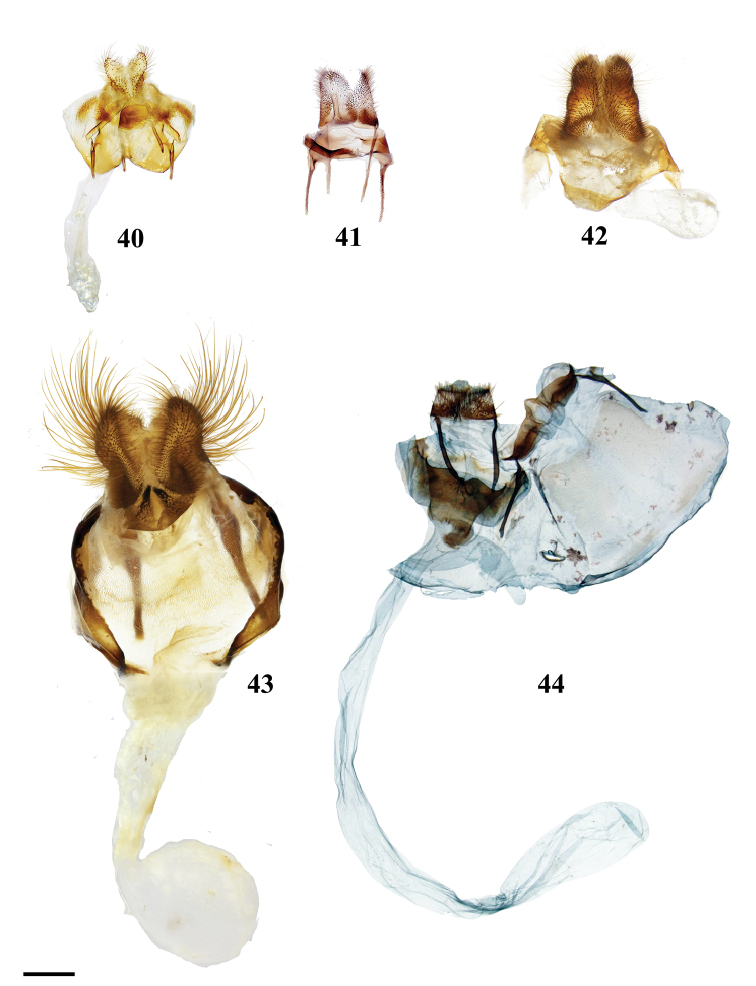
Ventral view of female genitalia of type species (except where noted) of genera belonging to Zaphantinae, *incertae sedis*, and Aurorianinae. **40***Zaphantainfantilis*, genitalia vial NHMUK 010402342 **41***Menevialantona*, St Laurent diss.: 7-8-15:1, ductus and corpus bursae missing [adapted and modified from St Laurent and Dombroskie (2016)] (USNM) **42***Tolypidaamaryllis*, St Laurent diss.: 3-31-18:1 (CRAS) **43***Roelofaolivia*, genitalia vial NHMUK 010402328 (NHMUK) **44***Aurorianaflorianensis*, C. Gibeaux diss. prep: 7759, not the type species of *Auroriana*, [reused with permission from St Laurent and Mielke (2016), ZooKeys] (MNHN). Scale bar: 1 mm.

**Figures 45–48.**
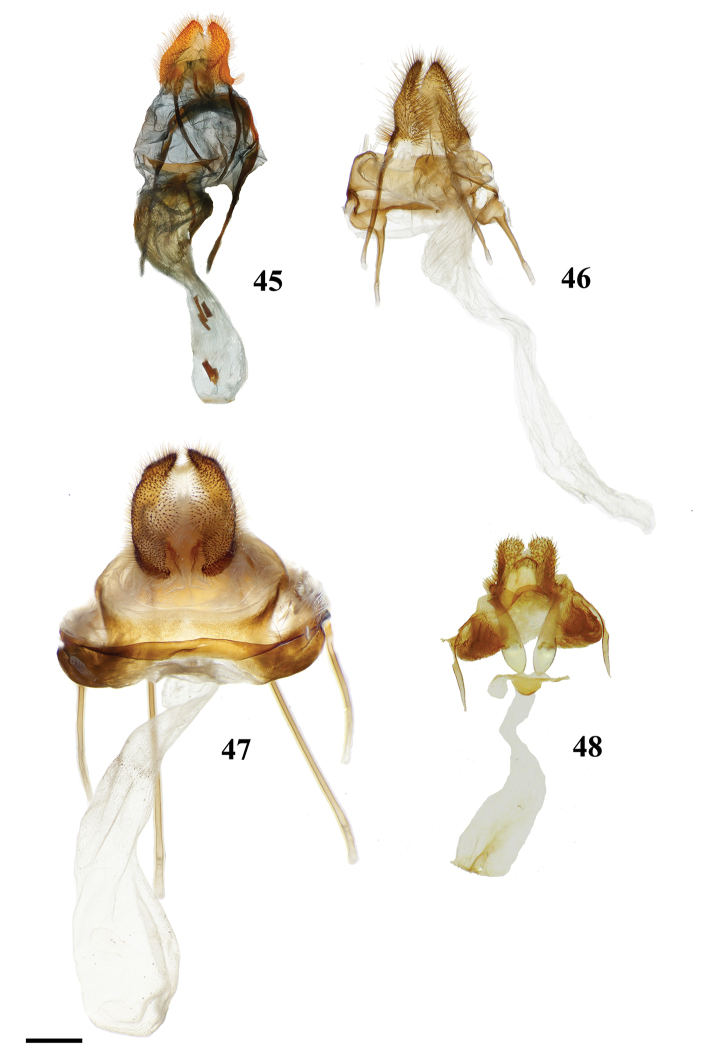
Ventral view of female genitalia of type species (except where noted) of genera belonging to Mimalloninae. **45***Eadmunaesperans*, St Laurent diss.: 2-12-16:1 (NHMUK) **46**Macessogacffabia, genitalia vial NHMUK 010402337 (NHMUK) **47***Mimalloamilia*, St Laurent diss.: 3-31-18:2 (MGCL) **48***Tostalloalbescens*, St Laurent diss.: 10-21-15:5 [reused with permission from St Laurent and Mielke (2016), ZooKeys] (NHMUK). Scale bar: 1 mm.

**Figures 49–53.**
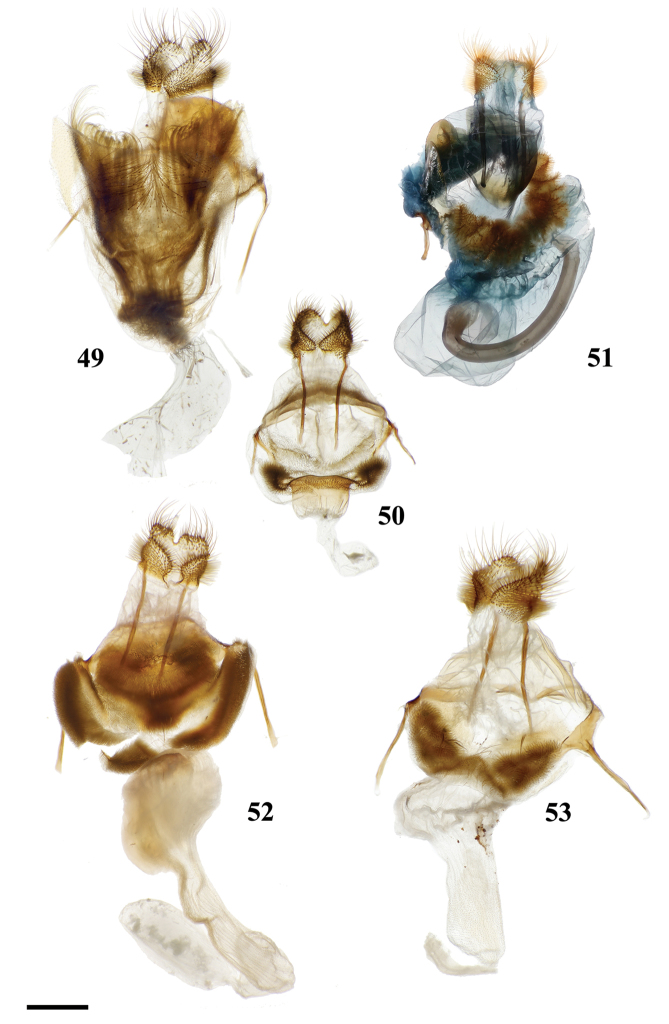
Ventral view of female genitalia of type species (except where noted) of genera belonging to Lacosominae: Alheitini. **49***Adalgisacroesa*, St Laurent diss.: 4-1-18:1 (CRAS) **50**Alheitacfpulloides, St Laurent diss.: 10-10-17:3, not the type species of *Alheita* (MGCL) **51***Taremarivara*, St Laurent diss.: 3-14-16:8 [reused with permission from St Laurent et al. (2017b), ZooKeys] (CNC) **52***Herbinallacaudina*, St Laurent diss.: 9-20-17:2 [reused with permission from St Laurent et al. (2018a), Systematic Entomology] (MGCL) **53***Thaelialinamariae*, St Laurent diss.: LEP50145 (MGCL). Scale bar: 1 mm.

**Figures 54–57.**
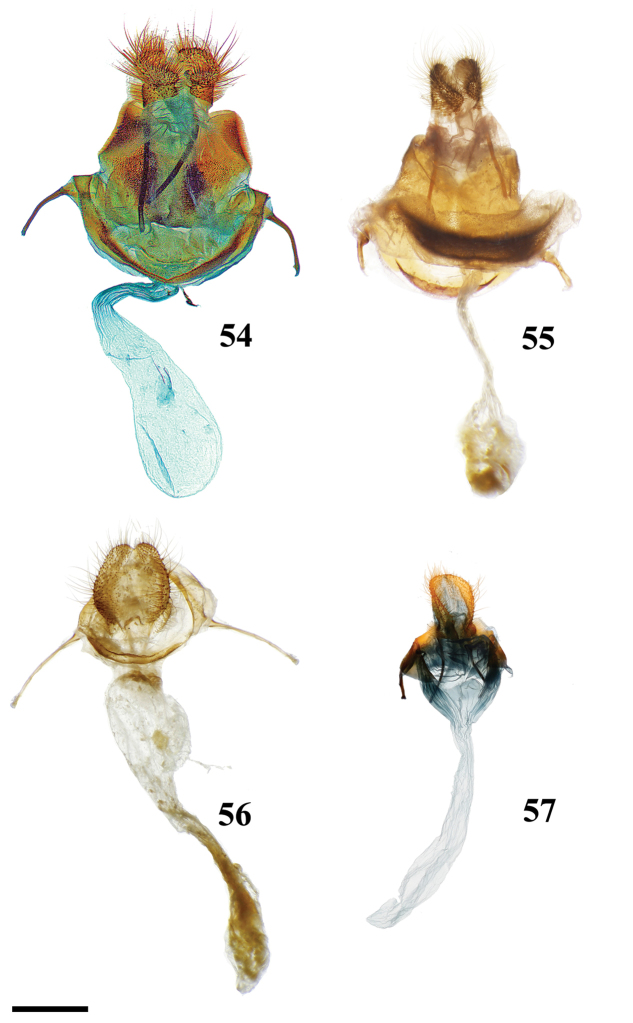
Ventral view of female genitalia of type species (except where noted) of genera belonging to Lacosominae: Trogopterini and Lacosomini. **54***Reinmaraenthona*, D. Herbin genitalia diss.: H. 1103 [reused with permission from St Laurent et al. (2017d), Tropical Lepidoptera Research] (CDH) **55***Trogopteratirzaha*, St Laurent diss.: 3-31-18:4, not the type species of *Trogoptera* (MGCL) **56***Lacosomachiridota*, St Laurent diss.: 3-31-18:3 (MGCL) **57***Vanengamera*, St Laurent diss.: 5-17-16:5 [reused with permission from St Laurent and Herbin (2017), ZooKeys] (CUIC). Scale bar: 1 mm.

**Figures 58–64.**
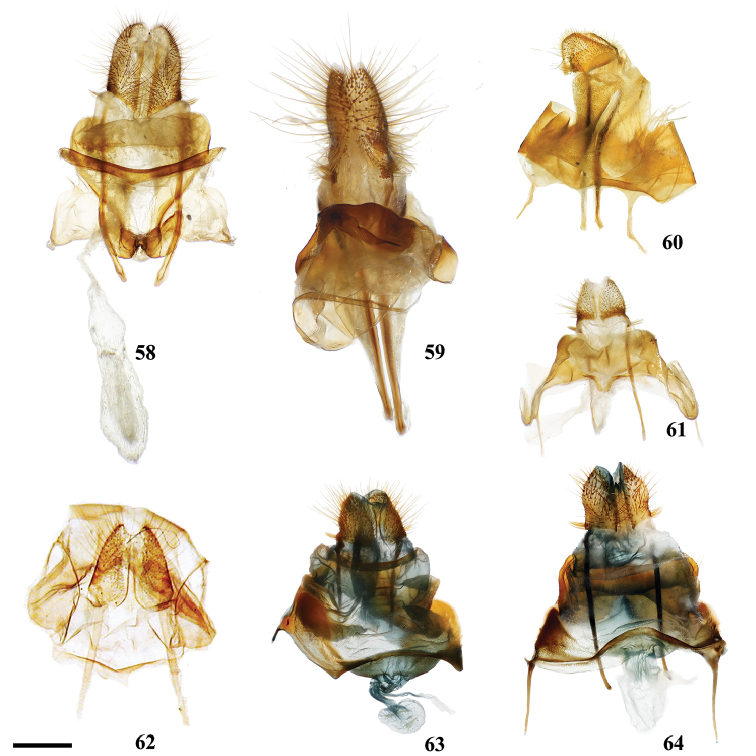
Ventral view of female genitalia of type species (except where noted) of genera belonging to Druenticinae. **58***Druenticapartha*, St Laurent diss.: 3-31-18:5 (MGCL) **59**Procinnuscfacuta, St Laurent diss.: LEP50136, not the type species of *Procinnus* (MGCL) **60***Micrallominutus*, St Laurent diss.: 10-21-15:2 [reused with permission from St Laurent and Mielke (2016), ZooKeys] (USNM) **61***Ulalumavalva*, St Laurent diss.: 10-10-17:2 [reused with permission from St Laurent et al. (2018a), Systematic Entomology] (MJWC) **62***Pamea* sp., Franclemont diss.: 1766, genitalia not to scale (CUIC) **63***Luramaquindiuna*, St Laurent diss.: 4-14-16:1, not the type species of *Lurama* [reused with permission from St Laurent (2016), ZooKeys] (USNM) **64***Ulmaraconjuncta*, St Laurent diss.: 4-19-16:1 [reused with permission from St Laurent (2016), ZooKeys] (MWM). Scale bar: 1 mm.

**Figures 65–68.**
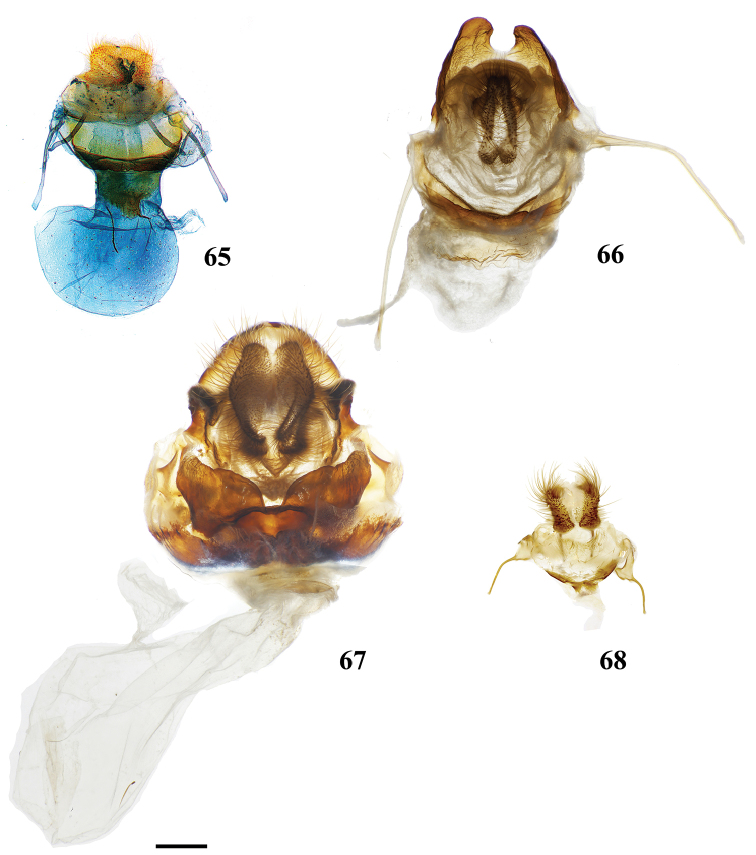
Ventral view of female genitalia of type species (except where noted) of genera belonging to Cicinninae: Psychocampini and Bedosiini. **65***Biterolfaalthea*, D. Herbin genitalia diss.: H600 [reused with permission from St Laurent et al. (2017d), Tropical Lepidoptera Research] (CDH) **66***Psychocampaconcolor*, St Laurent diss.: 3-31-18:7 (MGCL) **67***Bedosiaturgida*, St Laurent diss.: 3-31-18:6, not the type species of *Bedosia* (MGCL) **68***Bedosialloforbesi*, St Laurent diss.: genitalia vial NHMUK 010402292 [reused with permission from St Laurent et al. (2018a), Systematic Entomology] (NHMUK). Scale bar: 1 mm.

**Figures 69–74.**
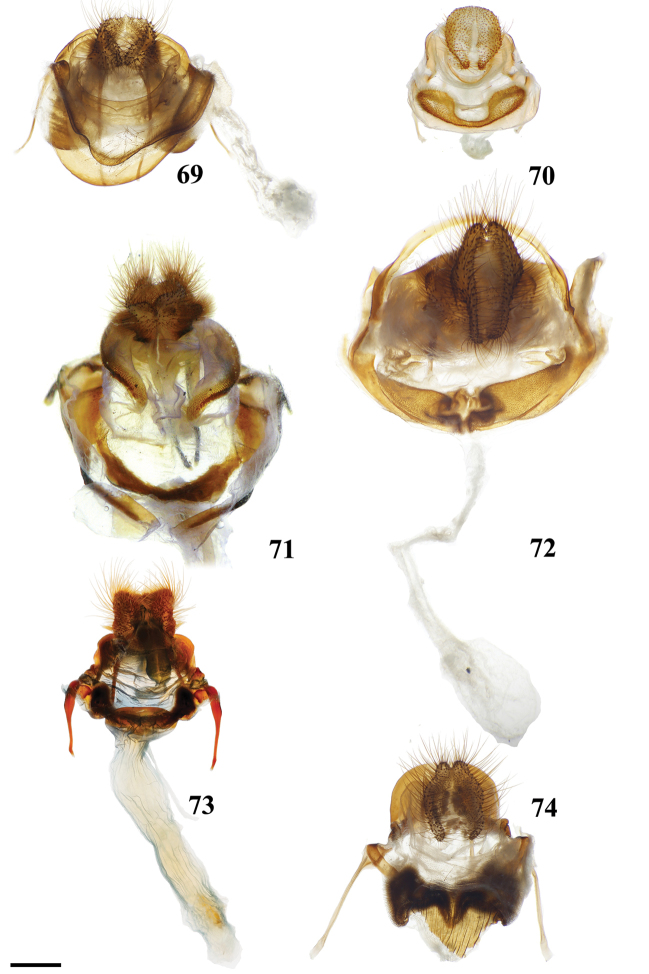
Ventral view of female genitalia of type species (except where noted) of genera belonging to Cicinninae: Cicinnini. **69***Aceclostriamus*, St Laurent diss.: 3-31-18:8 (CRAS) **70***Aleydaaccipiter*, St Laurent diss.: 4-24-17:1 [reused with permission from St Laurent et al. (2018b), SHILAP) (CUIC) **71**Cicinnuscforthane, C. Mielke diss. 34365, elongated ductus and corpus bursae not figured [reused with permission St Laurent et al. (2018c), Zootaxa] (CGCM) **72***Euphanetaromani*, St Laurent diss.: 3-31-18:9, not the type species of *Euphaneta* (MGCL) **73***Isoscellaventana*, St Laurent diss.: 3-7-16:1 [reused with permission from St Laurent and Carvalho (2017b), Journal of the Lepidopterists’ Society] (AMNH) **74***Roelmanamaloba*, St Laurent diss.: 3-31-18:10, corpus bursae not figured (CRAS). Scale bar: 1 mm.

**Figures 75–84.**
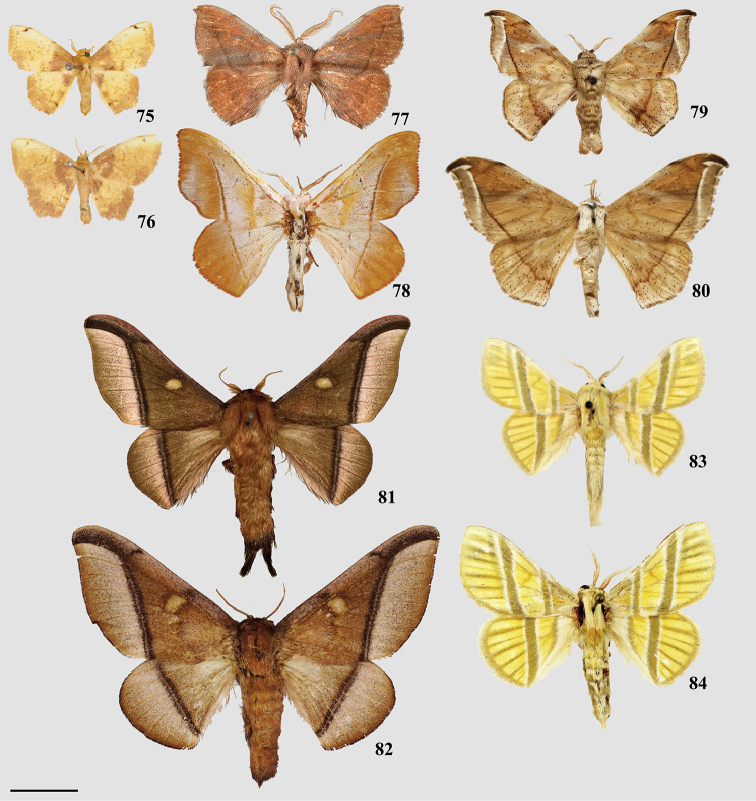
Adult specimens of type species of genera belonging to Zaphantinae, *incertae sedis*, and Aurorianinae. **75***Zaphantainfantilis*, male [photo A Giusti] (NHMUK) **76***Z.infantilis*, female [photo A Giusti] (NHMUK) **77***Cunicumaraanae*, paratype male [reused with permission St Laurent (2016), ZooKeys] (CDH) **78***Aurorianacolombiana* [reused with permission St Laurent and Mielke (2016), ZooKeys] (NHMUK) **79***Menevialantona*, male [reused with permission St Laurent and Dombroskie (2016), ZooKeys] (CUIC) **80***M.lantona*, female [adapted from St Laurent and Dombroskie (2016), ZooKeys; repaired in CS4] (USNM) **81***Roelofaolivia*, male (MWM) **82***R.olivia*, female [photo A Giusti] (NHMUK) **83***Tolypidaamaryllis*, male (MWM) **84***T.amaryllis*, female (MWM). Scale bar: 1 cm.

**Figures 85–92.**
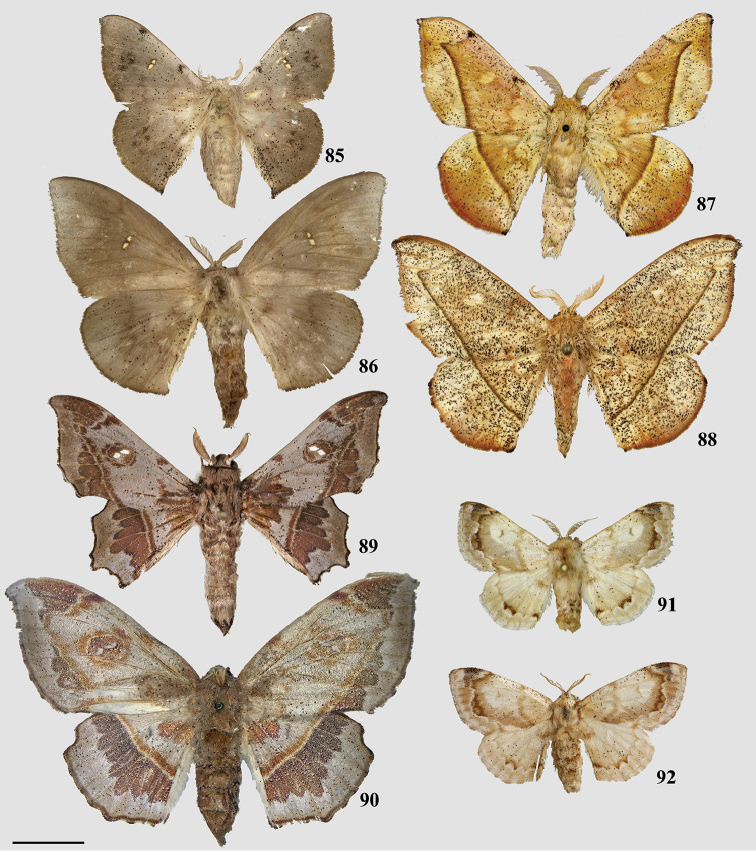
Adult specimens of type species of genera belonging to Mimalloninae. **85***Eadmunaesperans*, male (MWM) **86***E.esperans*, female (MWM) **87**Macessogacffabia [photo S Naumann] (ISEZ) **88**M.cffabia, female (NHMUK) **89***Mimalloamilia* [photo R Lahousse] (MNHN) **90***M.amilia*, female [reused with permission St Laurent and Cock (2017), Zootaxa] (MJWC) **91***Tostalloalbescens*, male [reused with permission from St Laurent and Mielke (2016), ZooKeys] (CGCM) **92***Tostalloalbescens*, female [reused with permission from St Laurent and Mielke (2016), ZooKeys] (NHMUK). Scale bar: 1 cm.

**Figures 93–103.**
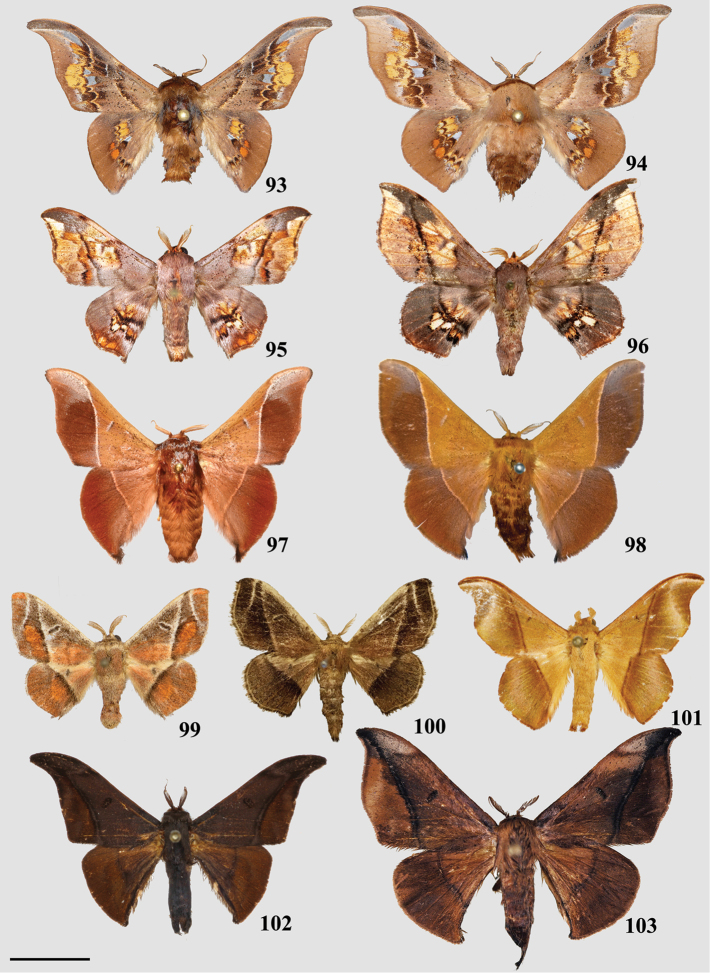
Adult specimens of type species of genera belonging to Lacosominae: Alheitini. **93***Adalgisacroesa*, male (CGCM) **94***A.croesa*, female (CGCM) **95***Arianulahaxairei*, male [photo R Lahousse] (MNHN) **96***A.haxairei*, female [photo D Herbin] (CDH) **97***Herbinallacaudina*, male [reused with permission from St Laurent et al. (2018a), Systematic Entomology] (CGD) **98***H.caudina*, female [reused with permission from St Laurent et al. (2018a), Systematic Entomology] (MJWC) **99***Taremarivara*, male [photo A Prozorov] (MWM) **100***T.rivara*, female (USNM) **101***Alheitaanoca* [reused with permission from St Laurent et al. (2018a), Systematic Entomology] (NHMUK) **102***Thaelialinamariae*, paratype male [photo R Lahousse] (MNHN) **103***T.linamariae*, female [photo R Lahousse] (MNHN). Scale bar: 1 cm.

**Figures 104, 105.**
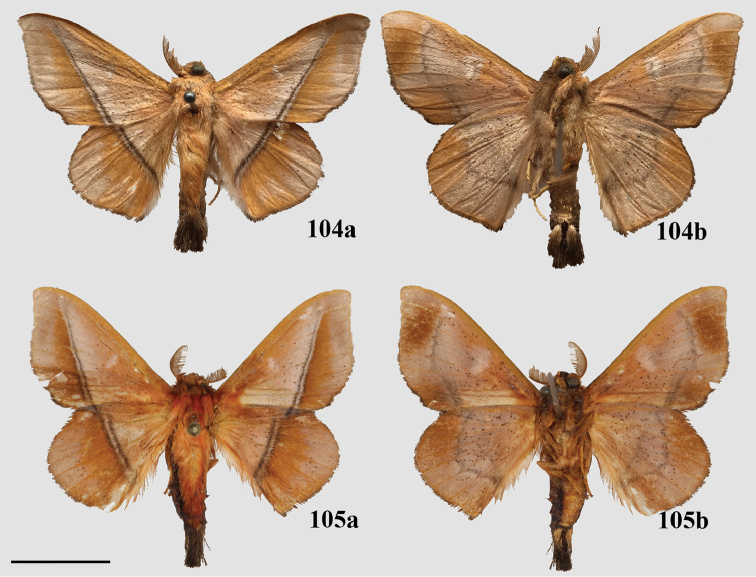
Adult male specimens of *Fatellallafatella***a** dorsal **b** ventral. **104** Holotype, French Guiana, St. Jean du Maroni (USNM) **105** French Guiana, Piste de Quesnel, km 7 [photo A Giusti] (NHMUK). Scale bar: 1 cm.

**Figure 106.**
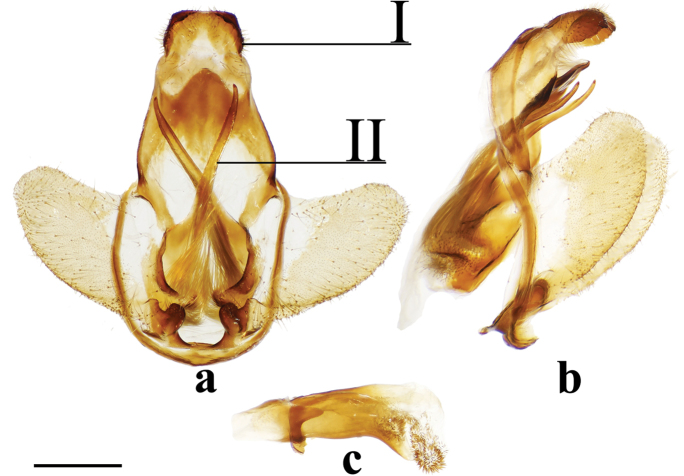
Male genitalia of *Fatellallafatella***a** ventral **b** lateral **c** phallus. Roman numerals refer to characters discussed in the text. Ecuador, Napo, Yasuni, St Laurent diss.: 10-26-17:1 (CPL). Scale bar: 1 mm.

**Figures 107–114.**
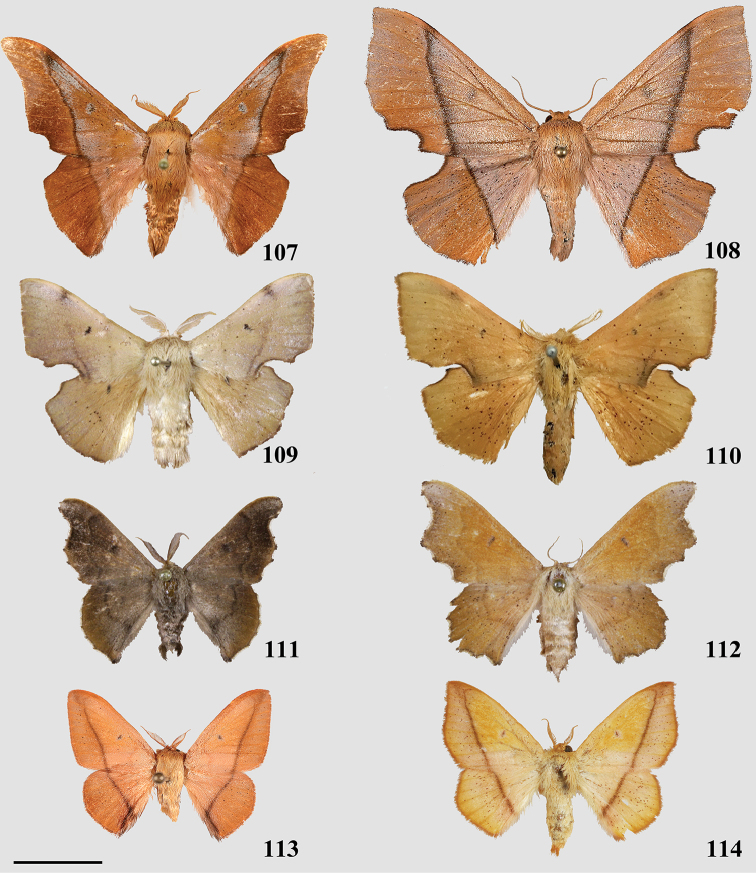
Adult specimens of type species of genera belonging to Lacosominae: Trogopterini and Lacosomini. **107***Reinmaraenthona*, male [reused with permission from St Laurent et al. (2017c), ZooKeys] (CDH) **108***R.enthona*, female [reused with permission from St Laurent et al. (2017c), ZooKeys] (CDH) **109**Trogopteracfnotata, male (MGCL) **110***Trogoptera* sp., female (NHMUK) **111***Lacosomachiridota*, male (MGCL) **112***L.chiridota*, female (MGCL) **113***Vanengamera*, male [reused with permission from St Laurent and Herbin (2017), ZooKeys] (CDH) **114***V.mera*, female [reused with permission from St Laurent and Herbin (2017), ZooKeys] (CUIC). Scale bar: 1 cm.

**Figures 115–117.**
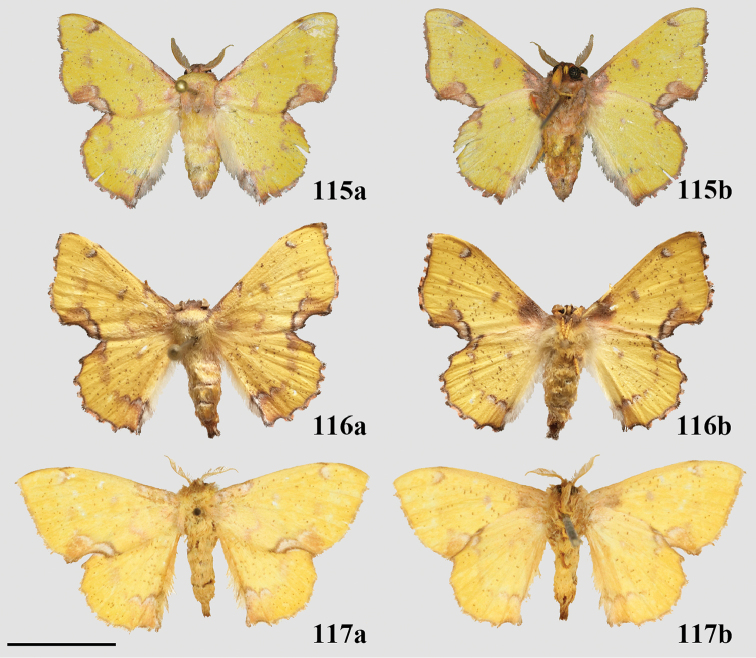
Adult specimens of *Citrallarumina***a** dorsal **b** ventral. **115** Male, Panama, Colón, Rio Indio Lodge (MGCL) **116** Female, Costa Rica, Guanacaste, Cafetal, 280 m, 90-SRNP-1981 (USNM) **117** Female, holotype, Panama, Volcán de Chiriquí, 2000–3000 ft (NHMUK). Scale bar: 1 cm.

**Figures 118, 119.**
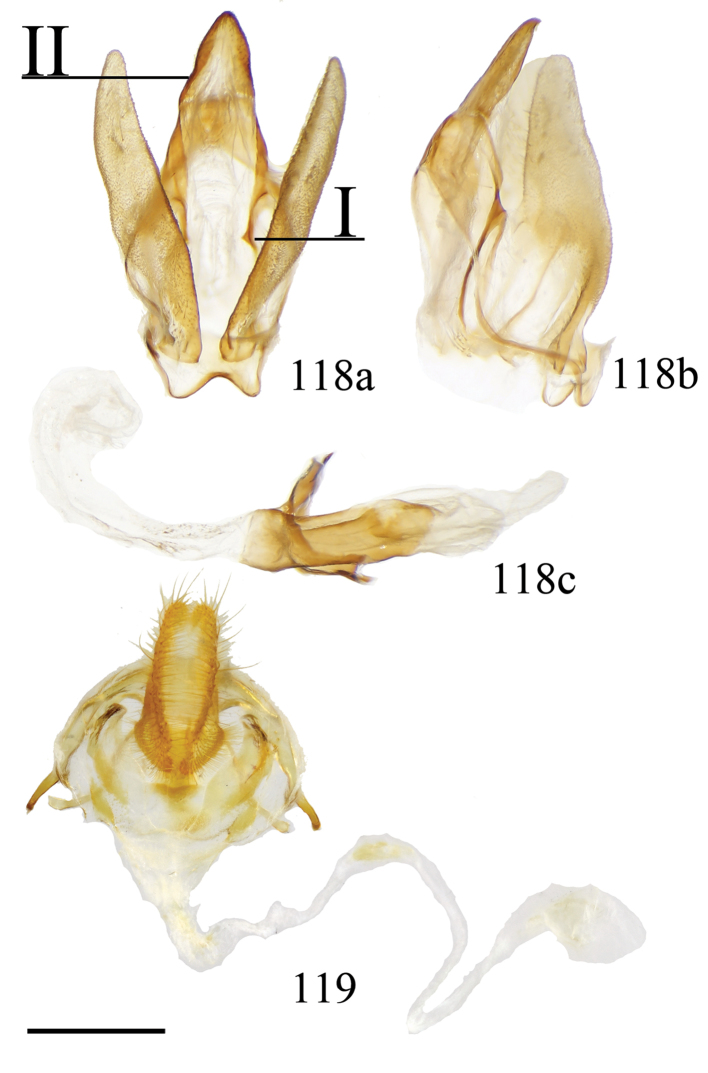
Genitalia of *Citrallarumina***a** ventral **b** lateral **c** phallus. Roman numerals refer to characters discussed in the text. **118** Male, Costa Rica, Heredia, Chilamate, Finca Selva Verde, St Laurent diss.: 9-20-17:4 (MGCL) **119** Female [ventral], holotype, Panama, Volcan de Chiriquí, 2000-3000 ft, genitalia vial NHMUK 010402303 (NHMUK). Scale bar: 1 cm.

**Figure 120.**
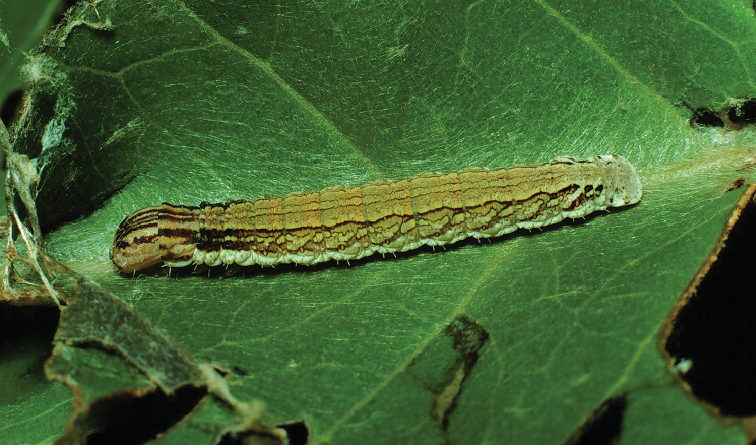
Larva of *Citrallarumina*, Costa Rica, Guanacaste, Santa Rosa, Cafetal, on *Eugeniasalamensis* (Myrtaceae), voucher ID: 90-SRNP-1981 [resulting adult female specimen in Fig. 116]. Photo courtesy of D Janzen, used with permission.

**Figures 121–130.**
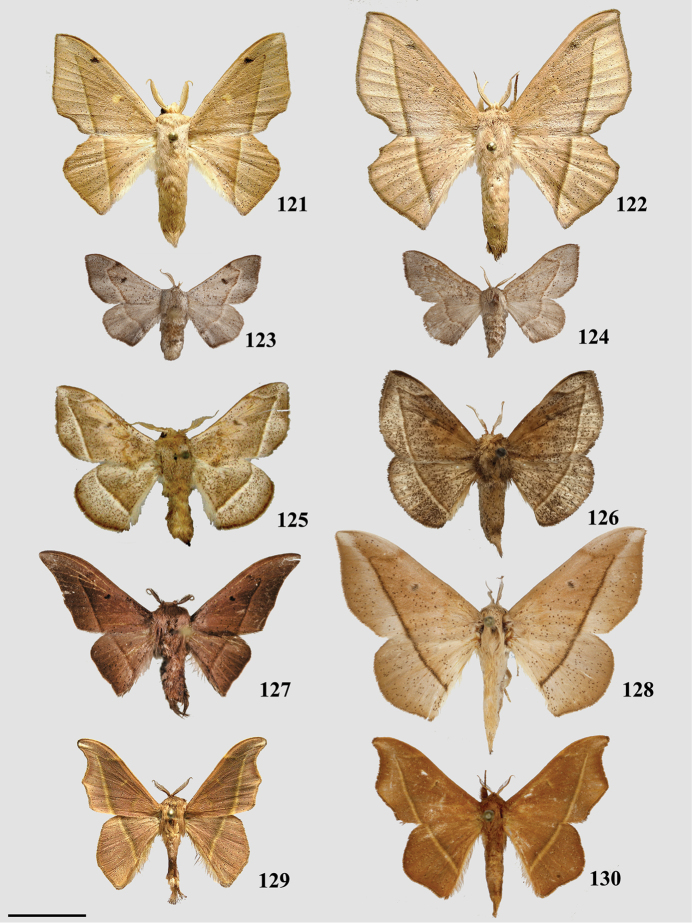
Adult specimens of type species of genera belonging to Druenticinae: Druenticini. **121***Druenticapartha*, male [reused with permission from St Laurent et al. (2018a), Systematic Entomology] (MNHN) **122***D.partha*, female [photo P Collet] (MNHN) **123***Micrallominutus*, holotype male [reused with permission from St Laurent and Mielke (2016), ZooKeys] (DZUP) **124***M.minutus*, paratype female [reused with permission from St Laurent and Mielke (2016), ZooKeys] (USNM) **125***Pameaalbistriga*, syntype male [photo A Giusti] (NHMUK) **126**P.cfalbistriga, female (CUIC) **127***Procinnuscahureli*, holotype male [photo R Lahousse] (MNHN) **128***P.cahureli*, female [photo A Giusti] (NHMUK) **129***Ulalumavalva*, male [reused with permission from St Laurent and Cock (2017), Zootaxa] (MNHN) **130***U.valva*, female [reused with permission from St Laurent et al. (2018a), Systematic Entomology] (NHMUK). Scale bar: 1 cm.

**Figure 131.**
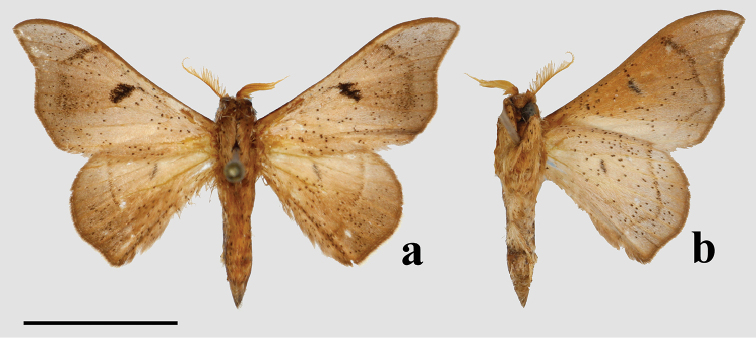
Adult male specimen of *Lepismallamontagnaniae***a** dorsal **b** ventral. French Guiana, Piste Bélizon, km 27 [photo A Giusti] (NHMUK). Scale bar: 1 cm.

**Figure 132.**
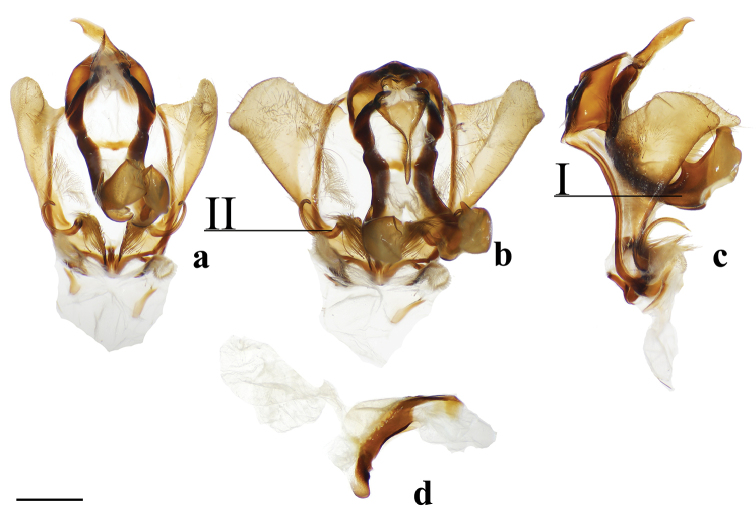
Male genitalia of *Lepismallamontagnaniae***a** ventral with valvae in natural position **b** ventral with valvae spread **c** ateral **d** phallus. Roman numerals refer to characters discussed in the text. Note that uncus is twisted to the left in Fig. 132a. French Guiana, genitalia vial NHMUK 010402310 (NHMUK). Scale bar: 1 mm.

**Figures 133–137.**
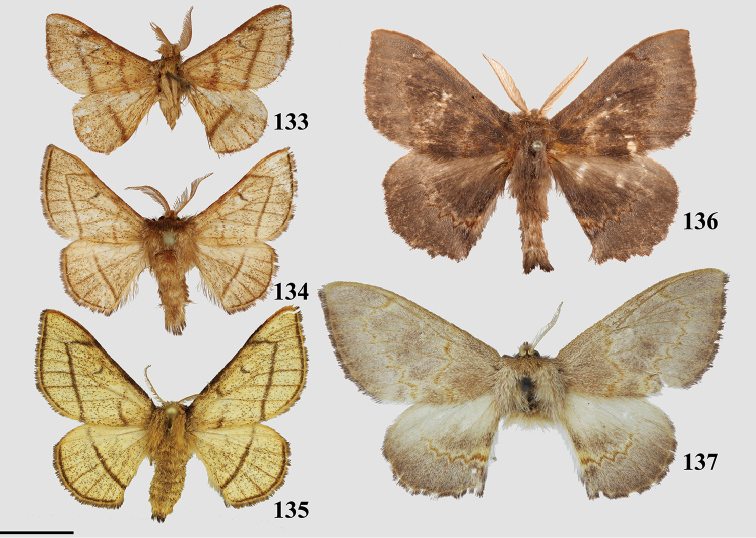
Adult specimens of type species (except where noted) of genera belonging to Druenticinae: Luramini, note that two species of *Lurama* are figured to better illustrate this genus. **133***Luramapenia*, male [reused with permission from St Laurent (2016), ZooKeys] (USNM) **134***L.quindiuna*, male, not type species of *Lurama* [reused with permission from St Laurent (2016), ZooKeys] (MWM) **135***L.quindiuna*, female, not type species of *Lurama* [reused with permission from St Laurent (2016), ZooKeys] (MWM) **136***Ulmararotunda*, male [photo T Malm] (NHRS) **137***U.rotunda*, [reused with permission from St Laurent (2016), ZooKeys] (MNHU). Scale bar: 1 cm.

**Figures 138–141.**
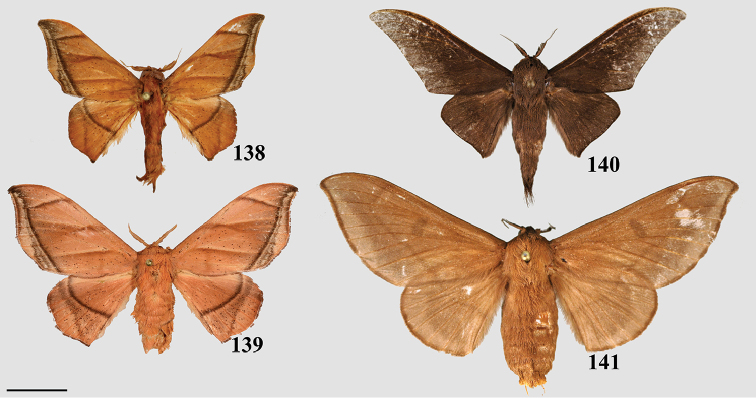
Adult specimens of type species of genera belonging to Cicinninae: Psychocampini. **138***Biterolfaalthea*, male [reused with permission from St Laurent et al. (2017d), Tropical Lepidoptera Research] (NHMUK) **139***Biterolfaalthea*, female [reused with permission from St Laurent et al. (2017d), Tropical Lepidoptera Research] (CDH) **140***Psychocampaconcolor*, male [photo R Lahousse] (MNHN) **141***P.concolor*, female [photo R Lahousse] (MNHN). Scale bar: 1 cm.

**Figures 142–145.**
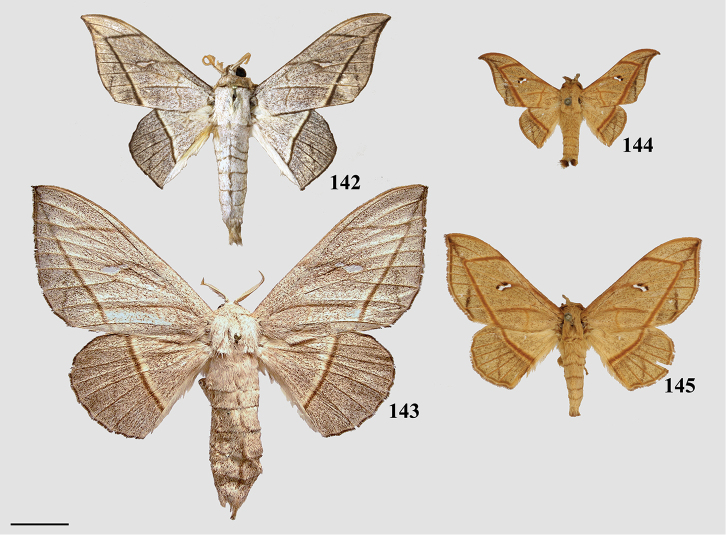
Adult specimens of type species of genera belonging to Cicinninae: Bedosiini. **142***Bedosiafraterna*, male (MGCL) **143***B.fraterna*, female [photo P Collet] (MNHN) **144***Bedosialloforbesi*, male [reused with permission from St Laurent et al. (2018a), Systematic Entomology] (NHMUK) **145***B.forbesi*, female [reused with permission from St Laurent et al. (2018a), Systematic Entomology] (NHMUK). Scale bar: 1 cm.

**Figures 146–152.**
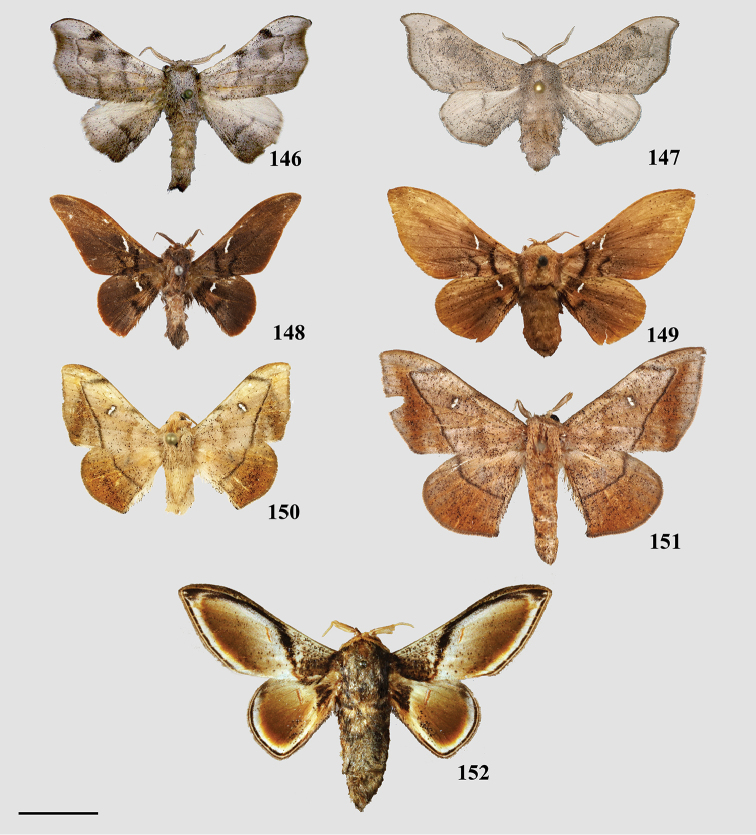
Adult specimens of type species of genera belonging to Cicinninae: Cicinnini. **146***Aceclostriamus*, male [photo S Naumann] (ZSM) **147**A.cfmus, female [photo C Mielke] (CGCM) **148***Aleydaaccipiter*, male [reused with permission from St Laurent et al. (2018a), SHILAP] (NHRS) **149***A.accipiter*, female [reused with permission from St Laurent et al. (2018b), SHILAP] (CUIC) **150**Arcinnuscfhoedli, male (MWM) **151***A.hoedli*, female [photo R Lahousse] (MNHN) **152***Euphanetadivisa*, male (MGCL). Scale bar: 1 cm.

**Figures 153–158.**
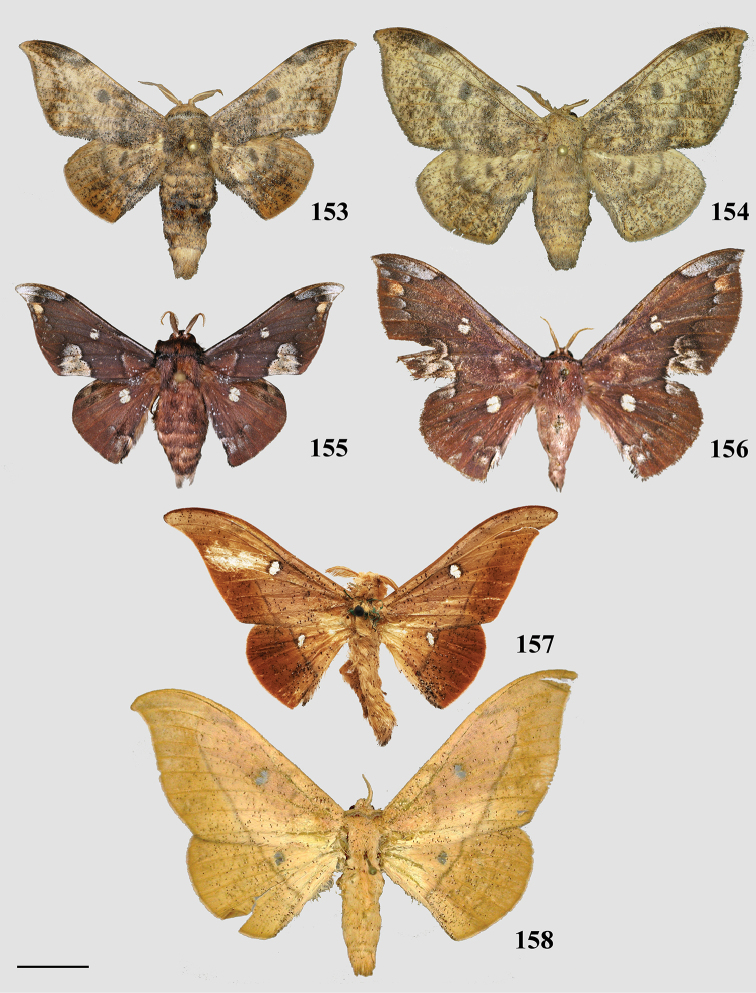
Adult specimens of type species of genera belonging to Cicinninae: Cicinnini. **153**Cicinnuscforthane, male [reused with permission St Laurent et al. (2018c), Zootaxa] (CGCM) **154**C.cforthane, female [reused with permission St Laurent et al. (2018c), Zootaxa] (CGCM) **155***Roelmanamaloba*, male [photo R Lahousse] (MNHN) **156***R.maloba*, female [photo R Lahousse] (MNHN) **157***Isoscellaventana*, male [photo T Malm] (NHRS) **158***I.ventana*, female [photo S Naumann] (ZSM). Scale bar: 1 cm.

**Figures 159–163.**
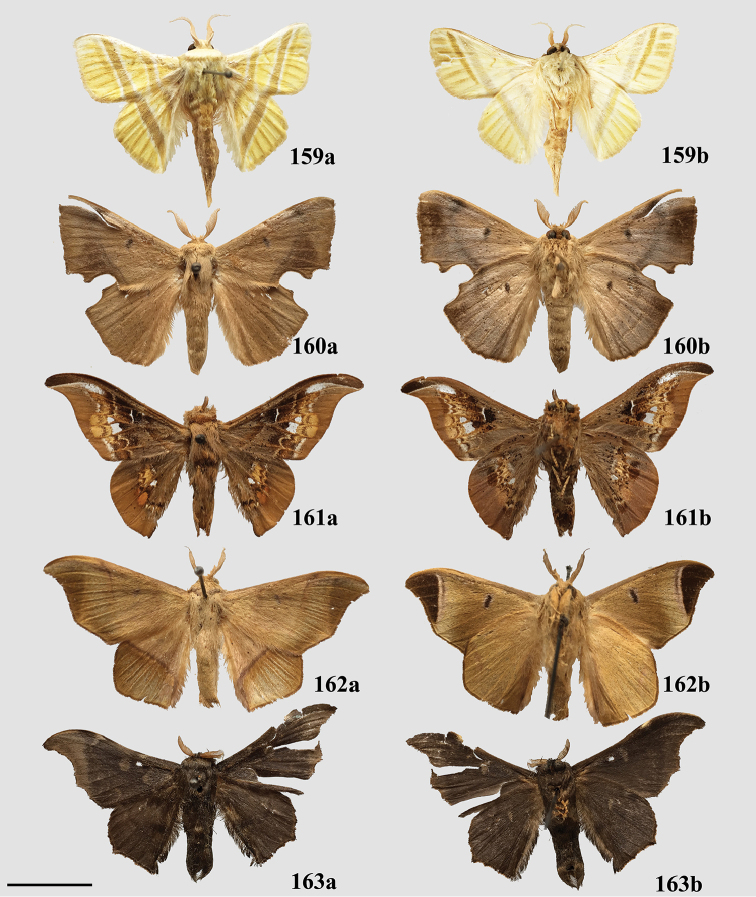
Lectotypes designated in the present work for *incertae sedis* and Lacosominae**a** dorsal **b** ventral. See annotations in Section 4 for complete label data for all lectotypes. All specimens are male **159***Hydriasamaryllis* Schaus (= *Tolypidaamaryllis*) **160***Trogopteraalthora* Schaus **161***Adalgisacroesa* Schaus **162***Perophorapulloides* Dognin (= *Alheitapulloides*) **163***Lacosomabriasia* Schaus. Scale bar: 1 cm.

**Figures 164–168.**
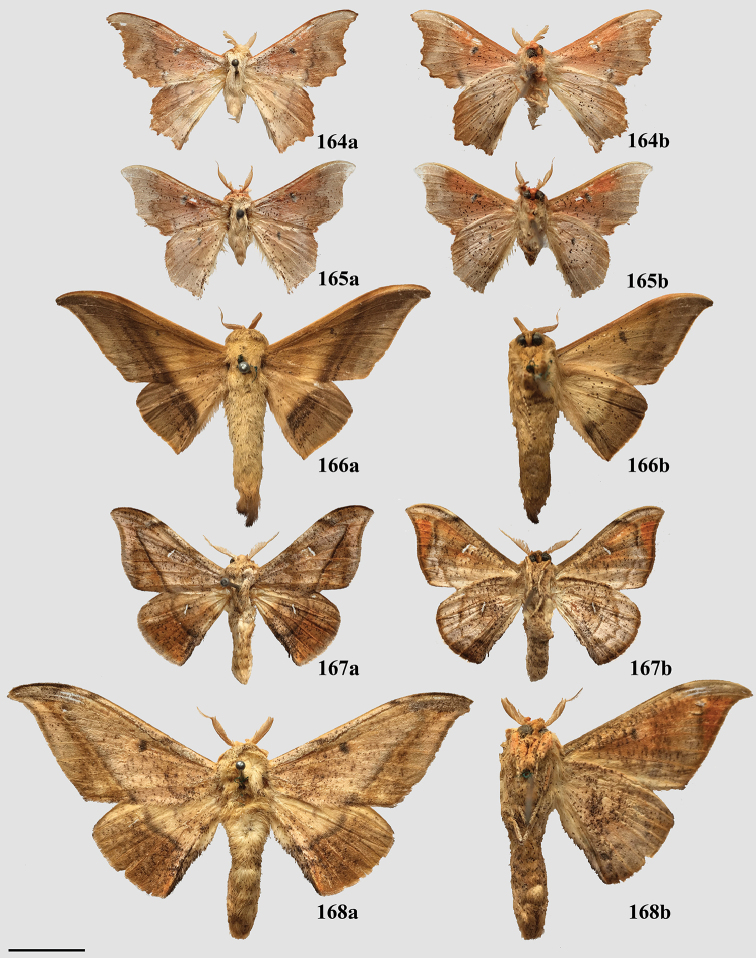
Lectotypes designated in the present work for Lacosominae and Cicinninae**a** dorsal **b** ventral. See annotations in Section 4 for complete label data for all lectotypes. All specimens are male. **164***Lacosomadiederica* Schaus **165***Lacosomaraydela* Schaus **166***Cicinnuslacuna* (= *Psychocampalacuna*) **167***Cicinnuscorallina* Dognin **168***Cicinnuslatris* Schaus. Scale bar: 1 cm.

**Figures 169–171.**
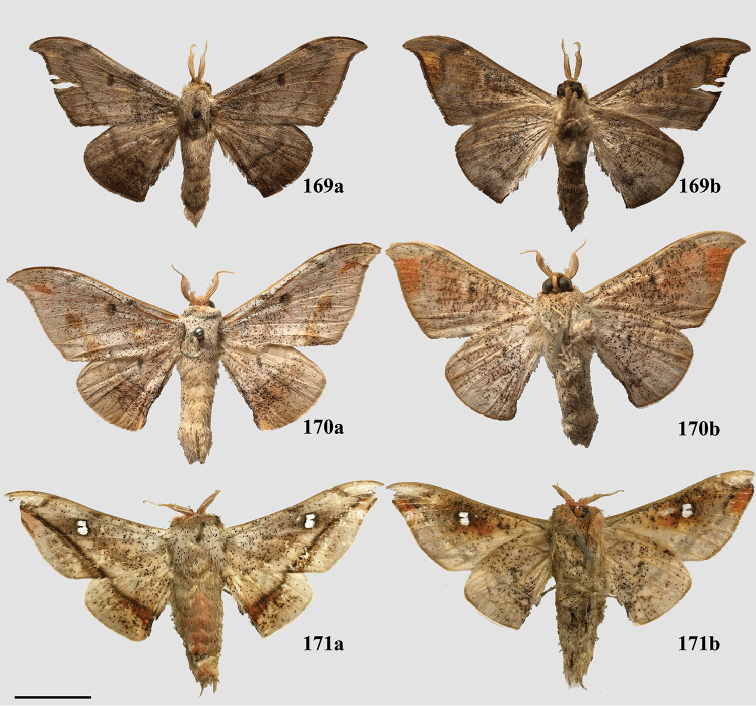
Lectotypes and neotype designated in the present work for Cicinninae**a** dorsal **b** ventral. See annotations in Section 4 for complete label data for all lectotypes and the neotype. All specimens are male. **169***Cicinnussolvens* Dyar, lectotype **170***Cicinnustuisana* Schaus, lectotype **171***Mimallodespecta* Walker (= *Cicinnusdespecta*), neotype. Scale bar: 1 cm.

## Supplementary Material

XML Treatment for Zaphanta

XML Treatment for Cunicumara

XML Treatment for Menevia

XML Treatment for Roelofa

XML Treatment for Tolypida

XML Treatment for Auroriana

XML Treatment for Eadmuna

XML Treatment for Macessoga

XML Treatment for Mimallo

XML Treatment for Tostallo

XML Treatment for Reinmara

XML Treatment for Trogoptera

XML Treatment for Adalgisa

XML Treatment for Alheita

XML Treatment for Arianula

XML Treatment for Fatellalla

XML Treatment for Herbinalla

XML Treatment for Tarema

XML Treatment for Thaelia

XML Treatment for Citralla

XML Treatment for Lacosoma

XML Treatment for Vanenga

XML Treatment for Druentica

XML Treatment for Lepismalla

XML Treatment for Micrallo

XML Treatment for Pamea

XML Treatment for Procinnus

XML Treatment for Ulaluma

XML Treatment for Lurama

XML Treatment for Ulmara

XML Treatment for Biterolfa

XML Treatment for Psychocampa

XML Treatment for Bedosia

XML Treatment for Bedosiallo

XML Treatment for Aceclostria

XML Treatment for Aleyda

XML Treatment for Arcinnus

XML Treatment for Cicinnus

XML Treatment for Euphaneta

XML Treatment for Isoscella

XML Treatment for Roelmana

## Supplementary materials

Supplementary material 1 Table 1. Morphological character matrix for Mimallonidae and two outgroups Data type: species data Explanation note: Characters 1 to 25 are male genitalia, 26 to 31 are female genitalia, and 32 to 55 are external morphological characters. See Suppl. material 2 for additional explanation of characters and their states. Unknowns are coded as “?”, inapplicable characters are coded as “-”. This is an open access article distributed under the terms of the Creative Commons Attribution License (CC BY 4.0), which permits unrestricted use, distribution, and reproduction in any medium, provided the original author and source are credited.Ryan A. St Laurent, Akito Y. Kawahara

Supplementary material 2 Explanation of the 55 characters and their states that were coded for the present study Data type: species data This is an open access article distributed under the terms of the Creative Commons Attribution License (CC BY 4.0), which permits unrestricted use, distribution, and reproduction in any medium, provided the original author and source are credited.Ryan A. St Laurent, Akito Y. Kawahara

Supplementary material 3 Figures denoting characters coded in Suppl. material 1: Table 1. Nine plates total, seven plates of genitalia, two plates of external morphology Data type: species data This is an open access article distributed under the terms of the Creative Commons Attribution License (CC BY 4.0), which permits unrestricted use, distribution, and reproduction in any medium, provided the original author and source are credited.Ryan A. St Laurent, Akito Y. Kawahara

Supplementary material 4 Constraint tree in Newick format, utilized in our constrained ML analysis Data type: phylogenetic data Explanation note: The constraint tree was derived from the dataset2_PART2 analysis of St Laurent et al. (2018a). This is an open access article distributed under the terms of the Creative Commons Attribution License (CC BY 4.0), which permits unrestricted use, distribution, and reproduction in any medium, provided the original author and source are credited.Ryan A. St Laurent, Akito Y. Kawahara

Supplementary material 5 Unconstrained morphological ML analysis conducted in IQ-TREE Data type: phylogenetic data Explanation note: Support values refer to SH-aLRT/UFBoot. This is an open access article distributed under the terms of the Creative Commons Attribution License (CC BY 4.0), which permits unrestricted use, distribution, and reproduction in any medium, provided the original author and source are credited.Ryan A. St Laurent, Akito Y. Kawahara

Supplementary material 6 Constrained morphological ML analysis conducted in IQ-TREE Data type: phylogenetic data Explanation note: Support values refer to SH-aLRT/UFBoot. Constraint used can be found in Suppl. material 4. This is an open access article distributed under the terms of the Creative Commons Attribution License (CC BY 4.0), which permits unrestricted use, distribution, and reproduction in any medium, provided the original author and source are credited.Ryan A. St Laurent, Akito Y. Kawahara

Supplementary material 7 Majority rule consensus tree of the five most parsimonious trees inferred in TNT Data type: phylogenetic data Explanation note: Support values refer to 100 jackknife replicates. This is an open access article distributed under the terms of the Creative Commons Attribution License (CC BY 4.0), which permits unrestricted use, distribution, and reproduction in any medium, provided the original author and source are credited.Ryan A. St Laurent, Akito Y. Kawahara
