# Supplementary material for: Reclassification of the Sack-bearer Moths (Lepidoptera, Mimallonoidea, Mimallonidae)
Source: Zookeys. 2019 Jan 10;(815):1–114. doi: 10.3897/zookeys.815.27335 (PMC6336762; doi:10.3897/zookeys.815.27335)
Supplement: Supplementary material 2 [file zookeys-815-001-s002.pdf]

Supplementary File 2: Document 1. Explanation of characters and their character states. See Supplementary File 3 for figures of characters and their states.

Male genitalia

1. Uncus
0: Uncus deeply bifurcated
1: Uncus somewhat rounded, with pair of small teeth/spines
2: Uncus triangular basally, apically pointed/rounded, notably narrowed for more than third of length distally (may be curved viewed laterally)
3: Uncus triangular basally, apically pointed/rounded, without apical narrowing
4: Uncus bell-shaped basally with elongated fingerlike distal process (viewed ventrally)
5: Uncus thin, spine-like
6: Uncus truncated, rectangular
7: Uncus truncated but basally triangular

2. Tegumen
0: Uncus proximal margin clearly differentiated from tegumen
1: Uncus proximal margin continuous with tegumen

3: Gnathos presence
0: Gnathos absent
1: Gnathos present

4. Gnathos fusion
0: Gnathos unfused mesally
1: Gnathos fused mesally

5. Gnathos configuration (projections/ arms)
0: Gnathos lacking mesal projection(s)
1: Gnathos with singular central protuberance/projection, not flattened
2: Gnathos with paired projections, narrow, fingerlike (same thickness along length for entire projection)
3: Gnathos thin, plate-like
4: Gnathos extensions elongate, arm-like, often extending to saccular valva margin
5: Gnathos extensions flattened, triangular, not arm-like
6: Gnathos extensions robust, boxing glove-like in shape
7: Gnathos extensions pair of bifurcated projections (two separate bifurcating structures, four points total)
8: Gnathos extensions broadly triangular, heavily sclerotized (not flattened)
9: Gnathos extensions pair of thick protuberances held close together mesally
A: Gnathos synscleritous or attached with membrane to juxta, extensions/projections variable
B: Gnathos extensions thin, flattened distally, usually with sclerotization weaker distally
C: Gnathos extensions robust, arm-like, with significant membranous component
D: Gnathos thin, plate-like with thicker, more heavily sclerotized mesal projection

6. Gnathos base

0: Base of gnathos shape not easily defined (often due to being covered by mesal area of a fused gnathos or obscured by huge projections, or simply due to lack of fusion)
1: Base of gnathos circular or ovoid, heavily sclerotized
2: Base of gnathos rectangular
3: Base of gnathos somewhat irregular due to origination of gnathos dorsally on vinculum below uncus such that gnathos margins loop downward

7. Subuncus projections (in addition to gnathos)

0: Subuncus projections absent
1: Subuncus projections present

8. Valvae tusks

0: Absent
1: Elongated, usually more than half length of valvae
2: Short, not much longer than half valva length

9. Vinculum tusks

0: Absent
1: Sharp, one tusk or single pair
2: Blunt, one tusk or single pair
3: Paired, two separate tusk pairs

10. Transtilla/mesal diaphragmal sclerotized plate

0: Absent
1: Triangular
2: Band-like
3: Rectangular, plate-like
4: Knob-like

11. Valva apodemes/transtilla modifications

0: Valvae apopdemes/transtilla absent
1: Valvae apopdemes/transtilla extensions lobe-like covered in setae
2: Valvae apopdemes/transtilla extensions internally wrinkled triangles/lobes
3: Valvae apopdemes/transtilla extensions elongated, filamentous
4: Valvae apopdemes/transtilla extensions columnar, broad, spanning near entire dorsal-ventral height of vinculum
5: Valvae apopdemes/transtilla extensions spined (multiple)
6: Valvae apopdemes/transtilla projections blade-like
7: Valva apodeme an upward projected lobe, sometimes covered in setae/spines
8: Transtilla/valva apodeme/vinculum complex situated back away from vinculum into body cavity, this complex with very elongated, pair of curved tusk-like arms that extend out through center of vinculum over phallus (may also have secondary lobes from base of valvae)
9: Valva apodeme as elongated singular tusk-like spines

A: Valvae apodeme/transtilla projections clubbed

12. Sternite VIII modified by heavily sclerotized structures
0: Sternite VIII unmodified, no sclerotized extensions
1: Sternite VIII modified – usually with bifid projections, arms, or Bilobed section

13. Diaphragm
0: Diaphragm without setae
1: Diaphragmal setae in four discrete sacks
2: Diaphragmal setae not in discrete sacks but present

14. Juxta
0: Juxta forming sharp-tipped, hammerhead like extension well above and beyond mostly membranous phallus
1: Juxta limited to connecting point from base of phallus to vinculum
2: Juxta with dorsal projections protuberances/projections (usually paired), but not overly complex such that phallus cannot be excised
3: Juxta/vinculum/diaphragm components complicated otherwise highly sclerotized surrounding phallus with various lateral and/or dorsal projections from phallus, phallus generally difficult to remove due to various complicated connections with juxta and diaphragm
4: Juxta as broad, ridged protuberance fused to phallus
5: Juxta as hood-like process fused to phallus
6: Juxta as pair of knobs on either side of phallus, connecting phallus to vinculum
7: Juxta fused to narrow sclerotizations from base of valvae which swoop forward to meet laterally on either side of phallus

15. Sacculus
0: Sacculus not distinctly thickened/ or with any modified spines/protuberances
1: Sacculus with erect sharp spine/ small curled projection
2: Sacculus with triangular projection (may be situated mesally)
3: Sacculus with triangular projection in addition to mesal valva projection
4: Sacculus with substantial thickening due to curl (which too may be spined)
5: Sacculus with trumpet shaped extension
6: Sacculus with lump
7: Sacculus with substantial thickening, but not curled, often with small clump of spines
8: Sacculus with blunt fingerlike or knob-like projection at base of valvae
9: Sacculus with small projection near to apex of valvae than base
A: Sacculus curled, but not substantially thickened
B: Sacculus broadened near base of valvae/vinculum, densely covered in setae
C: Sacculus more heavily sclerotized and extending beyond ventral edge of valvae
D: Sacculus with indentation

16. Dorsal phallic membrane
0: Absent
1: Present

17. Valvae shape
0: Distinctly bent (elbowed)
1: Valvae triangular or distally narrowed and lobe-like
2: Valvae rectangular, or parallelogram shaped
3: Valvae elongated, narrow, paddle-like, narrower than long
4: Valvae rounded, wider than long/ width roughly equal to length
5: Valvae irregular, mostly sclerotized
6: Valvae irregular, mostly membranous
7: Valvae upwardly curved/angled/perpendicular to vinculum, usually extending from upper half of vinculum such that base of valvae does not span from base of tegumen to base of vinculum

18. Phallus orientation asymmetrical
0: Phallus oriented symmetrically (extending from center of genitalia, outward)
1: Phallus oriented asymmetrically (extending from left side of genitalia, outward when viewed ventrally)

19. Vinculum apodemes
0: Absent
1: Present

20. Phallus shape
0: Phallus snake-like
1: Phallus sharp, sword-like
2: Phallus generally cylindrical/simple overall (terminus variously shaped)
3: Phallus distally flattened; distally with sclerotization restricted to margin such that terminal half of phallus appearing "open" from dorsal or ventral perspective, and spade-like in shape due to outline of sclerotized margin or not
4: Phallus tubular, very narrow
5: Phallus wide (somewhat flattened) curved

21. Vesica
0: Vesica simple
1: Vesica scobinate (many minute sclerotizations)
2: Vesica covered in thick setae

22. Cornutus/cornuti
0: Absent
1: Present

23. Coecum phallus
0: Coecum phallus absent
1: Coecum phallus present, simple
2: Coecum phallus present, bifurcated

24. Phallus projections (generally more heavily sclerotized than remainder of genitalia)

0: Absent
1: Phallus with apical ventral (downward) projection
2: Phallus with pair of elongated spine-like processes emanating somewhat laterally
3: Dorsal protuberance/spine/crest
4: Lateral spines
5: Distal tip scoop-like
6: Distal tip with spine

25. Saccus
0: Absent
1: Present as solid anteriorad facing lobe (typical saccus)
2: Present as upward angled bridge-like sclerotization spanning base of vinculum, often with extended thinly sclerotized sheet-like lip

Female genitalia

26. Apophyses anteriores
0: Absent
1: Present

27. Apophyses posteriores
0: Absent
1: Present

28. Apophyses posteriores length
0: > apophyses anteriores
1: < apophyses anteriores
2: = apophyses anteriores

29. Lamella antevaginalis
0: Mesally fused by sclerotization
1: Mesally unfused/ membranous

30. Ductus and corpus bursae
0: Tiny (smaller than papillae anales), baglike/balloon-like
1: Elongated for entire length, mostly continuous in width with ductus bursae
2: Broad/large (no clear ductus bursae)
3: Narrow ductus bursae with balloon-like corpus bursae
4: Ductus bursae broader than corpus bursae
5: Corpus bursae broad with sclerotized ductus bursae

31. Presence of setae on tergite VIII
0: Setae absent
1: Setae coverage entire or patchy, but not clumped to either side of ostium bursae
2: Setae clumped in nodes on either side of ostium bursae

External morphology

32. Female antennae
0: Female antennae dentate/filiform
1: Antennae rami continuously shortened
2: Antennae rami abruptly shorted

33. Male antennae
0: Antennae filiform
1: Male pectination continuously shortened along length
2: Male pectination abruptly shortened distally

34. Frenulum (coded for male only)
0: Absent/reduced to multiple small bristles (i.e. no retinaculum or not generally visible without removing wings)
1: Present/obvious as single bristle, usually with retinaculum

35. FW dorsal postmedial line
0: Absent
1: Poorly defined
2: Preapical, band-like
3: Preapical, fine to slightly wavy, angled or notably curved toward, and reaching costa apically (not including any continuation toward apex after angle toward costa)
4: Preapical, fine, not angled toward, and reaching costa apically
5: Fine, fully apical (not angled toward costa)
6: Serrate/ crenulate (preapical)

36. FW dorsal antemedial line (any wing)
0: Absent
1: Present (often very faint)

37. HW dorsal postmedial line
0: Absent
1: Present

38. HW dorsal antemedial line
0: Absent
1: Present (often very faint)

39. FW ventral postmedial line
0: Absent or nearly so (consisting of several discontinuous spots)
1: Continuous, not dentate (straight/ slightly wavy/ smoothly convex)
2: Dentate
3: Outwardly lobed (not spiked) mesally (often only apical portion apparent)
4: Mesally incomplete/ broken by spikes at intersection with veins M3 and CuA1

40. HW ventral postmedial line
0: Absent or nearly so
1: Not dentate (straight/ slightly wavy/ smoothly convex)
2: Dentate
3: Outwardly lobed (not spiked) mesally (often only apical portion apparent)
4: Mesally incomplete/ broken by spikes at intersection with veins mesally

41. Ventral antemedial line (any wing)
0: Absent
1: Present

42. FW hyaline patches
0: Absent
1: Present as B, BB-shape, or oval bisected by M2
2: Crescent shaped
3: Present in some other configuration

43. HW hyaline patches
0: Absent
1: Present as B-shape bisected by M2
2: Crescent shaped
3: Present in some other configuration

44. Costal mark
0: Absent
1: Present

45. FW dorsal discal mark
0: Absent
1: Present

46. HW dorsal discal mark
0: Absent
1: Present

47. FW ventral discal mark
0: Absent
1: Present

48. HW ventral discal mark
0: Absent
1: Present

49. Wing margin
0: Smooth (sometimes with mesal outward point)

1: Crenulate (usually with points at each vein intersection with margin)
2: Smooth with tornal notch (FW and HW)

50. Wing apex
0: Concave (even if slight) below apex/ deeply falcate
1: Not concave below apex, convex or flat

51. Postmedial lunule/tornal line
0: Absent
1: Well-defined lunule (white/gray)
2: Diffuse (brown, black, dark gray)
3: Tornal line

52. Apical streak
0: Absent
1: Present

53. Ventral abdominal line
0: Absent
1: Present

54. Paired distal abdominal tufts (male only)
0: Absent
1: Present

55. FW Ventrum apical patch
0: Absent
1: Present, brown/black heavy contrast against otherwise mostly plain ventrum, often outlined by lunule
2: Present, orange to blood red
