# Supplementary material for: Reclassification of the Sack-bearer Moths (Lepidoptera, Mimallonoidea, Mimallonidae)
Source: Zookeys. 2019 Jan 10;(815):1–114. doi: 10.3897/zookeys.815.27335 (PMC6336762; doi:10.3897/zookeys.815.27335)
Supplement: Supplementary material 3 [file zookeys-815-001-s003.pdf]

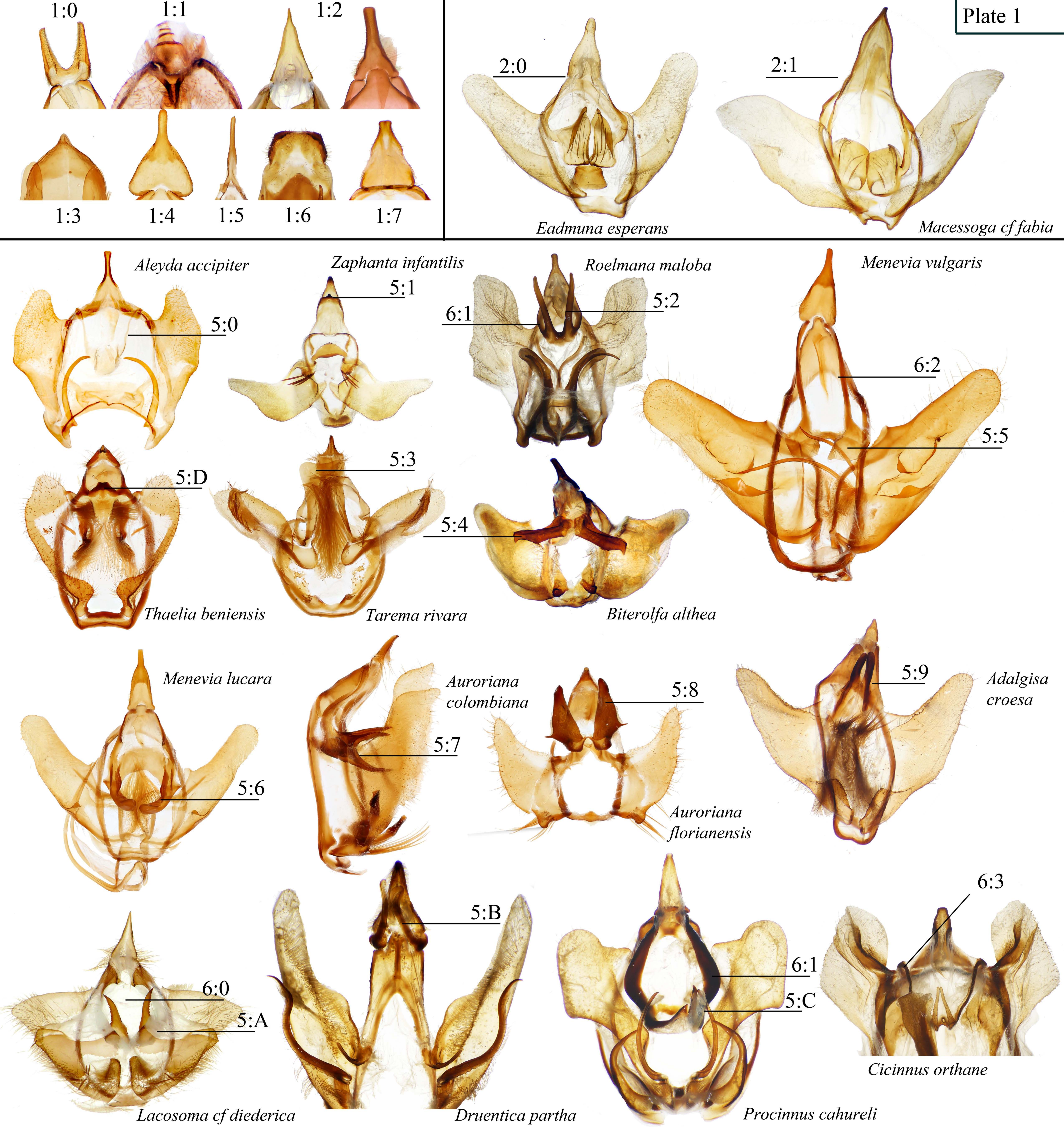
Plate 1
1:0
1:1
1:2
1:3
1:4
1:5
1:6
1:7
2:0
2:1
Eadmuna esperans
Macessoga cf fabia
Aleyda accipiter
5:0
Zaphanta infantilis
5:1
Roelmana maloba
6:1
5:2
Menevia vulgaris
6:2
5:5
5:D
Thaelia beniensis
5:3
5:4
Tarema rivara
Biterolfa althea
Menevia lucara
5:6
Auroriana colombiana
5:7
5:8
Auroriana florianensis
5:9
Adalgisa croesa
6:0
5:A
Lacosoma cf diederica
5:B
Druentica partha
6:1
5:C
Procinnus cahureli
6:3
Cicinnus orthane

Plate 2

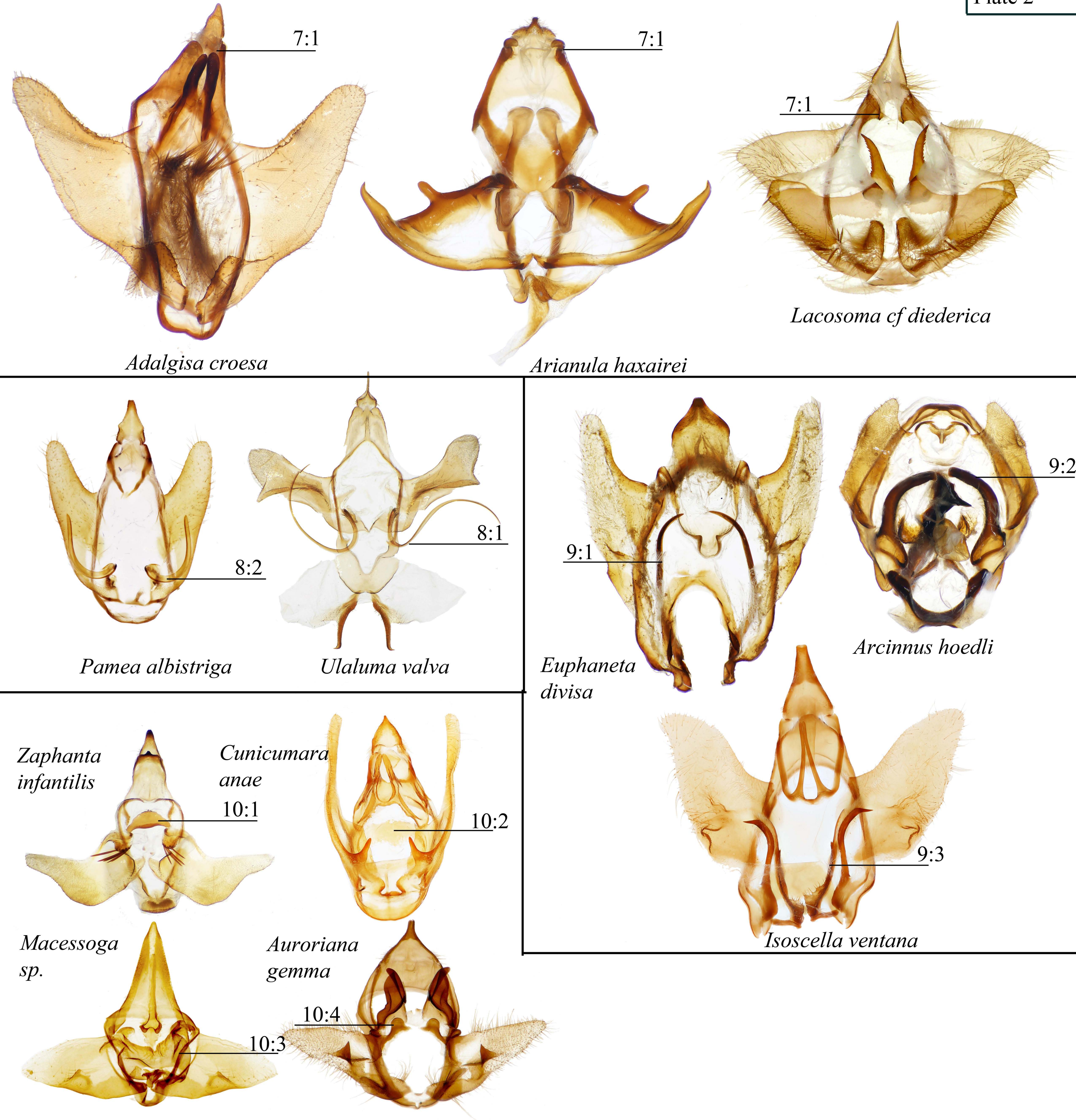

7:1
7:1
7:1
Adalgisa croesa
Arianula haxairei
Lacosoma cf diederica
8:2
8:1
Pamea albistriga
Ulaluma valva
9:2
9:1
Euphaneta divisa
Arcinnus hoedli
Zaphanta infantilis
Cunicumara anae
10:1
10:2
9:3
Isoscella ventana
Macessoga sp.
Auroriana gemma
10:4
10:3

Plate 3

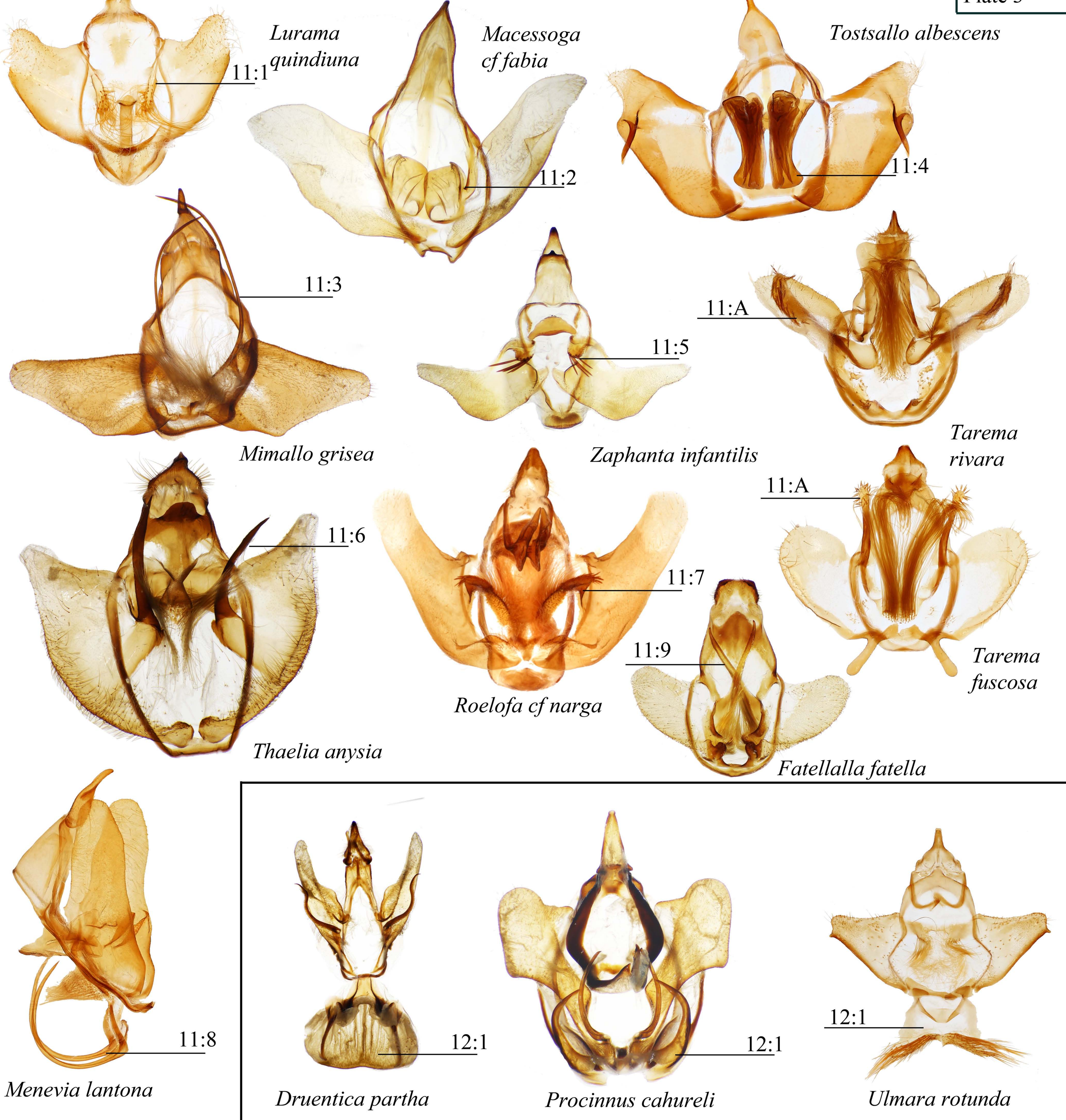
Lurama quindiuna
11:1
Macessoga cf fabia
11:2
Tostsallo albescens
11:4
11:3
Mimallo grisea
11:5
Zaphanta infantilis
11:A
Tarema rivara
11:A
11:6
Thaelia anysia
11:7
Roelofa cf narga
11:9
Fatellalla fatella
Tarema fuscosa
11:8
Menevia lantona
12:1
Druentica partha
12:1
Procinnus cahureli
12:1
Ulmara rotunda

Plate 4

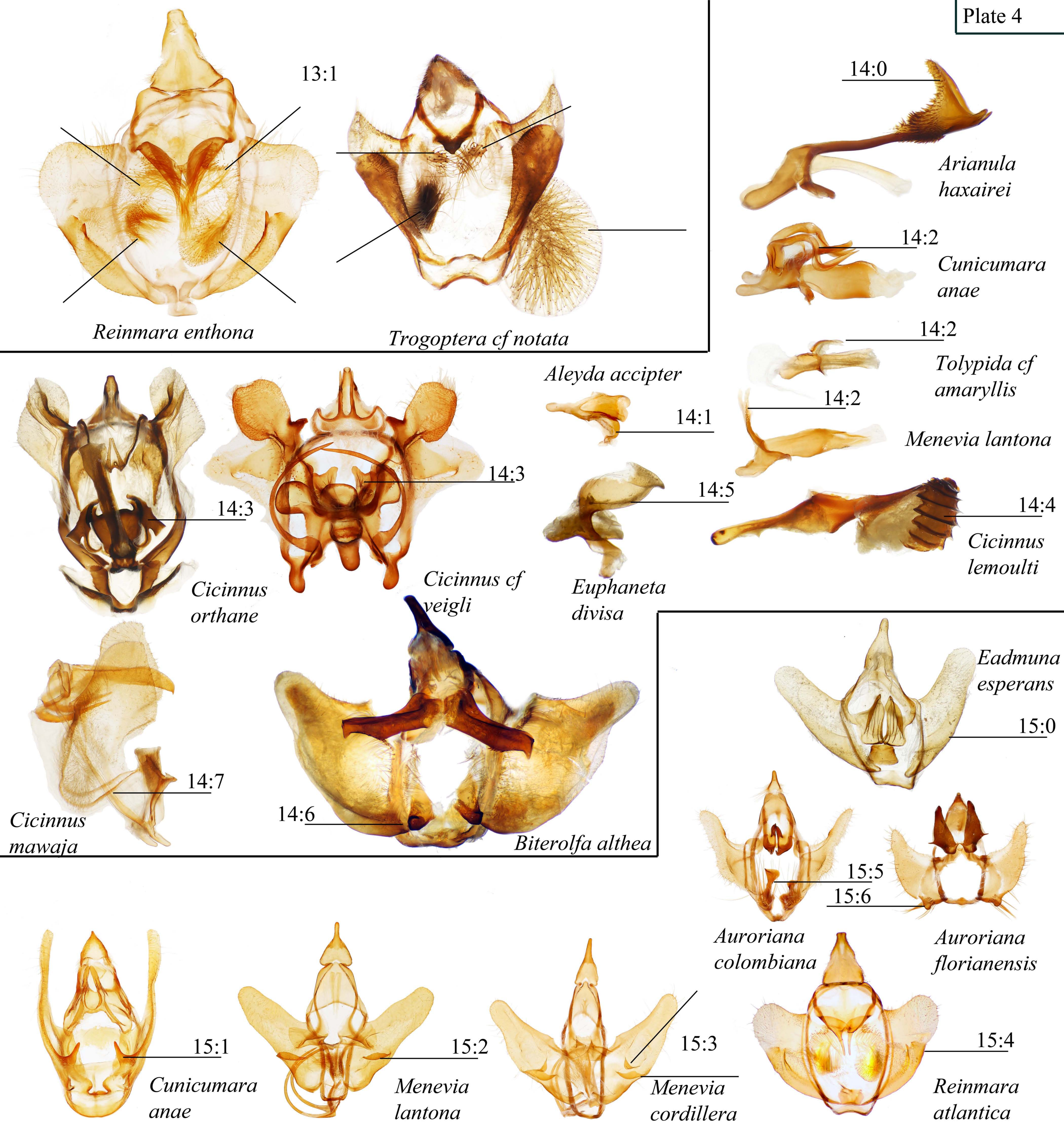

13:1
Reinmara enthona
Trogoptera cf notata
14:0
Arianula haxairei
14:2
Cunicumara anae
14:2
Tolypida cf amaryllis
14:2
Menevia lantona
Aleyda accipter
14:1
14:5
Euphaneta divisa
14:4
Cicinnus lemoulti
14:3
Cicinnus orthane
14:3
Cicinnus cf veigli
Eadmuna esperans
15:0
14:7
Cicinnus mawaja
14:6
Biterolfa althea
15:5
15:6
Auroriana colombiana
Auroriana florianensis
15:1
Cunicumara anae
15:2
Menevia lantona
15:3
Menevia cordillera
15:4
Reinmara atlantica

Plate 5

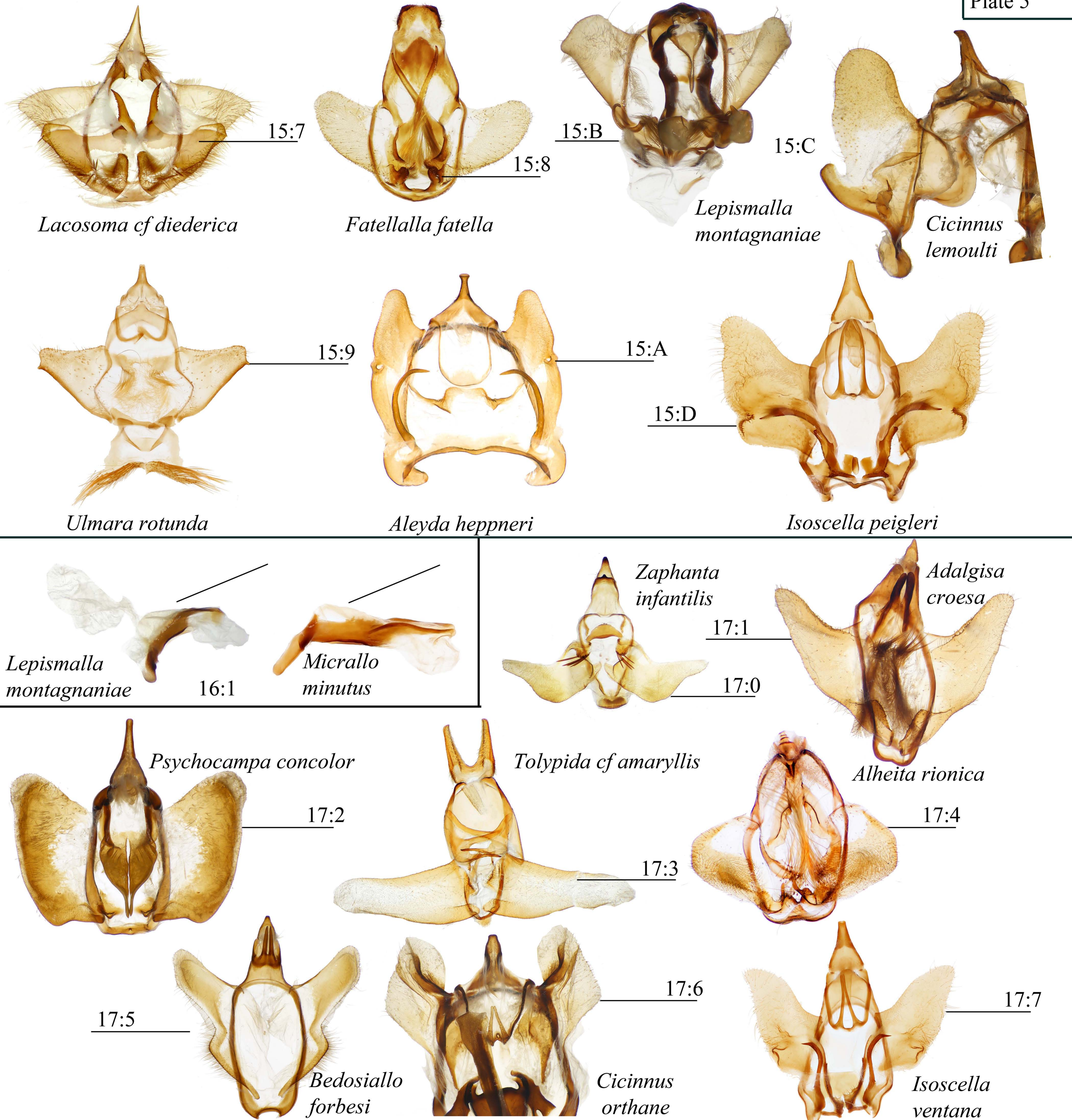
15:7
Lacosoma cf diederica
15:8
Fatellalla fatella
15:B
Lepismalla montagnaniae
15:C
Cicinnus lemoulti
15:9
Ulmara rotunda
15:A
Aleyda heppneri
15:D
Isoscella peigleri
Lepismalla montagnaniae
16:1
Micrallo minutus
Zaphanta infantilis
17:0
Adalgisa croesa
17:1
Psychocampa concolor
17:2
Tolypida cf amaryllis
17:3
Alheita rionica
17:4
17:5
Bedosiallo forbesi
17:6
Cicinnus orthane
17:7
Isoscella ventana

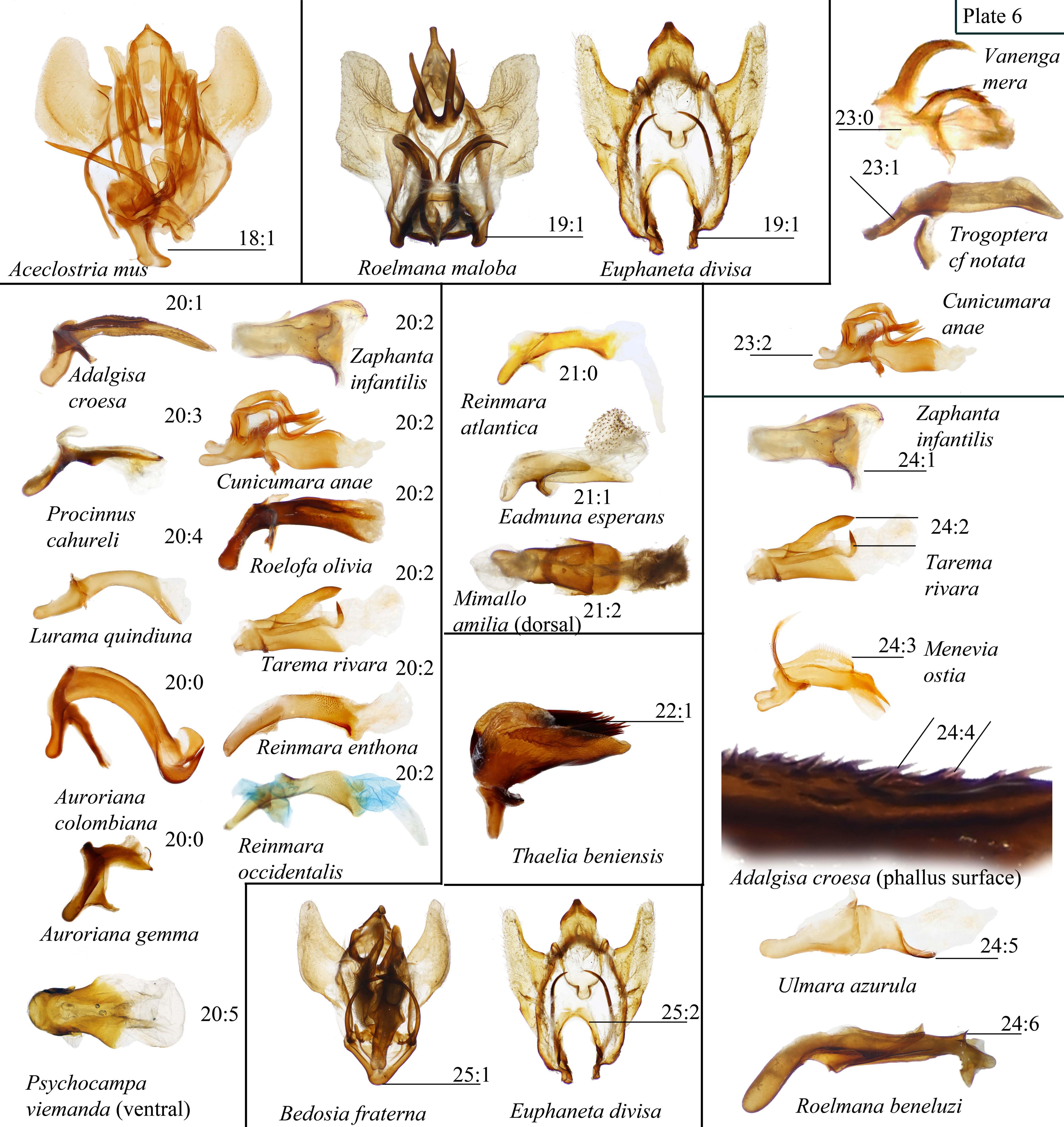
Plate 6
Aceclostria mus
18:1
Roelmana maloba
19:1
Euphaneta divisa
19:1
Vanenga mera
23:0
23:1
Trogoptera cf notata
Cunicumara anae
23:2
20:1
Adalgisa croesa
20:2
Zaphanta infantilis
20:3
Procinnus cahureli
20:2
Cunicumara anae
20:4
20:2
Roelofa olivia
Lurama quindiuna
20:2
Tarema rivara
20:2
20:0
Auroriana colombiana
Reinmara enthona
20:2
20:0
Reinmara occidentalis
Auroriana gemma
20:5
Psychocampa viemanda (ventral)
Reinmara atlantica
21:0
21:1
Eadmuna esperans
Mimallo amilia (dorsal)
21:2
22:1
Thaelia beniensis
Zaphanta infantilis
24:1
24:2
Tarema rivara
24:3
Menevia ostia
24:4
Adalgisa croesa (phallus surface)
Ulmara azurula
24:5
24:6
Roelmana beneluzi
25:1
Bedosia fraterna
25:2
Euphaneta divisa

Plate 7

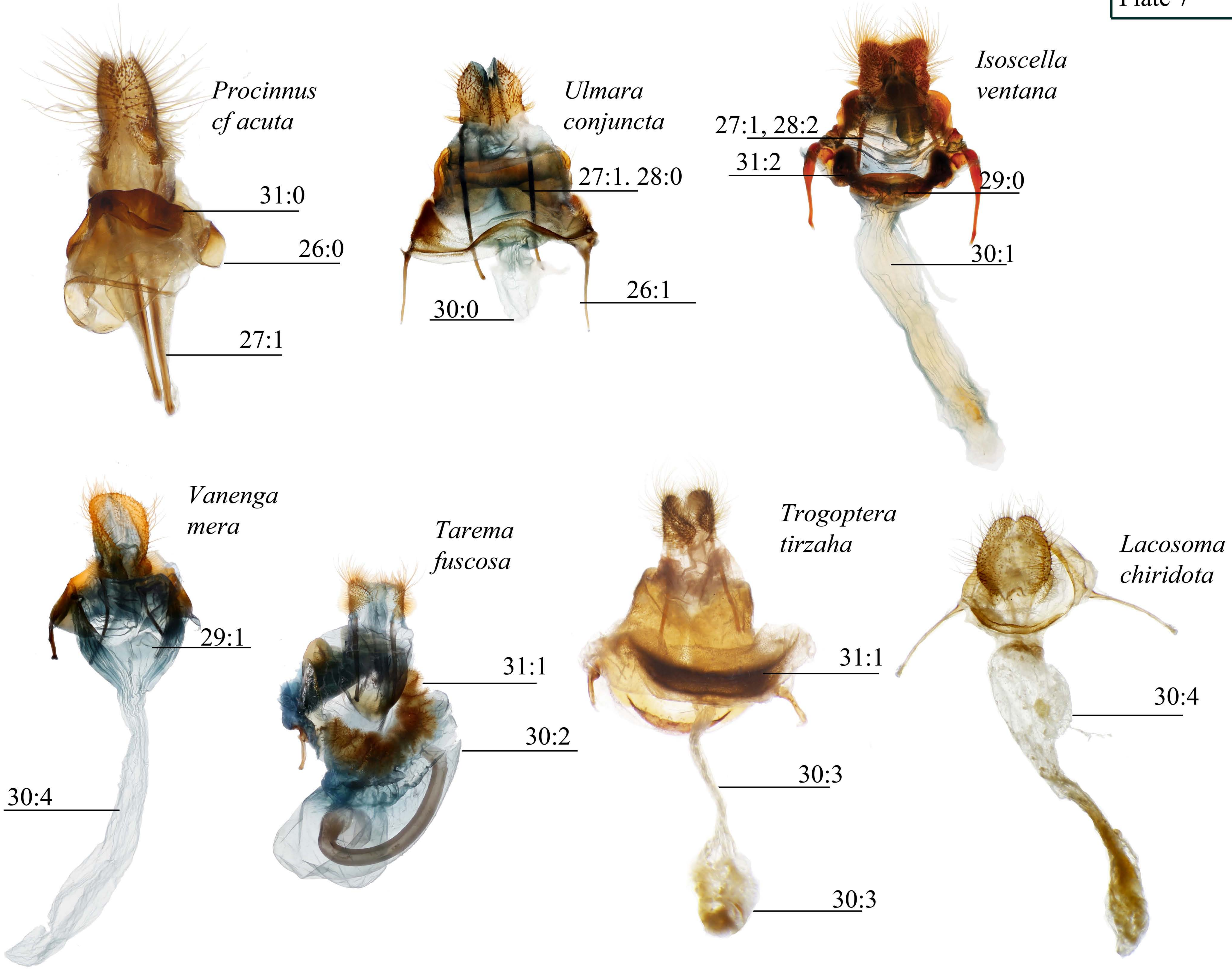

Procinnus cf acuta
31:0
26:0
27:1
Ulmara conjuncta
27:1. 28:0
30:0
26:1
Isoscella ventana
27:1, 28:2
31:2
29:0
30:1
Vanenga mera
29:1
30:4
Tarema fuscosa
31:1
30:2
Trogoptera tirzaha
31:1
30:3
30:3
Lacosoma chiridota
30:4

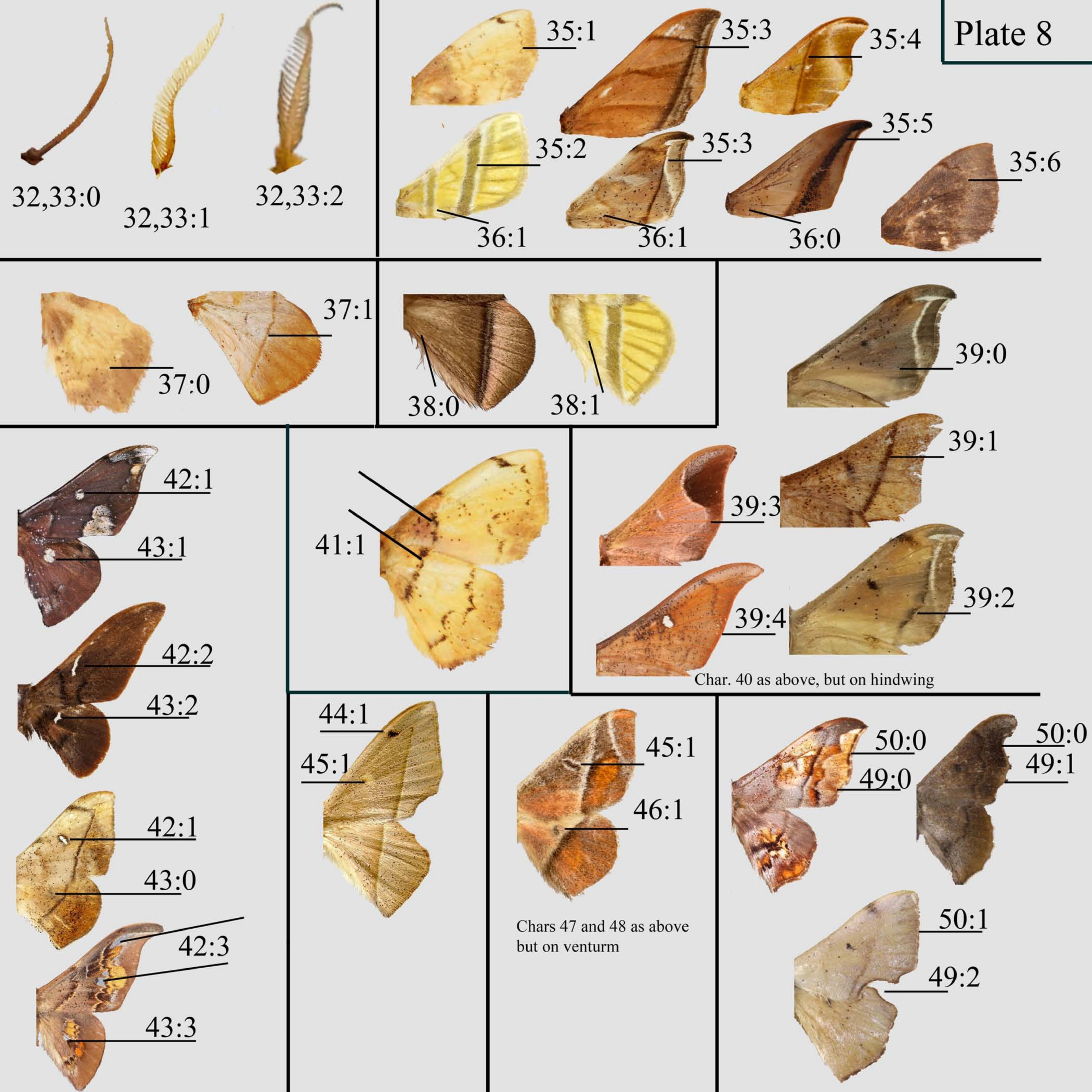
Plate 8
32,33:0
32,33:1
32,33:2
35:1
35:3
35:4
35:2
35:3
35:5
35:6
36:1
36:1
36:0
37:1
37:0
38:0
38:1
39:0
39:1
39:3
39:4
39:2
Char. 40 as above, but on hindwing
42:1
43:1
41:1
42:2
43:2
42:1
43:0
42:3
43:3
44:1
45:1
45:1
46:1
Chars 47 and 48 as above but on venturm
50:0
49:0
50:0
49:1
50:1
49:2

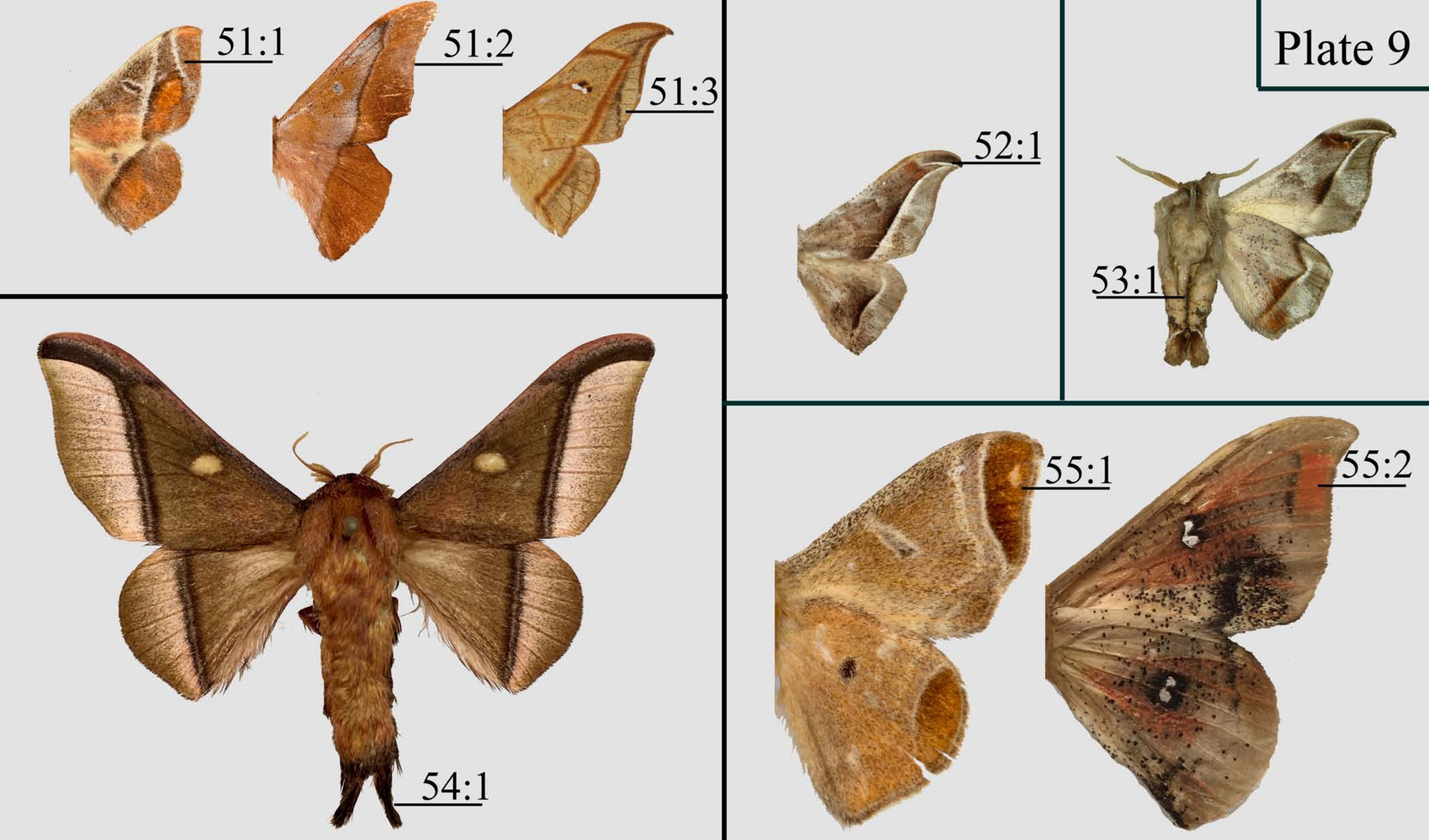

Plate 9
