# Supplementary figures and images for: Reclassification of the Sack-bearer Moths (Lepidoptera, Mimallonoidea, Mimallonidae)
Source: Zookeys. 2019 Jan 10;(815):1–114. doi: 10.3897/zookeys.815.27335 (PMC6336762; doi:10.3897/zookeys.815.27335)

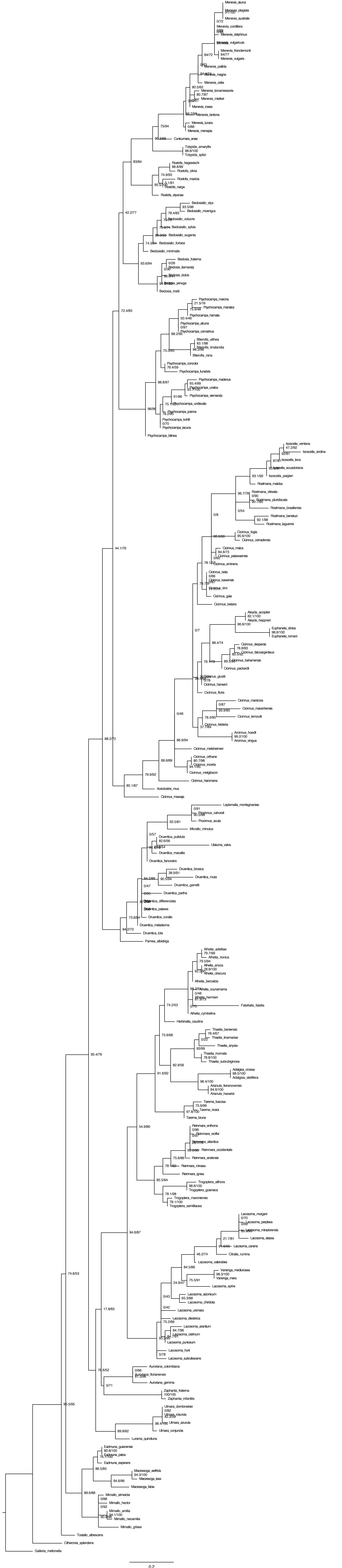

Supplement: Supplementary material 5 [file zookeys-815-001-s005.pdf]

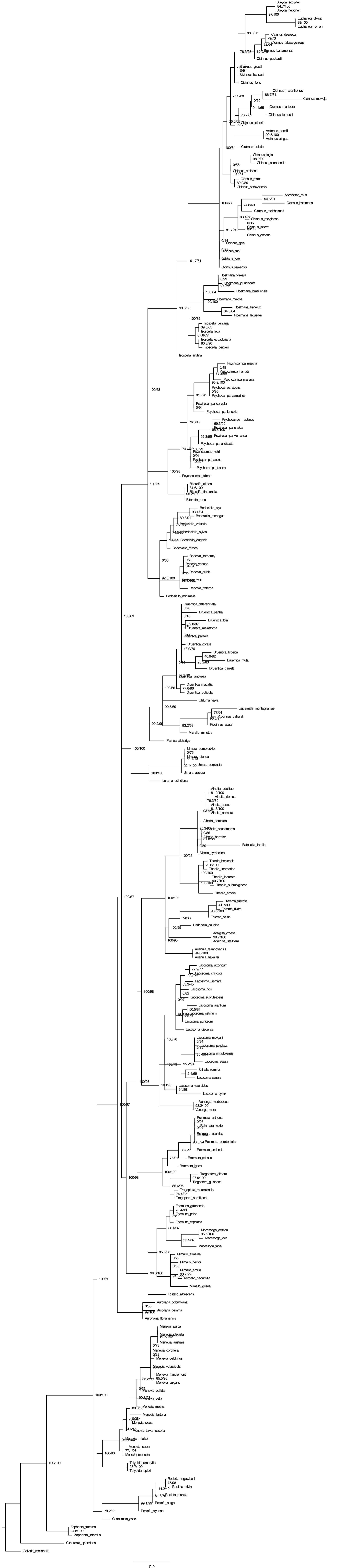

Supplement: Supplementary material 6 [file zookeys-815-001-s006.pdf]

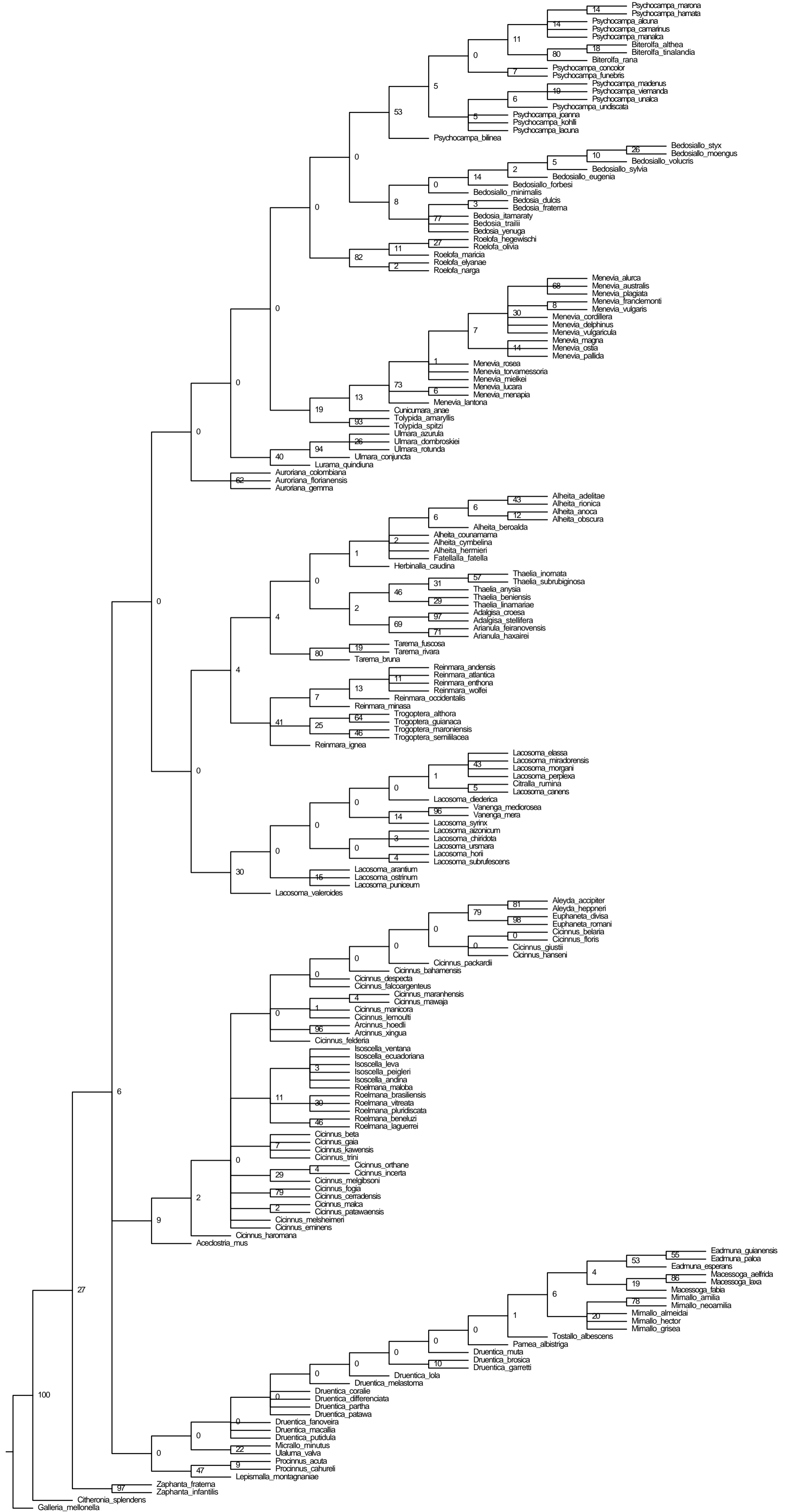

Supplement: Supplementary material 7 [file zookeys-815-001-s007.pdf]
